# The Effect of Nonreversibility on Inferring Rooted Phylogenies

**DOI:** 10.1093/molbev/msx294

**Published:** 2017-11-15

**Authors:** Svetlana Cherlin, Sarah E Heaps, Tom M W Nye, Richard J Boys, Tom A Williams, T Martin Embley

**Affiliations:** 1 Institute of Genetic Medicine, Newcastle University, Newcastle upon Tyne, United Kingdom; 2 School of Mathematics, Statistics and Physics, Newcastle University, Newcastle upon Tyne, United Kingdom; 3 School of Biological Sciences, University of Bristol, Bristol, United Kingdom; 4 Institute for Cell and Molecular Biosciences, Newcastle University, Newcastle upon Tyne, United Kingdom

**Keywords:** rooting, phylogenetic tree, substitution model, Bayesian hierarchical modeling

## Abstract

Most phylogenetic models assume that the evolutionary process is stationary and reversible. In addition to being biologically improbable, these assumptions also impair inference by generating models under which the likelihood does not depend on the position of the root. Consequently, the root of the tree cannot be inferred as part of the analysis. Yet identifying the root position is a key component of phylogenetic inference because it provides a point of reference for polarizing ancestor–descendant relationships and therefore interpreting the tree. In this paper, we investigate the effect of relaxing the unrealistic reversibility assumption and allowing the position of the root to be another unknown. We propose two hierarchical models that are centered on a reversible model but perturbed to allow nonreversibility. The models differ in the degree of structure imposed on the perturbations. The analysis is performed in the Bayesian framework using Markov chain Monte Carlo methods for which software is provided. We illustrate the performance of the two nonreversible models in analyses of simulated data using two types of topological priors. We then apply the models to a real biological data set, the radiation of polyploid yeasts, for which there is robust biological opinion about the root position. Finally, we apply the models to a second biological alignment for which the rooted tree is controversial: the ribosomal tree of life. We compare the two nonreversible models and conclude that both are useful in inferring the position of the root from real biological data.

## Introduction

The root of a phylogenetic tree is fundamental to its biological interpretation, providing a critical reference point for polarizing ancestor–descendant relationships and for determining the order in which key traits evolved along the tree (Embley and Martin 2006). Despite its importance, most models of sequence evolution are based on homogeneous continuous time Markov processes (CTMPs), which are assumed to be stationary and time-reversible, with the mathematical consequence that the likelihood of a tree does not depend on where it is rooted. Therefore, other methods are generally used to root evolutionary trees. The most common approach is to use an outgroup to the clade of interest, or ingroup; the root is then placed on the branch connecting the outgroup to the ingroup (Penny 1976; Huelsenbeck et al. 2002). However, this approach can be problematic if the outgroup is only distantly related to the ingroup because the long branch leading to the outgroup can induce phylogenetic artefacts such as long branch attraction (LBA), potentially interfering with the inference of ingroup relationships and the root position (Felsenstein 1978; Holland et al. 2003; Bergsten 2005). Indeed it has been proposed that the three domains of tree of life, in which Eukaryota represent the sister group to a monophyletic Archaea, could have resulted from LBA (Tourasse and Gouy 1999; Williams et al. 2013). Outgroup rooting is also difficult to apply to the question of rooting the universal tree, for which no obvious outgroup is available. One solution to this problem has been to use pairs of paralogous genes that diverged from each other before the last common ancestor of all cellular life, so that one paralogue can be used to root a tree of the other (Iwabe et al. 1989; Brown and Doolittle 1995; Baldauf et al. 1996; Hashimoto and Hasegawa 1996). However, for any given gene it is difficult to unambiguously establish that duplication took place before the divergence of the domains of life. The number of genes to which this technique can be applied is also limited.

An alternative, but perhaps underexplored, approach to rooting trees is to take a model-based approach. Focusing on homogeneous CTMPs, it is helpful to distinguish between the ideas of *homogeneity*, *stationarity*, and *reversibility* (e.g., see Yang 2006, Chapter 1).We say that a model is *homogeneous* if the evolutionary process at the root of the tree and across all branches can be characterized by a single instantaneous rate matrix. A homogeneous model is termed *reversible* if the rate matrix on which it depends can be factorized into a diagonal matrix of stationary probabilities and a symmetric matrix of exchangeability parameters. The latter determines the general propensity for change between the different pairs of molecular units (Whelan and Goldman 2001). Similarly, we call a rate matrix *reversible* if it permits such a factorization. Finally, a CTMP is *stationary* if the probability of a site being occupied by each molecular unit (e.g., each nucleotide for DNA) does not change over time and the probabilities of transitioning between units over some time interval depend only on the size of that interval and not on its position in time. It follows that all nonstationary models are also nonhomogeneous, although the converse need not be true.

The assumptions of stationarity and reversibility at the heart of standard substitution models simplify the underpinning mathematics and are usually justified on the grounds of computational convenience, rather than biological reasoning. Indeed, there is frequently evidence of nonreversibility in biological data sets (Squartini and Arndt 2008; Woodhams et al. 2015), whereas the assumption of stationarity is often undermined by variation in GC-content across species (Foster 2004; Cox et al. 2008). These unrealistic assumptions also come at an inferential cost, generating likelihood functions that are invariant to the position of the root. Therefore, a model which relaxes one or both assumptions can not only offer more biological credibility, but also give rise to likelihood functions that depend on the position of the root, providing a model-based tool for rooting phylogenetic trees. Most models that allow root inference are nonhomogeneous, typically assigning different reversible rate matrices to different parts of the tree. Generally, these models are nonstationary and allow variation in the theoretical stationary distribution over time. Some also allow variation in the exchangeability parameters (Dutheil and Boussau 2008) although, more commonly, they are fixed across all branches. For example, Yang and Roberts (1995) assigned common exchangeabilities but a different composition vector to each edge of the tree. Heaps et al. (2014) fitted a similar model in a Bayesian framework, but adopted a prior over composition vectors that allowed information to be shared between branches. Although biologically persuasive, such nonhomogeneous models are, however, highly parameterized and efforts have been made to seek more parsimonious representations. Yang and Roberts (1995) and Foster (2004) considered models in which composition vectors are applied to groups of edges rather than to a single edge. Blanquart and Lartillot (2006) used a variation of this idea by assuming the compositional shifts occurred according to a Poisson process, independently of speciation events. In the context of nucleotide evolution, Galtier and Gouy (1998) reduced the number of parameters in the model of Yang and Roberts (1995) by using a model parameterized by a single G + C component, rather than three free parameters for the composition vector. But this inevitably came at the cost of a loss of information from the alignment. In a general setting that allowed different reversible or nonreversible rate matrices to be assigned to each edge of the tree, Jayaswal et al. (2011) devised a heuristic to reduce the number of rate matrices using the distances between them as a similarity criteria and forcing the most similar rate matrices to be identical. However, given the speculative nature of the model search, the algorithm offered no assurance of identifying a global optimum. In spite of these moves, efforts to reduce the number of parameters, nonhomogeneous models remain substantially more highly parameterized than their homogeneous counterparts. This makes model-fitting computationally challenging, often limiting inference to fixed unrooted trees (e.g., Dutheil and Boussau 2008; Jayaswal et al. 2011) or alignments on a small number of taxa (e.g., Heaps et al. 2014). In this paper, we take a Bayesian approach to inference and focus on rooting using a *homogeneous* and stationary, but nonreversible, model that requires only *one* rate matrix. We develop a Markov chain Monte Carlo (MCMC) algorithm for posterior inference and provide an associated software implementation. This nonreversible model has previously been explored by Huelsenbeck et al. (2002), however we build on that work in a number of ways. First, Huelsenbeck et al. (2002) used a so-called noninformative prior on the rate matrix, with independent uniform distributions for each off-diagonal element. We incorporate prior structure and consider two hierarchical priors that are centered on a standard reversible rate matrix but allow nonreversible perturbations of the individual elements. Our two priors differ in the structure of the perturbation. Additionally, we do not fix the unrooted topology and extend the inferential algorithm to allow inference of rooted trees. This enables us to present a more complete summary of the posterior over root positions and to demonstrate the sensitivity of the analysis to different topological priors. Finally, although Huelsenbeck et al. (2002) only considered small alignments of up to nine taxa, we consider more compelling analyses with data sets of up to 36 taxa. To our knowledge, model-fitting software supporting other nonstationary or nonreversible models from the literature cannot be used routinely to learn simultaneously about *both* the unrooted topology and root position of non-clock trees for data sets of this size. We test our hierarchical models on simulated data and on a real biological data set for which there is robust biological opinion about the position of the root. Finally, we apply the models to an open question in biology: the root of the tree of life.

## New Approaches

### Top-Level Model Description

We consider a number of aligned homologous sequences and aim to infer the evolutionary relationships among these sequences. These relationships can be described in the form of a bifurcating tree, where each edge represents the period of time over which substitutions accumulate, and each bifurcation represents a speciation event. The nucleotides at each site of a sequence alignment on *n* taxa can be thought of as independent realizations of a random variable $X={\left(x_{1},\ldots ,x_{n}\right)}^{T}$ on a discrete space where $x_{i}\in \Omega$ and Ω={A,G,C,T}, for i=1,…,n. The evolutionary process operating along each edge of the tree is described by a homogeneous CTMP, where the future value of a nucleotide at any given site depends on its current value only and does not depend on its past values given this current value, that is

$$\begin{array}{c} \mathrm{Pr}(X(t)=j|X(t_{1})=i_{1},X(t_{2})=i_{2},\ldots ,X(t_{n})=i_{n})\\ =\mathrm{Pr}(X(t)=j|X(t_{n})=i_{n}), \end{array}$$

where $t>t_{n}>t_{n-1}>\ldots >t_{2}>t_{1}$. The process can therefore be specified by a transition matrix $P(\ell )=\{p_{ij}(\ell )\}$ whose elements $p_{ij}(\ell )$ represent the probabilities of changing from one nucleotide to another over a branch of length *ℓ*. Equivalently we can represent the process through an instantaneous rate matrix *Q*, where P(ℓ)= exp ⁡(Qℓ). The off-diagonal elements of *Q* represent an instantaneous rate of change from one nucleotide to another during an infinitesimal period of time. The diagonal elements are specified so that every row sums to zero. If branch lengths need to be expressed in terms of expected number of substitutions per site, then the *Q* matrix has to be rescaled so that $-\sum Q_{ii}\pi_{Q,i}=1$, where $\pi_{Q}=(\pi_{Q,A},\pi_{Q,G},\pi_{Q,C},\pi_{Q,T})$ is the theoretical stationary distribution of the process, which can be calculated from *Q* (e.g., see Huelsenbeck et al. 2002).

Most phylogenetic models are time-reversible. Reversibility implies that

$$\pi_{Q,i}p_{ij}=\pi_{Q,j}p_{ji}$$

and allows the rate matrix to be represented in the form Q=SΠ, where *S* is a symmetric matrix containing the exchangeability parameters *ρ_ij_*, i≠j, as the off-diagonal elements with $\rho_{ij}=\rho_{ji}$, and $\Pi =\text{diag}(\pi_{Q})$ is a diagonal matrix containing the elements of $\pi_{Q}$. Although the reversibility assumption makes statistical models simpler, it has no biological justification and is applied for computational convenience only. Indeed, there is often evidence of nonreversibility in biological data sets (Squartini and Arndt 2008; Woodhams et al. 2015).

The most common reversible rate matrix, with six exchangeability parameters, is the general time-reversible (GTR) model (Tavaré 1986). The HKY85 model (Hasegawa et al. 1985) is a widely used special case with only two distinct *ρ_ij_*, one of which is fixed to prevent arbitrary rescaling of the *Q* matrix. The rate matrix *Q* of this model is then specified by the compositional frequency vector $\pi =(\pi_{A},\pi_{G},\pi_{C},\pi_{T})$ and by the transition–transversion rate ratio *κ* as

$$Q=\left(\begin{array}{cccc} {}* & \kappa \pi_{G} & \pi_{C} & \pi_{T}\\ \kappa \pi_{A} & * & \pi_{C} & \pi_{T}\\ \pi_{A} & \pi_{G} & * & \kappa \pi_{T}\\ \pi_{A} & \pi_{G} & \kappa \pi_{C} & * \end{array}\right).$$

Here the symbol * is used to indicate that the diagonal elements are specified such that every row sums to zero.

We consider two Bayesian hierarchical models that are both nonreversible and based on an unstructured rate matrix *Q* whose 12, distinct off-diagonal elements *q_ij_* are unconstrained in ${\mathbb{R}}_{+}^{12}$. The models differ in the prior they assign to these off-diagonal elements. In each case the prior treats each *q_ij_* as a log-normal perturbation of the corresponding element of the unknown rate matrix of a HKY85 model. The first hierarchical model, henceforth called the NR (nonreversible) model, utilizes one perturbation component, whereas the more complex model, henceforth called the NR2 model, utilizes two perturbation components. The variances of the perturbations are unknown and can provide a measure of the evidence of nonreversibility in the data. In both models we assume that the variation between the overall rate of substitution events at sites can be modeled by a discrete gamma distribution with four rate categories and shape parameter *α* (Yang 1994).

### Top-Level Prior Distribution

#### NR Model

We denote the off-diagonal elements of the rate matrix of the NR model by *q_ij_*, and the off-diagonal elements of the rate matrix of the HKY85 model by $q_{ij}^{H},\;i\neq j$, so for instance $q_{12}^{H}=\kappa \pi_{G}$. The nonreversibility of the NR model is achieved by a log-normal perturbation of the off-diagonal elements of the rate matrix *Q^H^* using a perturbation component *σ* as represented in the following directed acyclic graph (DAG):


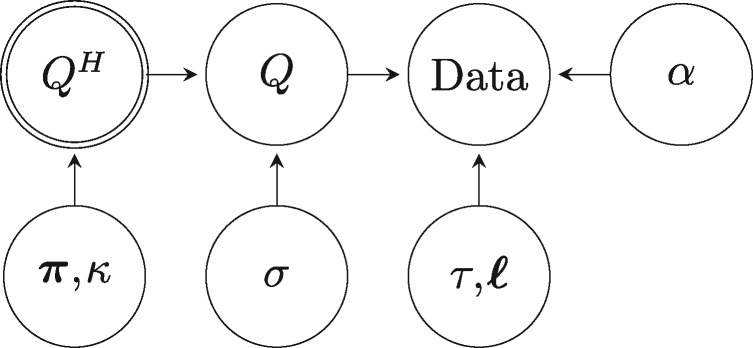


DAGs are a useful way of representing (especially hierarchical) models graphically (e.g., see Spiegelhalter and Lauritzen 1990). In a DAG, the nodes represent random variables and the directed arrows are used to indicate the order of conditioning when factorizing the joint probability density of all the nodes. A double circle around a node indicates deterministic dependence; in this case *Q^H^* is completely determined once π and *κ* are known. In the DAG above, *α* is the across-site heterogeneity parameter, *τ* is the rooted topology, and ℓ are the branch lengths.

Working element-wise on a log scale, the off-diagonal elements of the rate matrix of the NR model can be expressed as, for i≠j

$$\text{log}\;q_{ij}=\;\text{log}\;q_{ij}^{H}+\epsilon_{ij},$$

where the $\epsilon_{ij}$ are independent $N(0,\sigma^{2})$ quantities. Here the perturbation standard deviation *σ* represents the extent to which *Q* departs from a HKY85 structure: the larger its value, the greater the degree of departure. This parameter is treated as an unknown quantity whose value we learn about during the analysis. The unknowns of the hierarchical model therefore comprise: the composition vector π, the transition–transversion rate ratio *κ*, the perturbation standard deviation *σ*, the off-diagonal elements of the rate matrix *Q*, the shape parameter *α*, the branch lengths ℓ, and the rooted topology *τ*. We express our initial uncertainty about these unknown parameters though a prior distribution that takes the form

(1) π(π,κ,σ,Q,α,ℓ,τ)=π(Q|π,κ,σ)π(π,κ,σ,α,ℓ,τ)

in which the top-level prior density π(Q|π,κ,σ) has been described above. The bottom-level density π(π,κ,σ,α,ℓ,τ) will be described in Bottom-Level Prior Distribution section.

#### NR2 Model

Under the NR model, departures from HKY85 structure could lead to a nonreversible model or simply a GTR rate matrix. As such the two types of deviation are confounded and so for any given data set, learning that *σ* is large does not necessarily provide evidence of nonreversibility. The NR2 model addresses this issue, thereby aiding model interpretation, by using a two-stage process to perturb the underlying HKY85 rate matrix *Q^H^*. The first perturbation is within the space of GTR matrices, perpendicular to the subspace of HKY85 matrices, leading to a reversible rate matrix denoted *Q^R^*. The second perturbation acts on *Q^R^* and is within the space of general rate matrices but perpendicular to the subspace of GTR matrices, leading to a general nonreversible rate matrix denoted *Q*. These two random perturbations have different variance parameters $\sigma_{R}^{2}$ and $\sigma_{N}^{2}$, respectively. Biologically, the variance parameter $\sigma_{R}^{2}$ represents the extent to which the data contradict the assumption of a common rate of transition and a common rate of transversion. Similarly, the variance parameter $\sigma_{N}^{2}$ provides a measure of the evidence in the data for the directionality of time.

The general structure of this model can be represented by the following DAG:


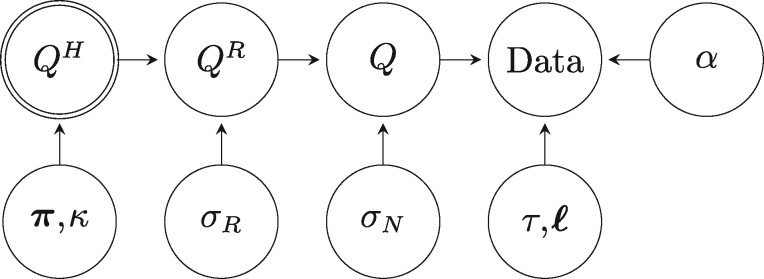


The two-stage perturbation procedure is explained further in Appendix A. Therefore, the unknown parameters in the NR2 model are the composition vector π, the transition–transversion rate ratio *κ*, the perturbation standard deviation on the reversible plane *σ_R_*, the perturbation standard deviation on the nonreversible plane *σ_N_*, the shape parameter *α*, the branch lengths ℓ, and the rooted topology *τ*. We also have latent variables comprising $\nu_{1},\ldots ,\nu_{5}$ for the reversible perturbation and $\eta_{1},\eta_{2},\eta_{3}$ for the nonreversible perturbation (see Appendix A). The prior distribution of these unknowns takes the form

(2) $$\begin{array}{cc} \pi & (\pi ,\kappa ,\sigma_{R},\sigma_{N},\nu ,\eta ,\alpha ,\ell ,\tau )\\ & =\pi (\nu |\sigma_{R})\pi (\eta |\sigma_{N})\pi (\pi ,\kappa ,\sigma_{R},\sigma_{N},\alpha ,\ell ,\tau ) \end{array},$$

where the top-level prior distributions with densities $\pi (\nu |\sigma_{R})$ and $\pi (\eta |\sigma_{N})$ are $\nu_{i}∼N(0,\sigma_{R}^{2})$ for i=1,…,5 independently, and $\eta_{i}∼N(0,\sigma_{N}^{2})$ for i=1,2,3 independently (see Appendix A). The bottom-level density $\pi (\pi ,\kappa ,\sigma_{R},\sigma_{N},\alpha ,\ell ,\tau )$ will be described in the following section.

### Bottom-Level Prior Distribution

#### NR Model

The bottom-level prior density π(π,κ,σ,α,ℓ,τ) from equation (1) takes the form

π(π,κ,σ,α,ℓ,τ)=π(π)π(κ)π(σ)π(α)π(ℓ)π(τ)

to reflect our initial assessment of independence between these parameter blocks.

The composition vector π is defined on the four-dimensional simplex, that is, it has four positive elements, constrained to sum to one. We choose to assign it a Dirichlet prior, $\pi ∼D(a_{\pi }\pi_{0})$, where $\pi_{0}=(0.25,0.25,0.25,0.25)$ is the mean and $a_{\pi }$ is a concentration parameter (we take $a_{\pi }=4$). This prior is exchangeable with respect to the nucleotide labels, representing the belief that on average the number of different nucleotides in a sequence is the same. We adopt a log-normal prior for the transition–transversion rate ratio $\kappa ∼LN(\text{log}\;\kappa_{0},\nu^{2})$, where *κ*_0_ = 1 and *ν* = 0.8. The parameters of the prior for *κ* represent our belief that the probability of *κ* exceeding 2 is 0.2, that is, Pr⁡(κ<2)=0.8. This was informed by our experiences of working with the HKY85 model; we judged that in around 80% of cases, the transition–transversion rate ratio was less than 2. The perturbation parameter *σ* is assigned an Exponential prior σ∼ Exp(γ), where the rate γ=2.3 reflects our prior belief that the probability of *σ* exceeding 1 is 0.1, that is, Pr⁡(σ<1)=0.9. Together with the rest of our hierarchical specification, this choice induces a prior for the stationary distribution $\pi_{Q}$ in which little density is assigned to vectors where some characters are heavily favored over the others.

The branch lengths are assigned independent Exponential priors $\ell_{i}∼\;\text{Exp}(\mu )$, where i=1,…,k and *k* is the number of edges. The rate *μ* equals 10, so that $E(\ell_{i})=0.1$, representing the belief that, on average, there will be 0.1 substitutions per site. The shape parameter *α* is assigned a gamma prior, α∼Ga(10,10), which ensures the expected substitution rate in the Ga(α,α) model for site-specific substitution rates is modestly concentrated around 1. We define a *root type* as the number of species on each side of the root. For example, the root type 1:(n−1) represents a root split on a pendant edge, 2:(n−2) represents a root split between two taxa and all others, etc. The set $T_{n}$ of all rooted trees on *n* species can be expressed as a partition

$$T_{n}=\overset{k_{n}}{\underset{i=1}{\cup }}T_{i:n-i}\text{where}k_{n}=\{\begin{array}{cc} (n-1)/2,\; & \text{if}n\;\text{odd},\\ n/2,\; & \text{if}n\;\text{even} \end{array}$$

in which $T_{i:n-i}$ represents the subset of rooted trees whose root type is i:n−i. A uniform prior over rooted topologies assigns a prior probability of more than 0.5 to the set $T_{1:n-1}$, in other words, to trees with roots on pendant edges. We felt that trees with deeper roots are generally more biologically plausible and should be assigned higher prior mass, although still retaining a diffuse initial distribution. We therefore chose to assign the rooted topology a prior according to the Yule model of speciation, which assumes that at any given time each of the species is equally likely to undergo a speciation event. This generates a biologically defensible prior in which each subset $T_{i:n-i}$ receives the same prior probability if *n* is odd. If *n* is even, a near uniform distribution is induced, but with the subset $T_{n/2:n/2}$ receiving half the prior probability of the others. This is illustrated in supplementary figure 1, Supplementary Material online, which compares the uniform and Yule priors for rooted trees on *n* = 4 taxa, showing the probability assigned to the subsets $T_{1:3}$ and $T_{2:2}$, and to every possible rooted tree.

The probability of generating a *n*-species tree T under the Yule distribution is calculated by dividing the number of labelled histories for the tree T by the total number of all possible labelled histories on *n* species (Steel and McKenzie 2001). This probability depends on the complete rooted topology and therefore has to be recalculated at every iteration of the Metropolis–Hastings algorithm used for inference. To save computational time, we therefore additionally introduce an approximation to the Yule prior, which we term the *structured uniform prior*, that assigns equal prior probability to all subsets $T_{1:n-1},\ldots ,T_{k_{n}:n-k_{n}}$ of rooted trees. To sample a rooted topology from this distribution, we first sample a subset $T_{i:n-i}$ uniformly. This subset contains all the trees with root type i:n−i. We then sample uniformly from the rooted trees within $T_{i:n-i}$. Computationally, this prior is more convenient than the Yule prior because its mass function is independent of the particular unrooted topology and only depends on the root type; see supplementary figure 1, Supplementary Material online, for an illustration with trees on *n* = 4 taxa. It also has the advantage of being uniform over the subsets of the partition $T_{i:n-i}$ for *any* value of *n*. Posterior sensitivity to the choice of topological prior will be discussed in Analysis of Experimental Data section.

#### NR2 Model

The bottom-level prior density

$\pi (\pi ,\kappa ,\sigma_{R},\sigma_{N},\alpha ,\ell ,\tau )$ from equation (2) takes the form

$$\pi (\pi ,\kappa ,\sigma_{R},\sigma_{N},\alpha ,\ell ,\tau )=\pi (\pi )\pi (\kappa )\pi (\sigma_{R})\pi (\sigma_{N})\pi (\alpha )\pi (\ell )\pi (\tau ).$$

The rate heterogeneity parameter *α*, branch lengths ℓ, rooted topology *τ*, and the parameters π and *κ* of the reversible *Q^H^* matrix are assigned the same priors as those used for the NR model. Both perturbation standard deviations are assigned the same prior as their analogue, *σ*, in the NR model, that is, $\sigma_{R}∼\;\text{Exp}(2.3)$ and $\sigma_{N}∼\;\text{Exp}(2.3)$.

## Results

Taking a Bayesian approach to inference, we fitted the NR and NR2 models to the data sets described in this section using an MCMC algorithm. Full details of the inferential procedure are provided in Materials and Methods section.

### Analysis of Simulated Data

Our simulations aim to explore the effect of three factors on root inference: 1) different levels of nonreversibility in the evolutionary process, 2) different topologies and branch lengths, and 3) different levels of (unmodeled) nonstationarity in the evolutionary process.

#### Different Levels of Nonreversibility in the Evolutionary Process

Here, we explore the posterior when the NR and NR2 models are fitted to data that contain different levels of nonreversibility.

##### Simulation of Data

The tree used to simulate the data is a random 30-taxon tree (generated under the Yule birth process), with the branch lengths drawn as independent samples from a Ga(2, 20) distribution. The lengths of the branches adjacent to the root are independent samples from a Ga(1, 20) distribution such that their combined length is Ga(2, 20) (supplementary fig. 2, Supplementary Material online). This ensures that the lengths of all edges on the underlying unrooted topology are statistically indistinguishable.

Under the NR model, the perturbation from the underlying reversible HKY85 rate matrix *Q^H^* does not necessarily produce a nonreversible rate matrix *Q*. It follows that the perturbation parameter *σ* in the NR model does not provide a direct measure of the degree of nonreversibility. In contrast, because the NR2 model decomposes the perturbation into its reversible and nonreversible parts, the nonreversible perturbation parameter *σ_N_* in the NR2 model gives a more clear-cut measurement. To assess the performance of both models under a broad and clearly demarcated set of nonreversibility conditions, we therefore simulated data using the NR2 model. To this end we first fixed the base HKY85 rate matrix *Q^H^* using the values π=(0.25,0.25,0.25,0.25) and *κ* = 2. In all cases we used the same value for the reversible perturbation, $\sigma_{R}=0.1$, but investigated five different values of the nonreversible perturbation standard deviation *σ_N_*: *σ_N_* = 0, 0.1, 0.25, 0.5, 1.0. To simulate the alignments, we used our own software, programmed in Java, which is available in the Supplementary Material online.

For each value of *σ_N_*, we simulated five different rate matrices *Q*, then for each rate matrix we simulated five different alignments of length 2,000 bp. Simulated alignments from the same rate matrix are clearly samples from a process with the same stationary distribution $\pi_{Q}$, whereas the simulations using different rate matrices sampled from the same value of *σ_N_* come from processes with different stationary distributions. This type of alignment simulation therefore allows us to investigate different sources of variability in the data as the degree of nonreversibility increases. All the alignments were simulated using a gamma shape heterogeneity parameter generated from Ga(10,10). Note that the case of $\sigma_{N}=0$ corresponds to the reversible GTR model. The other values of *σ_N_* were chosen so that the prior for the stationary distribution induced by the log-normal perturbation would be in the range of values estimated for real data; as *σ_N_* increases, significant support is given to highly biased compositions, and for $\sigma_{N}>1.0$ these are biologically unrealistic (supplementary fig. 3, Supplementary Material online).

A measure of nonreversibility used elsewhere in the literature (e.g., Huelsenbeck et al. 2002; Squartini and Arndt 2008) is Huelsenbeck’s *I* statistic, defined as $I=\sum_{ij}|\pi_{i}q_{ij}-\pi_{j}q_{ji}|$. Under a reversible model, $\pi_{i}q_{ij}=\pi_{j}q_{ji}$ for all i≠j, and so *I* = 0. However, *I* is strictly positive for nonreversible models, with larger values indicating a greater degree of nonreversibility. The values of Huelsenbeck’s *I* statistic for the models used to generate the data in these experiments are shown in table 1.

**Table 1.** Values of Huelsenbeck’s *I* Statistic for the *Q* Matrices Used in the Simulations.

Data Sets	*σ_N_* = 0	*σ_N_* = 0.1	*σ_N_* = 0.25	*σ_N_* = 0.5	*σ_N_* = 1.0
1a–1e	0.0000	0.0550	0.2327	0.3282	1.0416
2a–2e	0.0000	0.0366	0.1871	0.4423	0.9019
3a–3e	0.0000	0.0737	0.3297	0.4699	0.7494
4a–4e	0.0000	0.0538	0.1675	0.3654	0.7282
5a–5e	0.0000	0.1012	0.3541	0.4402	0.9948

Note.—By design, there is a strong positive correlation between *σ_N_* and *I*.

##### NR Model

Table 2 summarizes the marginal posterior probabilities of the correct root split and the posterior means for Huelsenbeck’s *I* statistic for the analyses using the NR model under the Yule prior (the posterior distributions of the root splits from a representative sample of simulations are shown in supplementary fig. 4, Supplementary Material online). When $\sigma_{N}=0$ the posterior of the root splits is identical to the prior (not shown) because the data contain no information about the root. As *σ_N_* increases, the root is inferred substantially better, with $\sigma_{N}=1.0$ demonstrating the best root inference of all analyzed values of *σ_N_*. However, the analyses of the 25 simulated data sets for each value of *σ_N_* do not show identical behavior. There is clearly variability between the data sets, although this is less noticeable for alignments simulated with the same rate matrix. For smaller values of *σ_N_*, the true root split is not inferred well in all experiments. However, as the degree of nonreversibility increases, the signal from the data becomes more consistent and markedly stronger. The true unrooted topology is also inferred with posterior probability close to one in most cases (supplementary fig. 5, Supplementary Material online). Altogether, this suggests that in addition to inferring the unrooted topology, we can also use the NR model to extract some information about the root.

**Table 2.** Marginal Posterior Probabilities of the Correct Root Split for the Simulations from the NR2 Model, Analyzed under the NR Model with the Yule Prior.

Data Set	*σ_N_* = 0	*σ_N_* = 0.1	*σ_N_* = 0.25	*σ_N_* = 0.5	*σ_N_* = 1.0
1a	0.06 (0.04)	0.11 (0.05)	0.62 (0.22)	0.95 (0.35)	1.00 (1.07)
1b	0.06 (0.04)	0.10 (0.06)	0.55 (0.22)	0.78 (0.34)	1.00 (1.07)
1c	0.07 (0.02)	0.16 (0.06)	0.31 (0.21)	0.56 (0.34)	1.00 (1.04)
1d	0.08 (0.02)	0.07 (0.05)	0.61 (0.21)	0.92 (0.31)	1.00 (1.09)
1e	0.10 (0.03)	0.10 (0.04)	0.85 (0.27)	0.72 (0.35)	1.00 (1.06)
2a	0.10 (0.04)	0.08 (0.05)	0.07 (0.15)	0.86 (0.41)	0.93 (0.81)
2b	0.06 (0.05)	0.11 (0.05)	0.56 (0.16)	0.97 (0.43)	1.00 (0.90)
2c	0.10 (0.03)	0.20 (0.06)	0.54 (0.18)	0.64 (0.49)	1.00 (0.92)
2d	0.09 (0.04)	0.08 (0.03)	0.20 (0.19)	0.89 (0.45)	1.00 (0.87)
2e	0.09 (0.02)	0.12 (0.05)	0.41 (0.18)	0.98 (0.45)	1.00 (0.86)
3a	0.04 (0.05)	0.13 (0.08)	0.20 (0.36)	0.28 (0.49)	0.98 (0.75)
3b	0.09 (0.04)	0.16 (0.05)	0.49 (0.31)	0.99 (0.49)	1.00 (0.74)
3c	0.06 (0.05)	0.11 (0.04)	0.68 (0.33)	0.92 (0.44)	0.94 (0.78)
3d	0.07 (0.03)	0.09 (0.03)	0.35 (0.32)	0.99 (0.45)	0.99 (0.73)
3e	0.12 (0.04)	0.17 (0.04)	0.79 (0.32)	0.97 (0.45)	1.00 (0.72)
4a	0.06 (0.06)	0.08 (0.04)	0.10 (0.20)	0.64 (0.33)	1.00 (0.76)
4b	0.08 (0.02)	0.06 (0.03)	0.13 (0.16)	0.19 (0.38)	1.00 (0.80)
4c	0.07 (0.06)	0.08 (0.03)	0.21 (0.17)	0.38 (0.35)	1.00 (0.74)
4d	0.11 (0.02)	0.15 (0.06)	0.29 (0.17)	0.33 (0.37)	0.99 (0.74)
4e	0.08 (0.01)	0.09 (0.02)	0.73 (0.15)	0.38 (0.35)	0.98 (0.75)
5a	0.08 (0.03)	0.22 (0.10)	0.39 (0.34)	0.91 (0.50)	1.00 (1.06)
5b	0.07 (0.02)	0.13 (0.05)	0.20 (0.37)	0.93 (0.51)	1.00 (1.03)
5c	0.07 (0.03)	0.14 (0.12)	0.48 (0.32)	0.89 (0.46)	0.95 (0.99)
5d	0.09 (0.03)	0.16 (0.07)	0.35 (0.36)	0.97 (0.45)	0.95 (1.04)
5e	0.08 (0.02)	0.09 (0.06)	0.22 (0.32)	0.65 (0.45)	0.99 (1.00)

Note.—The posterior means for Huelsenbeck’s *I* statistic are indicated in parentheses. When the correct root split is a modal root split, the corresponding marginal posterior probability appears underlined.

To evaluate the sensitivity of the analysis to the topological prior, the same analysis was performed using the structured uniform prior (supplementary table 1 and supplementary figs. 6 and 7, Supplementary Material online). This analysis gave very similar results, as we might expect given the similarity between the two priors.

##### NR2 Model

The NR and NR2 models differ only in the degree of structure used to model the perturbation from the underlying HKY85 rate matrix *Q^H^*. Therefore, as expected, the results from the analyses under the NR2 model are almost identical to those obtained under NR. Supplementary table 2, Supplementary Material online, summarizes the marginal posterior probabilities of the correct root split and the posterior means for Huelsenbeck’s *I* statistic for the analyses using the Yule prior (the posterior distributions of the root splits from a representative sample of simulations are shown in supplementary fig. 8, Supplementary Material online). Again, we see that as the degree of nonreversibility increases, the posterior becomes increasingly concentrated around the root split used to simulate the data. In terms of inference for the unrooted tree, as in the NR analyses, the true topology had posterior probability close to 1 in most cases (supplementary fig. 9, Supplementary Material online). The analysis of the same data sets performed with the structured uniform prior showed similar results (supplementary table 3 and supplementary figs. 10 and 11, Supplementary Material online).

#### Different Topologies and Branch Lengths

In a Bayesian analysis, the posterior distribution reflects information from both the prior and the data. When the prior and likelihood are comparably concentrated, but in conflict, the posterior can only represent a middle ground. In phylogenetics, inferences can be highly sensitive to the choice of prior for branch lengths and the topology itself (Yang et al. 2005; Alfaro and Holder 2006). Motivated by the kinds of conflicts that are likely to arise in the analysis of real biological data, we consider the robustness of posterior root inferences to conflicting prior and likelihood information concerning the rooted topology and branch lengths. In our analyses we adopt the commonly used Exp(10) prior for branch lengths and a Yule prior (or the approximating structured uniform prior) over rooted topologies. An Exp(10) prior for branch lengths asserts a strong prior belief that edges will be reasonably short. Therefore, given an unrooted topology that contains a long branch, the prior will typically support placement of the root midway along this branch to break it up into two shorter ones. In New Approaches section, we discussed properties of the Yule prior for rooted topologies on *n* taxa, in particular that for *n* odd (even) it induces an exact (near) uniform distribution over the subsets $T_{1:n-1},\ldots ,T_{k_{n}:n-k_{n}}$ of rooted trees with each root type. However, the subsets $T_{i:n-i}$ corresponding to unbalanced types, like 1:n−1, tend to contain many more trees than the subsets for more balanced types, like n/2:n/2 for *n* even or (n−1)/2:(n+1)/2 for *n* odd. The prior mass therefore has to be distributed among fewer trees in the latter case. It follows that the prior is not uniform over root splits and trees that are more balanced typically receive more prior mass than those that are unbalanced, as illustrated in supplementary figure 1, Supplementary Material online. In the remainder of this section, we therefore use simulation to examine posterior robustness in cases where prior–likelihood conflict arises due to a data-generating tree that is unbalanced or that contains a long branch.

**Fig. 1.**
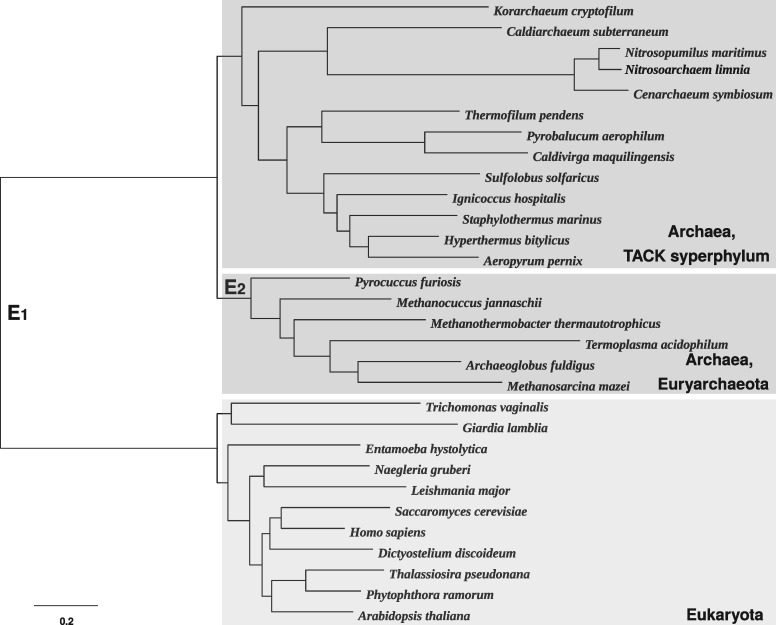
An unrooted 30-taxon tree derived from a recent analysis (Williams et al. 2012) describing the relationships between Archaea and Eukaryota. A root on the branch *E*_1_ corresponds to the three-domain hypothesis (located between monophyletic Archaea and Eukaryota), whereas a root on the branch *E*_2_ corresponds to the eocyte hypothesis (located within paraphyletic Archaea, separating Euryarchaeota from the clade comprising the TACK superphylum and Eukaryota).

We base our simulations on an unrooted 30-taxon tree derived from a recent analysis (fig. 1) (Williams et al. 2012). This tree describes the relationships between Archaea and Eukaryota. These relationships are still debated, concentrating on two competing hypotheses about the tree of life: 1) the three-domain hypothesis, according to which the root of the tree comprising Archaea and Eukaryota is placed on the branch separating monophyletic Archaea from monophyletic Eukaryota (branch *E*_1_) and 2) the eocyte hypothesis which places the root within a paraphyletic Archaea (branch *E*_2_). Based on this unrooted tree, we construct six different rooted trees by changing the placement of the root and the length of the branch *E*_1_ according to table 3.

**Table 3.** Six Rooted Trees for Simulating the Data.

Tree	Root Edge	Length of *E*_1_
1	*E*_1_	1.3
2	*E*_2_	1.3
3	*E*_1_	0.1
4	*E*_2_	0.1
5	*E*_1_	0.3
6	*E*_2_	0.3

Note.—The trees have the unrooted topology of the tree depicted in figure 1 but differ in the placement of the root and the length of the branch *E*_1_. Note that if a tree is rooted on branch *E_i_*, the root is placed at the middle of *E_i_*.

Trees 1, 3, and 5 are fairly balanced with root type 11:19, whereas Trees 2, 4, and 6 are more unbalanced with root type 6:24. The Yule prior assigns almost 30% more mass to the former rooted topology. In Trees 1 and 2 and, to a lesser extent, Trees 5 and 6, the unrooted topology contains a long internal branch. In Trees 3 and 4 this internal branch is short. Given the unrooted tree depicted in figure 1, the prior will therefore support placement of the root on branch *E*_1_ over *E*_2_, particularly if this branch is long.

We use the NR model to simulate a rate matrix *Q* with π=(0.25,0.25,0.25,0.25), *κ* = 2 and σ=0.3. In turn, this rate matrix is used to simulate three different alignments for each tree. These alignments are then analyzed under the NR model with the Yule prior.

**Fig. 2.**
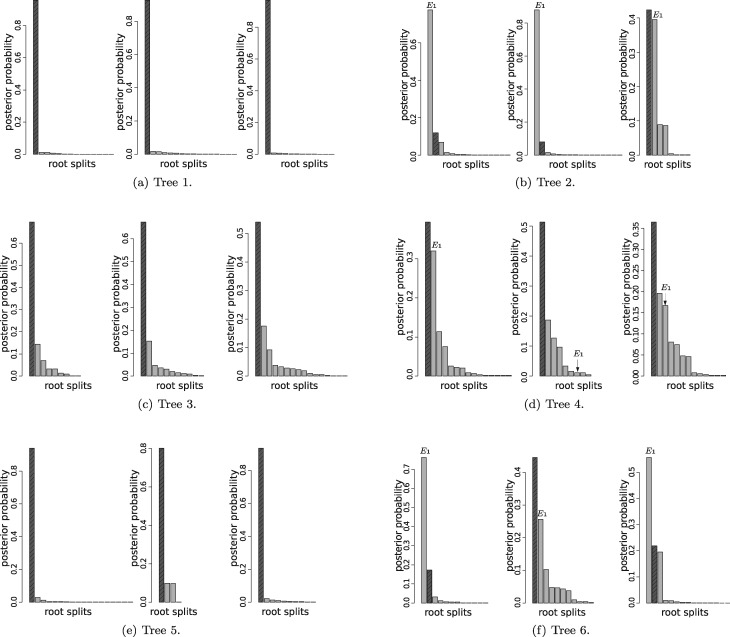
Posterior distribution of the root splits for three different alignments simulated for each of the six rooted trees according to table 3. Different bars on each plot represent different root splits ordered by posterior probabilities, with the highlighted bar representing the true root split. In the plots for Trees 2, 4, and 6, the split corresponding to a root on edge *E*_1_ is also marked.

*Tree 1:* Tree 1 is rooted on the long branch *E*_1_. Clearly the likelihood for data generated from this tree will support the correct placement of the root. Moreover, for the reasons expressed above, the prior will also support rooting on edge *E*_1_. It is not surprising, therefore, that we find the posterior is very concentrated around the true root position (fig. 2*a*).

*Tree 2:* In Tree 2, the root is placed on the much shorter branch *E*_2_, creating a fairly unbalanced unrooted topology with a long interior branch *E*_1_. As such, data generated under this tree will favor the correct root position on edge *E*_2_, but the prior will favor a root on branch *E*_1_. This creates prior–likelihood conflict. As expected, we find that the posterior probability of the true root drops substantially in comparison to the analysis for Tree 1 and in two of the three analyses, the posterior offers more support to a root on edge *E*_1_ (fig. 2*b*).

*Tree 3*: Tree 3 has the same rooted topology as Tree 1, but the root branch *E*_1_ is now much shorter and the unrooted topology does not contain any long edges. As for Tree 1, prior–likelihood conflict does not arise but there is no longer such pronounced prior support for placement of the root on edge *E*_1_. Nevertheless, we find that the posterior is still concentrated around the true root position (fig. 2*c*).

*Tree 4*: Tree 4 has the same rooted topology as Tree 2, but the long interior branch *E*_1_ is now shortened to 0.1. Although the Yule prior generally favors more balanced trees than Tree 4, the prior for branch lengths no longer offers overwhelming support to placement of the root on edge *E*_1_. We find that the true root can now be recovered as the posterior mode (fig. 2*d*) but with less support than in the analysis for Tree 3.

*Tree 5*: Tree 5 has the same rooted topology as Trees 1 and 3, but the root edge *E*_1_ has length 0.3, which lies between the corresponding values for Trees 1 and 3. As expected, we find that the true root is inferred as the posterior mode (fig. 2*e*), and the posterior is less (more) concentrated around the mode in comparison to the analysis of Tree 1 (Tree 3).

*Tree 6*: Tree 6 has the same rooted topology as Trees 2 and 4, but the internal edge *E*_1_ has length 0.3, which lies between the corresponding values for Trees 2 and 4. The unrooted topology has a moderately long interior edge and the rooted topology is unbalanced, leading to some prior–likelihood conflict. We find that a root on edge *E*_1_ sometimes receives more posterior support than the true root (fig. 2*f*), although, as expected, this effect is less pronounced than in the analysis for Tree 2.

This simulation experiment illustrates the sensitivity of root inferences to conflict between the prior and the likelihood. The effect of a mismatch in information about branch lengths is particularly noticeable. Given a particular unrooted topology, although the likelihood might support the presence of a long branch in the corresponding rooted tree, an Exp(10) prior does not, and therefore favors placement of the root on the long edge. Ideally constructing a more flexible prior that more explicitly models topology and branch lengths jointly will contribute to better root inference. However, given the absence of very long branches, our results show that the model is still able to extract information from the data about the root even in the face of prior–likelihood conflict.

#### Different Levels of Nonstationarity in the Evolutionary Process

If it was reasonable to assume that the evolutionary process for a particular alignment was stationary, we would expect the empirical sequence composition for each species to be approximately the same. However, this is often not the case in experimental data (Foster 2004; Cox et al. 2008). The NR and NR2 models assume that the evolutionary process is stationary. Therefore, in cases where this is not a reasonable assumption, model misspecification may have an effect on our posterior inferences. For example, it has been previously shown that failure to account for compositional heterogeneity can lead to inferring incorrect topologies with strong support (Foster 2004; Cox et al. 2008; Foster et al. 2009; Williams et al. 2012). The remainder of this section describes a simulation experiment to examine the robustness of posterior root inference to situations where the data-generating process exhibits different levels of nonstationarity.

We base our simulations on Tree 3, described previously, as it has a rooted topology and branch lengths which are consistent with the prior, removing prior–likelihood conflict about the tree as a potential source of confounding. In these experiments, we simulated data using a variant of the NR model in which the distribution at the root of the tree was equal to $\pi_{\text{root}}$, where $\pi_{\text{root}}$ was not equal to the stationary distribution $\pi_{Q}$ associated with the NR rate matrix *Q*. We used the same rate matrix *Q* as that employed in the simulations for Tree 3 in the previous subsection to allow comparison with the stationary case. We chose two values for $\pi_{\text{root}}$ at Euclidean distances of 0.2 and 0.4 along the line connecting $\pi_{Q}$ = (0.246, 0.287, 0.189, 0.278) to the extreme composition vector (0.0, 0.5, 0.0, 0.5), which preserves the ordering of the nucleotides in $\pi_{Q}$. These were $\pi_{\text{root},M}$ = (0.1968, 0.3296, 0.1512, 0.3224) and $\pi_{\text{root},L}$ = (0.1476, 0.3732, 0.1134, 0.3668), respectively. These root distributions are intended to represent increasing degrees of nonstationarity and model misspecification, with $\pi_{\text{root},L}$ constituting a very biased composition vector. For both root compositions, we simulated five alignments and analyzed each of them under the NR model with the Yule prior.

Table 4 summarizes the marginal posterior probabilities of the correct root split for the various simulations (the posterior distributions of the root splits are shown in supplementary fig. 12, Supplementary Material online). Indicated in parentheses are the metric variances (Pawlowsky-Glahn and Egozcue 2001) across taxa of the sequence composition for each simulated alignment. This is a global measure of spread for compositional data, with larger values indicating a greater degree of compositional heterogeneity. When the degree of nonstationarity is modest, it seems that the true root is still recovered as the posterior mode, although the posterior is more diffuse than it was in the stationary case. When the level of nonstationarity is larger, as expected, posterior support for the true root is slightly eroded due to the greater degree of model misspecification. The posterior distributions for the unrooted topology, displayed in supplementary figure 13, Supplementary Material online, show little evidence of an effect of the degree of nonstationarity, with the true root recovered as the posterior mode in most cases. Therefore, it seems that the model offers robustness to, at least, moderate degrees of nonstationarity, providing reassurance of the contribution of the model to questions of root position in analyses of real biological data.

**Table 4.** Marginal Posterior Probabilities of the Correct Root Split for the Simulations under the Nonstationary Variant of the NR Model, Analyzed Using the NR Model, with Stationary Distribution $\pi_{Q}$, and the Yule Prior.

Data Set	$\pi_{Q}$	$\pi_{\text{root},M}$	$\pi_{\text{root},L}$
1	0.69 (0.00029)	0.39 (0.00041)	0.37 (0.00087)
2	0.65 (0.00034)	0.64 (0.00043)	0.44 (0.00061)
3	0.56 (0.00039)	0.34 (0.00037)	0.18 (0.00100)
4	0.41 (0.00028)	0.60 (0.00040)	0.31 (0.00067)
5	0.47 (0.00041)	0.51 (0.00052)	0.31 (0.00062)

Note.—The distributions at the root are $\pi_{Q}$ and two distributions, $\pi_{\text{root},M}$ and $\pi_{\text{root},L}$, increasingly displaced from $\pi_{Q}$. When the correct root split is a modal root split, the corresponding marginal posterior probability appears in italics. The metric variance of the empirical compositions in the simulated alignments is indicated in parentheses.

### Analysis of Experimental Data

#### Rooting the Radiation of Paleopolyploid Yeasts

**Fig. 3.**
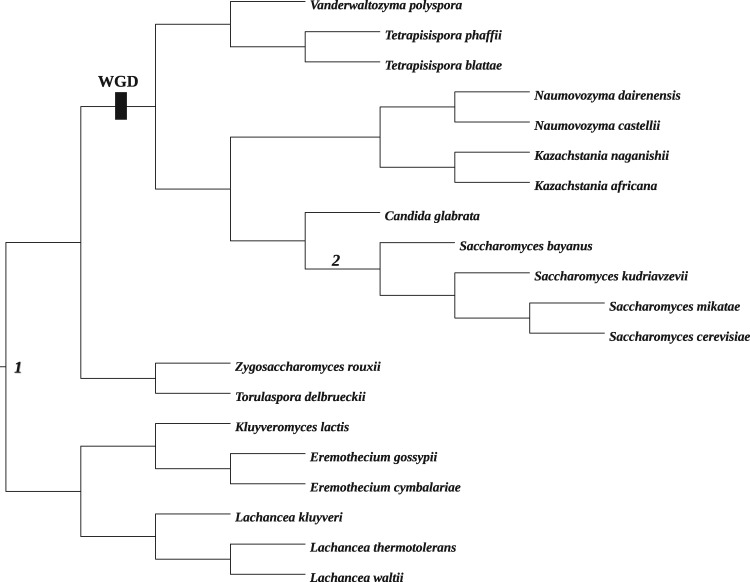
Rooted phylogeny of the paleopolyploid yeasts supported by the whole-gene duplication analysis (not drawn to scale), reproduced from the YGOB web site (Byrne and Wolfe 2005; http://ygob.ucd.ie 2015; last accessed January 1, 2015). The tree is rooted according to the outgroup method based on an analysis with the GTR + I+G model in a maximum likelihood framework (Hedtke et al. 2006). Roots 1 and 2 represent the two most plausible posterior root splits in the current analysis.

We next investigated the performance of the NR and NR2 models on a real biological data set for which there is broad biological consensus on the root position (Byrne and Wolfe 2005; Hedtke et al. 2006). The lineage leading to *Saccharomyces cerevisiae* (brewer’s yeast) and its relatives underwent a conserved whole-genome duplication (WGD) about 100 million years ago (Wolfe and Shields 1997; Kellis et al. 2004). Evidence for this WGD, in the form of duplicated genes and genomic regions, is shared by all post-WGD yeasts and defines the group as a clade from which the root of the *Saccharomycetales* is excluded (fig. 3) (Byrne and Wolfe 2005).

**Fig. 4.**
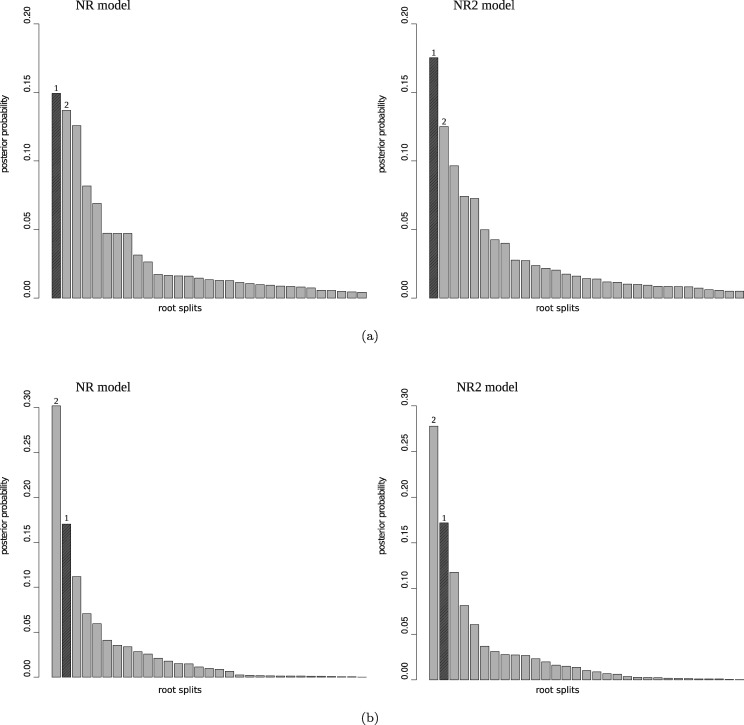
The posterior distribution of the root splits of the paleopolyploid yeasts data set for both NR and NR2 models analyzed (*a*) with the structured uniform prior and (*b*) with the Yule prior. Different bars on the plot represent different root splits on the posterior distribution of trees ordered by posterior probabilities (roots 1 and 2 are mapped in fig. 3). In (*a*), the analysis performed with the structured uniform prior, the root split supported by outgroup rooting (Hedtke et al. 2006) has the highest posterior probability (root 1, highlighted), whereas root 2 is placed within the post-WGD clade. In (*b*), the analysis performed with the Yule prior, the root split supported by outgroup rooting (Hedtke et al. 2006) has the second highest posterior probability (root 1, highlighted). The posterior modal root 2 is placed within the post-WGD clade.

The root inferred through outgroup analysis separates a clade comprising *Eremothecium gossypii*, *Eremothecium cymbalariae*, *Kluyveromyces lactis*, *Lachancea kluyveri*, *Lachancea thermotolerans*, and *Lachancea waltii* from the other species (Hedtke et al. 2006). We analyzed an alignment of concatenated large and small subunit ribosomal DNA sequences for 20 yeast species, with a combined length of 4,460 bp. The sequences were aligned with MUSCLE (Edgar 2004), and poorly aligned regions were detected and removed using TrimAl (Capella-Gutiérrez et al. 2009). The alignment is available in the Supplementary Material online. We analyzed this data set with the NR and NR2 models, using both the Yule prior and the structured uniform prior. In the analysis with the structured uniform prior, the root split supported by outgroup rooting (Hedtke et al. 2006) has the highest posterior probability (root 1 in fig. 3) for both models. However, there is a substantial amount of uncertainty represented by the nonnegligible posterior probabilities of the other root splits (fig. 4*a*) and, for example, the second most plausible root is located within the post-WGD clade (root 2 in fig. 3). This posterior uncertainty is also reflected in the sensitivity of the analysis to the topological prior: Although the structured uniform prior recovered the root supported by the outgroup analysis with the highest posterior support, the Yule prior instead recovered this root with the second-highest support (fig. 4*b*). The most plausible root inferred with the Yule prior is placed within the post-WGD clade (root 2 in fig. 3) contradicting the WGD analysis.

The posterior for Huelsenbeck’s *I* statistic is suggestive of a nonnegligible degree of nonreversibility in the data (the posterior mean is 0.2 for the analysis with the NR model, 0.14 for the analysis with the NR2 model). In our simulations, larger values of *I* were generally required to infer the true root with high posterior probability. However, the support offered to the widely accepted outgroup root in this analysis shows that it is possible to extract useful root information in spite of the data suggesting only a modest degree of nonreversibility.

**Fig. 5.**
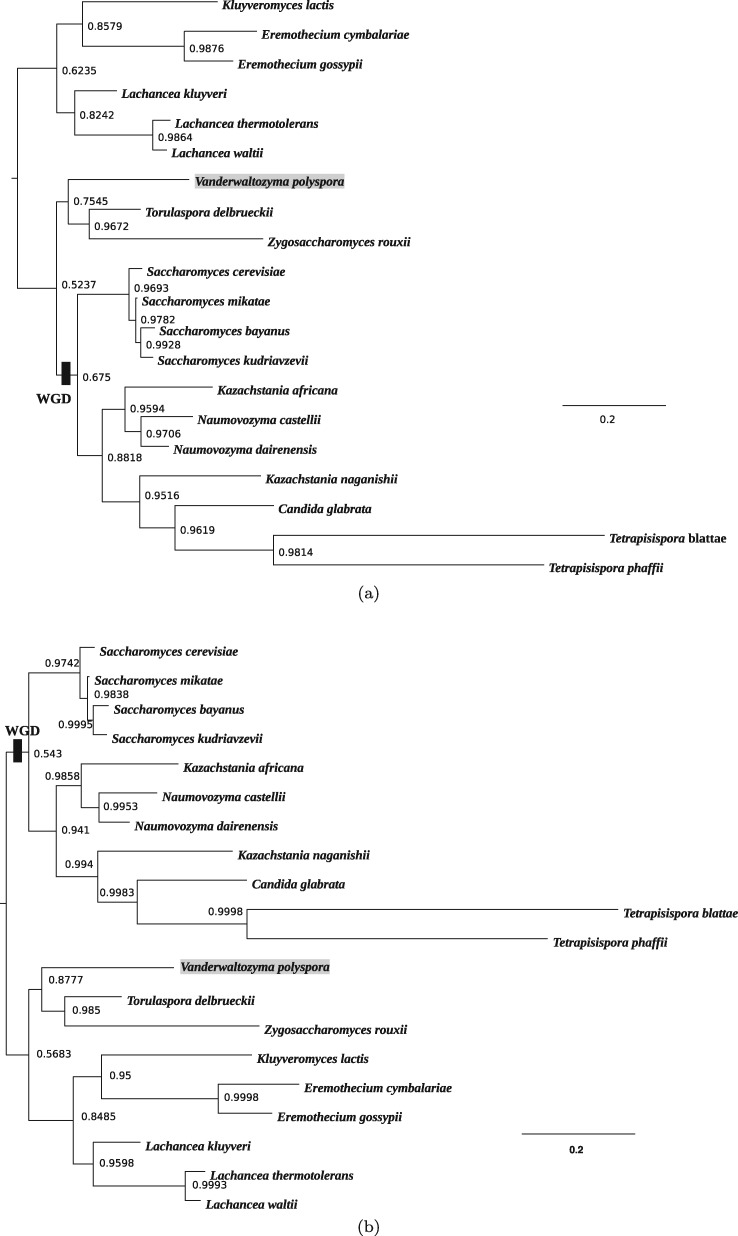
Rooted majority rule consensus tree of the paleopolyploid yeasts data set, inferred under the NR model using (*a*) the structured uniform prior and (*b*) the Yule prior, with the WGD event mapped. The analysis is based on the alignment of concatenated large and small subunit ribosomal DNA sequences for 20 yeast species, 4,460 bp. The trees differ from that supported by the WGD analysis by the placement of *Vanderwaltozyma polyspora* (highlighted) within the pre-WGD clade. The consensus trees obtained under the analyses using the NR2 model are very similar and so not shown.

The unrooted topologies of the rooted majority rule consensus trees from the analyses with the two topological priors (fig. 5) differ from that supported by the WGD analysis by the placement of *Vanderwaltozyma polyspora*. Although the WGD analysis places it within the post-WGD clade, in our analysis this taxon is located within the pre-WGD clade. This result is consistent with our posterior inferences from fitting the HKY85 and GTR models. Interestingly, it is also consistent with the analysis performed with the site-heterogeneous CAT-GTR model (Lartillot and Philippe 2004) where *V. polyspora* is, again, excluded from the post-WGD clade (not shown). The placement of *V. polyspora* outside the WGD clade is surprising given that the genome of *V. polyspora* preserves evidence of having undergone WGD (Scannell et al. 2007). Although this result requires further investigation, the similarity between the consensus trees obtained with the CAT-GTR model and our nonreversible models suggests that the nonreversible models can not only extract meaningful information about the root position, but also capture information for inferring the unrooted topology. However, the minor mismatch of the topologies inferred in our analysis with that supported by WGD and outgroup analyses (Hedtke et al. 2006) confirms the presence of some features of the data that our models do not account for. For example, ribosomal RNA function depends on the molecule folding into a complex three-dimensional shape. Interactions among sites that are distant in the primary sequence, but close in the three-dimensional structure, are likely to induce site-specific selective constraints that are not accounted for in our models. Thus further refinement of the models, for instance, allowing compositional heterogeneity across sites, might be necessary to improve the ability of the models to provide better insight into the evolution of paleopolyploid yeasts.

It is worth noting that the root split on the majority rule consensus tree (fig. 5*b*) does not match the marginal posterior modal root split (fig. 4*b*). This happens because the consensus tree is a conditional summary, computed recursively from the leaves to the root, which depends upon the plausibility of subclades. On the other hand, the posterior over root split is a marginal summary that averages over the relationships expressed elsewhere in the tree; see Appendix B for an illustrative example.

### Analysis of the Ribosomal Tree of Life

We have also applied the models to a data set for which there is still debate about the unrooted topology and root position: the ribosomal tree of life. Recall that the debates are centered on two hypotheses. According to the three-domain hypothesis, Archaea is monophyletic, sharing a common ancestor with Eukaryota (Woese et al. 1990). The other hypothesis, called the eocyte hypothesis, suggests that Archaea is paraphyletic and Eukaryota originated from within Archaea (Lake 1988; Rivera and Lake 1992; Cox et al. 2008). Recent analyses of ribosomal RNA data have demonstrated that topological inferences can be sensitive to the choice of substitution model. When homogeneous models are used for the analysis they often recover the three-domain tree, whereas heterogeneous models generally recover the eocyte tree (Cox et al. 2008; Williams et al. 2012). In addition, there is also external evidence for the eocyte hypothesis. For example, newly discovered archaeal species whose genomes encode many eukaryote-specific features provide additional support for the eocyte hypothesis (Spang et al. 2015).

**Fig. 6.**
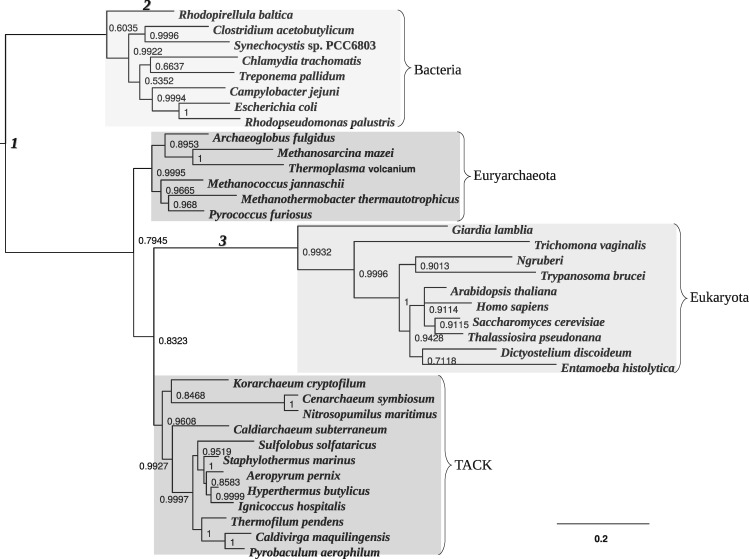
Rooted majority rule consensus tree for the tree of life data set, inferred under the NR model using the Yule prior. The tree supports the eocyte hypothesis by placing Eukaryota within Archaea, as a sister group to the TACK superphylum. Roots 1, 2, and 3 are the root splits having the highest posterior support in the current analysis. Posterior support for these root splits is shown in figure 7. The consensus tree inferred under the NR2 model using the Yule prior is similar and so not shown. The same is true for both models using the structured uniform prior.

**Fig. 7.**
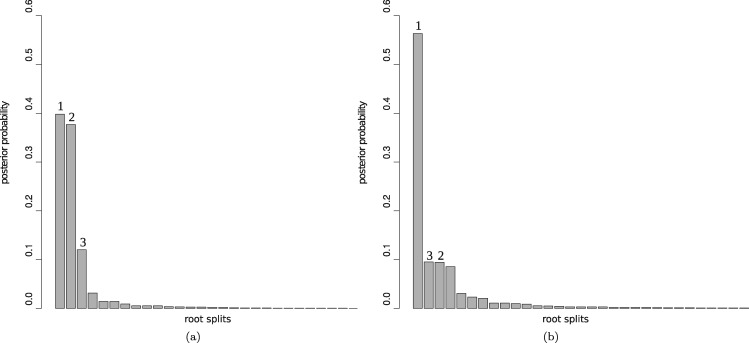
The posterior distribution of the root splits of the tree of life data set for the NR model analyzed with (*a*) the Yule prior and (*b*) with the structured uniform prior. Different bars on the plot represent different root splits on the posterior distribution of trees (ordered by posterior probabilities). The root split on the branch leading to Bacteria has the highest posterior probability (root 1). Root 2 is placed within Bacteria (on the branch leading to *Rhodopirellula baltica*) and root 3 is placed on the branch leading to Eukaryota (the roots are mapped in fig. 6). The posterior distributions obtained under the analyses using the NR2 model are very similar and so not shown.

Here we analyzed aligned concatenated large and small subunit ribosomal RNA sequences from archaeal, bacterial, and eukaryotic species (36 taxa, 1,734 sequence positions), including the recently discovered archaeal groups: Thaumarchaeota, Aigarchaeota, and Korarchaeota. These new groups are closely related to Crenarchaeota and together they form the so-called TACK superphylum (Guy and Ettema 2011; Kelly et al. 2011; Williams et al. 2012; Lasek-Nesselquist and Gogarten 2013). The alignment is available in the Supplementary Material online. Previous analysis of this data set performed with the CAT-GTR model recovered an eocyte topology (Williams et al. 2012). Fitting the simpler HKY85 and GTR models also support this hypothesis. However, these analyses were not able to infer the root because they used only reversible rate matrices in stationary substitution models. We analyzed these data with both the NR and NR2 models using both the Yule prior and the structured uniform prior. In all cases we recovered the eocyte topology with similar posterior support (fig. 6). The analysis with the Yule prior assigned high posterior support to two roots splits (fig. 7*a*)—one on the branch leading to Bacteria (root 1 in fig. 6), the other within Bacteria, on the branch leading to *Rhodopirellula baltica* (root 2 in fig. 6). This inference is in accord with current biological opinion about the root of the tree of life, which places the root either on the branch leading to Bacteria or within Bacteria (Baldauf et al. 1996; Hashimoto and Hasegawa 1996; Cavalier-Smith 2006; Skophammer et al. 2007). However, in the analysis performed with the structured uniform prior, the support for the root within Bacteria decreased and that for the root on the bacterial branch increased (fig. 7*b*). This analysis illustrates the sensitivity of the inference to the choice of topological prior and confirms the importance of the choice of prior in Bayesian phylogenetics. The posterior mean of the Huelsenbeck’s *I* statistic is 0.18 for the analysis with the NR model and 0.17 for the analysis with the NR2 model. Again, this is suggestive of a moderate degree of nonreversibility in the data. Therefore, modeling other features of the data that also provide root information could make a valuable contribution to the inference.

## Discussion

We presented two hierarchical nonreversible models for inferring rooted phylogenetic trees. The biologically dubious assumption of reversibility which underpins standard models of sequence evolution is relaxed by applying a stochastic perturbation to the rate matrix of a reversible model. This perturbation makes the likelihood dependent on the position of the root, enabling us to infer the root directly from the sequence alignment. In the first model (the NR model), we use only one variation component and perform a log-normal perturbation on the space of all possible rate matrices. In contrast, the second model (the NR2 model) utilizes two variation components and the perturbation is performed on the space of reversible and nonreversible rate matrices separately. This separation allows us to judge the extent of the different types of perturbation.

The results on the simulated data with different levels of nonreversibility show that the correct root can be recovered with greater posterior support when the degree of nonreversibility in the data-generating model is larger. We also investigated the robustness of posterior root inferences to situations where information from the prior and data are in conflict. Given a particular unrooted topology, our Yule prior for rooted trees and Exp(10) prior for branch lengths offer most support to balanced trees with short edges. Our simulations show that we can still recover the true root in the posterior when the data-generating tree is unbalanced or the associated unrooted topology contains a long edge. However, when this edge is very long, it can mislead the root inference. Similarly, we investigated the robustness of posterior inferences to situations where the data-generating process is nonstationary, meaning the (stationary) NR model is misspecified. We found that our root inferences are robust against modest degrees of nonstationarity, but that when the degree of nonstationarity increases, the posterior over root splits becomes more diffuse, typically offering less support to the true root.

We applied our models to two biological data sets. These analyses agree with our simulations in suggesting that our nonreversible models can recover useful rooting information, this time from real biological sequence alignments. The analyses of both the yeast and tree of life data sets recover the widely agreed root. However, both data sets show some prior sensitivity, even though the two topological priors (the Yule prior and the structured uniform prior) share similar features. To investigate this issue we computed a log Bayes factor (Kass and Raftery 1995) to compare the Yule prior (*Y*) with the structured uniform prior (*S*) for both examples with real data; see Materials and Methods for computational details. Although usually used to compare models, the Bayes factor really compares prior–likelihood combinations and so can also be used to assess which of the two priors is most consistent with the data. The log Bayes factor for the yeasts data set is $\text{log}\;B_{YS}$ = 2.27 suggesting that there is evidence against the structured uniform prior, though the evidence is not strong. The log Bayes factor for the tree of life data set is $\text{log}\;B_{YS}$ = 0.12 suggesting that there is no difference between the priors. Therefore, the more noticeable prior sensitivity in the analysis of the yeasts data set is likely to be due to the greater difference in consistency between the data and each of the two priors.

Although Huelsenbeck’s *I* statistic provides evidence of a nonnegligible degree of nonreversibility in both biological data sets, the analyses display high levels of posterior uncertainty. This suggests that the information about the root may be obscured by other signals that are not accounted for by our current models. For instance, our models assume the evolutionary process is stationary but this assumption is clearly violated for the tree of life data set, where the metric variance of the sequence compositions was 0.0096, with empirical GC content ranging from 41% for *Entamoeba histolytica* to 69% for *Giardia lamblia*. Our simulation experiment suggested that a metric variance of this size is large enough for model misspecification to affect root inference. The models may therefore benefit from further development, for example, to model the nonstationarity of the process. Another extension which we are currently exploring is to develop similar models for protein data, applying a log-normal perturbation to the rate matrix of an empirical model of amino acid substitution, such as WAG (Whelan and Goldman 2001) or LG (Le and Gascuel 2008). Unfortunately, the general perturbed rate matrix *Q* for a 20-character alphabet contains 380 off-diagonal elements, compared with only 12 for the 4-character DNA alphabet. Although computational challenges associated with sampling all of the additional parameters have so far frustrated efforts to produce a viable MCMC algorithm, future work will investigate more efficient computational schemes for inference. Nonetheless, our findings illustrate that, as they stand, our nonreversible models NR and NR2 can be useful for simultaneously inferring the unrooted topology and root position from modestly sized biological data sets.

## Materials and Methods

We work within the Bayesian paradigm and base our inferences on the posterior distribution of the unknowns in the model. According to Bayes theorem, the posterior distribution is proportional to the prior times the likelihood. For the NR model, for example, the posterior distribution factorizes as

(3) π(π,κ,σ,Q,α,ℓ,τ|Data)∝π(Q|π,κ,σ)×π(π,κ,σ,α,ℓ,τ)×π(Data|Q,α,ℓ,τ).

This distribution is analytically intractable and so we build up a numerical approximation by sampling from it using MCMC methods, specifically a Metropolis-within-Gibbs sampling scheme. In the remainder of this section, we first describe the calculation of the likelihood function, before outlining details of the MCMC algorithm. Finally, we provide practical details of the application of this algorithm to the analyses presented in Results section.

### Likelihood

The likelihood function summarizes the information available from the data about the unknowns in the model including the phylogenetic tree. Since we assume that alignment sites evolve independently of each other, the likelihood can be expressed as a product of the likelihoods of the *n* individual sites of the alignment. If we denote θ to be the parameters of the substitution process, the likelihood takes the form

$$\pi (\text{Data}|\theta ,\alpha ,\ell ,\tau )=\prod_{i=1}^{n}\pi (D_{i}|\theta ,\alpha ,\ell ,\tau ),$$

where *D_i_* is the column of nucleotides at site *i*. The probability of the data at a site *i* is given by

$$\pi (D_{i}|\theta ,\alpha ,\ell ,\tau )=\sum_{X}\pi_{X(\text{root})}\prod_{\text{edges}\ell =(v,w)}p_{X(v),X(w)}(\ell ),$$

where *v* and *w* are the vertices at the two ends of edge *ℓ* and *X*(*u*) denotes the nucleotide at a vertex *u*. The sum is taken over all functions *X* from the vertices to Ω such that *X*(*u*) matches data $D_{i}(u)$ for all leaf vertices *u*. We assume a stationary model and so take the probability at the root $\pi_{X(\text{root})}$ to be $\pi_{Q,X(\text{root})}$, which comes from $\pi_{Q}$, the theoretical stationary distribution associated with *Q* (note that this is not the same as π, the stationary distribution of the underlying HKY85 model).

### MCMC Algorithm

#### NR Model

For the NR model, the posterior distribution for the unknowns was summarized through equation (3). At each iteration of the MCMC algorithm, the following steps are performed:

1. update the parameters of the substitution model (π,κ,σ,Q,α);
2. update the branch lengths ℓ and the rooted topology *τ*.

In step (1) we update the parameters using a Dirichlet random walk proposal for π and log-normal random walk proposals for the other parameters. Move (2) consists of a series of Metropolis–Hastings steps to update each branch length one at a time using a log-normal random walk proposal and then updating the rooted topology and branch lengths (in a joint move) through three types of proposal: nearest-neighbor interchange, sub-tree prune and regraft, and a proposal that moves the root; see the work of Heaps et al. (2014) for complete details of all three moves.

#### NR2 Model

Here, the posterior distribution of the unknowns takes the form

$$\pi (\pi ,\kappa ,\sigma_{R},\sigma_{N},\varepsilon ,\eta ,\alpha ,\ell ,\tau |\text{Data})\propto \pi (\pi ,\kappa ,\sigma_{R},\sigma_{N},\epsilon ,\eta ,\alpha ,\ell ,\tau )\times \pi (\text{Data}|\pi ,\kappa ,\epsilon ,\eta ,\alpha ,\ell ,\tau )$$

and an analogous Metropolis-within-Gibbs algorithm is used to generate posterior samples.

### MCMC Implementation

In Results section, all results were based on (almost) un-autocorrelated posterior samples of size 5K. These samples were obtained by running the MCMC algorithm for at least 1,000K iterations, discarding at least 500K iterations as burn-in and then thinning by taking every 100th iterate to remove autocorrelation. Convergence was diagnosed using the procedure described in the study of Heaps et al. (2014). This involved initializing two MCMC chains at different starting points and graphically comparing the chains through properties based on model parameters and the relative frequencies of sampled clades. In all cases, the graphical diagnostics gave no evidence of any lack of convergence. The MCMC inferential procedures are programmed in Java and a software implementation can be found in the Supplementary Material online. For alignments on 30 taxa with 2,000 sites, such as those used in the simulation experiments, generating 1,000K iterations took approximately 11 days on a High-Performance Computing cluster where each server has two 12-core Xeon E5-2680 v3 CPUs and 128GB DDR3 1600 RAM.

### Bayes Factor

In the analyses of both the yeast and tree of life data sets, we observed some posterior sensitivity to the choice of topological prior. We therefore computed a log Bayes factor to assess which of the two priors was most consistent with the data. In general, numerical evaluation of the Bayes factor is a very challenging integration problem. In this case, however, we are simply comparing two prior–likelihood combinations in which only the topological priors differ. As a result, the calculation can be simplified substantially, allowing the Bayes factor to be computed as a product of the ratios of the topological priors and the topological posteriors, evaluated at any tree with nonzero support. This effectively uses a simplified form of Chib’s method (Chib 1995; Chib and Jeliazkov 2001) for the approximation of marginal likelihoods. Full details of the calculation can be found in the Supplementary Material online.

## Appendix A

The two-stage perturbation relies upon the underlying geometry of the space of Markov rate matrices and is achieved in the following way. We work on a log scale element-wise with all matrices, ignoring diagonal elements. The set of all possible 4 × 4 rate matrices *M* is therefore identified with ${\mathbb{R}}^{12}$, which we equip with the standard inner product. The set *M*_HKY_ of HKY85 matrices and the set *M*_GTR_ of GTR matrices form nested subsets of *M*. The perturbations are best understood by first supposing that *M*_HKY_ and *M*_GTR_ are linear subspaces of $M={\mathbb{R}}^{12}$. In the first stage, a matrix $Q^{H}\in M_{\text{HKY}}$ is randomly perturbed to a matrix $Q^{R}\in M_{\text{GTR}}$. The perturbation is performed orthogonal to *M*_HKY_ so that *Q^H^* is the closest element of *M*_HKY_ to *Q^R^* with respect to the Euclidean distance on *M*. In other words, the orthogonality condition ensures that *Q^H^* is the geometrical center of the distribution for *Q^R^*. The second stage randomly perturbs *Q^R^* to a general matrix Q∈M and is orthogonal to *M*_GTR,_ so in a similar way, *Q^H^* and *Q^R^* are the closest elements of *M*_HKY_ and *M*_GTR_ to *Q*, respectively.

However, *M*_HKY_ and *M*_GTR_ are not linear subspaces of *M*, but instead are curved submanifolds. To achieve an analogous perturbation procedure to the description above, we approximate by working on the tangent spaces to *M*_HYK_ and *M*_GTR_ at each point $Q^{H}\in M_{\text{HKY}}$ and perturb orthogonal to these. The result is that, for example, *Q^H^* is locally the closest element of *M*_HKY_ to *Q^R^*. Again, the orthogonality assumptions are imposed so that *Q^H^* lies at the center of the distribution on *M*, at least approximately. To perform the perturbations we therefore need to compute tangent vectors at *Q^H^*. Recall that working element-wise on a log scale, the off-diagonal elements of the rate matrix of the NR model can be expressed as, for i≠j

(4) $$\text{log}\;q_{ij}=\;\text{log}\;q_{ij}^{H}+\epsilon_{ij},$$

where the $\epsilon_{ij}$ are independent $N(0,\sigma^{2})$ quantities. The element-wise log of the HKY85 matrix *Q^H^* in equation (4) is

(5) $$\begin{array}{cc} \;\text{log}\;q_{ij}^{H} & =\tilde{\kappa }(e_{1}e_{2}^{T}+e_{2}e_{1}^{T}+e_{3}e_{4}^{T}+e_{4}e_{3}^{T})+\sum_{i=1}^{4}{\tilde{\pi }}_{i}se_{i}^{T}\\ & =\tilde{\kappa }(e_{1}e_{2}^{T}+e_{2}e_{1}^{T}+e_{3}e_{4}^{T}+e_{4}e_{3}^{T})+\sum_{i=1}^{3}{\tilde{\pi }}_{i}se_{i}^{T}\\ & +\;\text{log}\;\left(1-e^{{\tilde{\pi }}_{1}}-e^{{\tilde{\pi }}_{2}}-e^{{\tilde{\pi }}_{3}}\right)se_{4}^{T} \end{array},$$

where $\left({\tilde{\pi }}_{1},{\tilde{\pi }}_{2},{\tilde{\pi }}_{3},{\tilde{\pi }}_{4}\right)$=$(\text{log}\;\pi_{A},\;\text{log}\;\pi_{G},\;\text{log}\;\pi_{C},\;\text{log}\;\pi_{T}),\tilde{\kappa }=\;\text{log}\;\kappa ,\;e_{i}$ is the *i*th standard basis vector of ${\mathbb{R}}^{4}$ and $s={\left(1,1,1,1\right)}^{T}$. By differentiating equation (5) with respect to the parameters ${\tilde{\pi }}_{1},{\tilde{\pi }}_{2},{\tilde{\pi }}_{3}$ and $\tilde{\kappa }$ we obtain four linearly independent vectors in *M* that are locally tangent to *M*_HKY_ at *Q^H^*, and we denote these $V_{1},V_{2},V_{3},V_{4}$. These tangent vectors correspond to the 4 × 4 matrices

$$V_{i}=se_{i}^{T}-\;\text{exp}\;({\tilde{\pi }}_{i}-{\tilde{\pi }}_{4})se_{4}^{T}\text{ for}\;i=1,2,3,$$

and

$$V_{4}=e_{1}e_{2}^{T}+e_{2}e_{1}^{T}+e_{3}e_{4}^{T}+e_{4}e_{3}^{T}.$$

The element-wise log of the general GTR matrix is

$$\sum_{i=1}^{4}{\tilde{\pi }}_{i}se_{i}^{T}+\sum_{i<j}\tilde{\rho_{ij}}\left(e_{i}e_{j}^{T}+e_{j}e_{i}^{T}\right),$$

where ${\tilde{\rho }}_{ij}$ is the log of the exchangeability parameter *ρ_ij_*. By considering the derivatives with respect to the ${\tilde{\rho }}_{ij}$ parameters, it can be seen that the following vectors lie in the tangent space to *M*_GTR_ at *Q^H^*:

$$\begin{array}{c} V_{5}=\left(e_{1}e_{2}^{T}+e_{2}e_{1}^{T}\right)-\left(e_{3}e_{4}^{T}+e_{4}e_{3}^{T}\right),\\ V_{6}=\left(e_{1}e_{3}^{T}+e_{3}e_{1}^{T}\right)+\left(e_{2}e_{4}^{T}+e_{4}e_{2}^{T}\right),\\ V_{7}=\left(e_{1}e_{3}^{T}+e_{3}e_{1}^{T}\right)-\left(e_{2}e_{4}^{T}+e_{4}e_{2}^{T}\right),\\ V_{8}=\left(e_{1}e_{4}^{T}+e_{4}e_{1}^{T}\right)+\left(e_{2}e_{3}^{T}+e_{3}e_{2}^{T}\right),\\ V_{9}=\left(e_{1}e_{4}^{T}+e_{4}e_{1}^{T}\right)-\left(e_{2}e_{3}^{T}+e_{3}e_{2}^{T}\right). \end{array}$$

The vectors $V_{1},V_{2},\ldots ,V_{9}$ are linearly independent by construction. Standard linear algebra can be used to extend this to a basis $V_{1},\ldots ,V_{12}$ of ${\mathbb{R}}^{12}$.

Next, the QR factorization algorithm is applied to the 12 × 12 matrix with columns $V_{1},\ldots ,V_{12}$ to obtain an orthonormal basis of tangent vectors $W_{1},\ldots ,W_{12}$ that is used to perturb *Q^H^*. First, *Q^H^* is perturbed using $\nu_{1},\ldots ,\nu_{5}$ to obtain a GTR matrix *Q^R^* where, for i≠j

$$\text{log}\;q_{ij}^{R}=\;\text{log}\;q_{ij}^{H}+\sum_{k=5}^{9}\nu_{k-4}W_{kij},$$

and the *ν_k_* are independent $N(0,\sigma_{R}^{2})$ and *W_kij_* is the (*i*, *j*)th element of the 4 × 4 matrix corresponding to *W_k_*. The choice of basis $W_{1},\ldots ,W_{12}$ ensures that this perturbation is locally orthogonal to *M*_HKY,_ and that the perturbation is otherwise isotropic within the subset of GTR matrices. The second stage perturbs *Q^R^* into the space of nonreversible rate matrices using $\eta_{1},\eta_{2},\eta_{3}$: for i≠j

$$\text{log}\;q_{ij}=\;\text{log}\;q_{ij}^{R}+\sum_{k=10}^{12}\eta_{k-9}W_{kij},$$

and the *η_k_* are independent $N(0,\sigma_{N}^{2})$ quantities. This perturbation is locally perpendicular to *M*_GTR._ The equation determines the off-diagonal elements of the nonreversible rate matrix *Q*, whereas the diagonal elements are fixed to make the row sums zero. The size of the perturbation variance $\sigma_{R}^{2}$ can be thought of as representing the extent to which the rate matrix *Q* departs from the class of HKY85 models remaining within the class of reversible models, whereas $\sigma_{N}^{2}$ represents the extent to which *Q* departs from being reversible.

## Appendix B

The root on the majority rule consensus tree and the mode of the posterior distribution for root splits are different point summaries of the posterior distribution for root positions. Both can be approximated from posterior samples of rooted topologies, but they need not coincide. For example, suppose the posterior output comprises the following five trees:

**Table:** 

Tree 1:	((A, B),(((E, F), D), C));
Tree 2:	(((A, B), C),((E, F), D));
Tree 3:	((((A, B), C), D),(E, F));
Tree 4:	(((((A, B), C), D), E), F);
Tree 5:	((A, B),(((E, F), D), C));

The clade (A, B) appears on all the trees and so is included in the consensus tree with probability one. Similarly, the clade (A, B, C) appears on three trees (Tree 2, Tree 3, and Tree 4) and so appears in the consensus tree with support 0.6. Continuing in this fashion, the consensus tree is completed by incorporating the clades (E, F) and (D, E, F) that appear with support 0.8 and 0.6, respectively. Hence, the root position on the consensus tree (displayed in fig. 8) separates the taxa A, B, C from D, E, F. On the other hand, the posterior for root splits is given in table 5. Clearly the posterior modal root split is (A, B): (C, D, E, F) which does not match the root split (A, B, C): (D, E, F) on the consensus tree.

**Table 5.** Posterior for Root Splits in Illustrative Example.

Root Split	Count	Probability
(A, B): (C, D, E, F)	2	0.4
(A, B, C): (D, E, F)	1	0.2
(E, F): (A, B, C, D)	1	0.2
(F): (A, B, C, D, E)	1	0.2

**Fig. 8.**
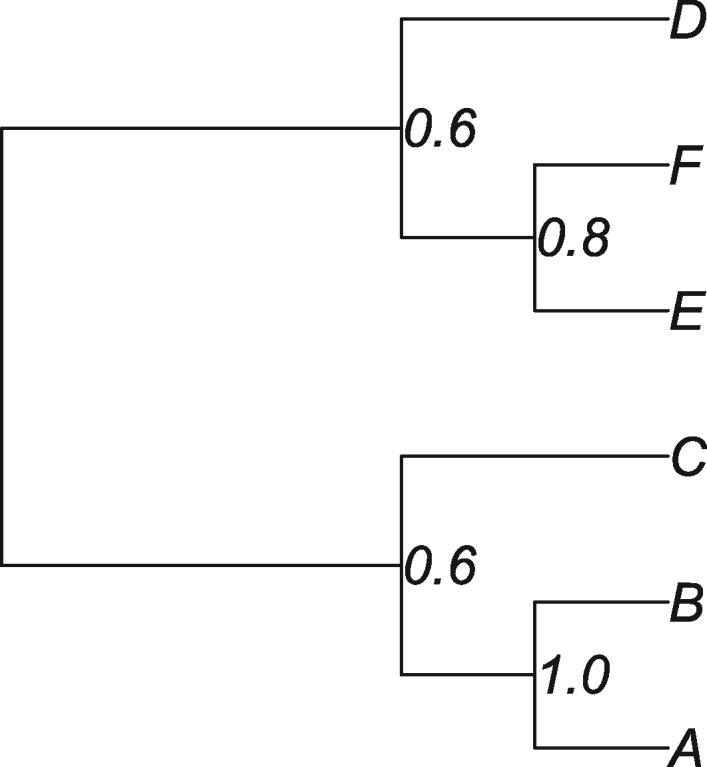
Majority rule consensus tree for illustrative example.

## Supplementary Material

Supplementary data are available at *Molecular Biology and Evolution* online.

## Supplementary Material

Supplementary Data
