# Supplementary material for: The Effect of Nonreversibility on Inferring Rooted Phylogenies
Source: Mol Biol Evol. 2017 Nov 15;35(4):984–1002. doi: 10.1093/molbev/msx294 (PMC5889004; doi:10.1093/molbev/msx294)
Supplement: Supplementary Data [file msx294_supp.zip › software_link.html]

## Software for the paper "The effect of non-reversibility on inferring rooted phylogenies"

Download a zip file containing java executable and instructions for use.
