# Supplementary material for: The Effect of Nonreversibility on Inferring Rooted Phylogenies
Source: Mol Biol Evol. 2017 Nov 15;35(4):984–1002. doi: 10.1093/molbev/msx294 (PMC5889004; doi:10.1093/molbev/msx294)
Supplement: Supplementary Data [file msx294_supp.zip › SupplMat.pdf]

Supplementary Material for Cherlin *et al.* (2017)

1 Approximation of the Bayes factor

In the analyses of the yeast and tree of life data sets, we computed a log Bayes factor to assess whether the Yule or structured uniform prior for the rooted topology was most consistent with the data.

Denote the parameters of the NR (or NR2) substitution model, the across site rate heterogeneity parameter, and the branch lengths by $\boldsymbol{\theta}$. Let M represent the model for the data conditional on $\boldsymbol{\theta}$ and the rooted topology τ. Recalling that we assume that $\boldsymbol{\theta}$ and τ are independent *a priori*, denote by P_θ the joint prior for $\boldsymbol{\theta}$, and by $P_{\tau,Y}$ or $P_{\tau,S}$ the Yule or structured uniform prior for rooted topologies, respectively. We can express the Bayes factor comparing the two priors as

$$B_{YS} = \frac{p(\text{data}|M, P_\theta, P_{\tau,Y})}{p(\text{data}|M, P_\theta, P_{\tau,S})}.$$

In this expression,

$$p(\text{data}|M, P_\theta, P_{\tau,Y}) = \frac{p(\text{data}|\tau, M, P_\theta)p(\tau|P_{\tau,Y})}{p(\tau|\text{data}, M, P_\theta, P_{\tau,Y})}$$

in which

$$p(\text{data}|\tau, M, P_\theta) = \int p(\text{data}|\boldsymbol{\theta}, \tau, M)p(\boldsymbol{\theta}|P_\theta)\, d\theta$$

and similarly for $p(\text{data}|M, P_\theta, P_{\tau,S})$. It follows that

$$\begin{aligned} B_{YS} &= \frac{p(\tau|P_{\tau,Y})}{p(\tau|P_{\tau,S})} \times \frac{p(\text{data}|\tau, M, P_\theta)}{p(\text{data}|\tau, M, P_\theta)} \times \frac{p(\tau|\text{data}, M, P_\theta, P_{\tau,S})}{p(\tau|\text{data}, M, P_\theta, P_{\tau,Y})} \\ &= \frac{p(\tau|P_{\tau,Y})}{p(\tau|P_{\tau,S})} \times \frac{p(\tau|\text{data}, M, P_\theta, P_{\tau,S})}{p(\tau|\text{data}, M, P_\theta, P_{\tau,Y})} \end{aligned}$$

where the likelihood ratio cancels because the model and the prior for $\boldsymbol{\theta}$ do not differ. Since the left-hand-side of this expression does not depend on τ, it must hold for any topology. In practice, we follow the recommendation of Chib (1995) and use the approximation

$$\hat{B}_{YS} = \frac{p(\tau^*|P_{\tau,Y})}{p(\tau^*|P_{\tau,S})} \times \frac{\hat{p}(\tau^*|\text{data}, M, P_\theta, P_{\tau,S})}{\hat{p}(\tau^*|\text{data}, M, P_\theta, P_{\tau,Y})}$$

where τ^* is a tree with high posterior support under both priors and the posterior ordinates $\hat{p}(\tau^*|\text{data}, M, P_\theta, P_{\tau,\cdot})$ are approximated using the posterior samples of trees. For example, $\hat{p}(\tau^*|\text{data}, M, P_\theta, P_{\tau,Y})$ is the proportion of posterior draws under the Yule prior where topology τ^* was sampled.

2 Supplemetary Figures and Tables

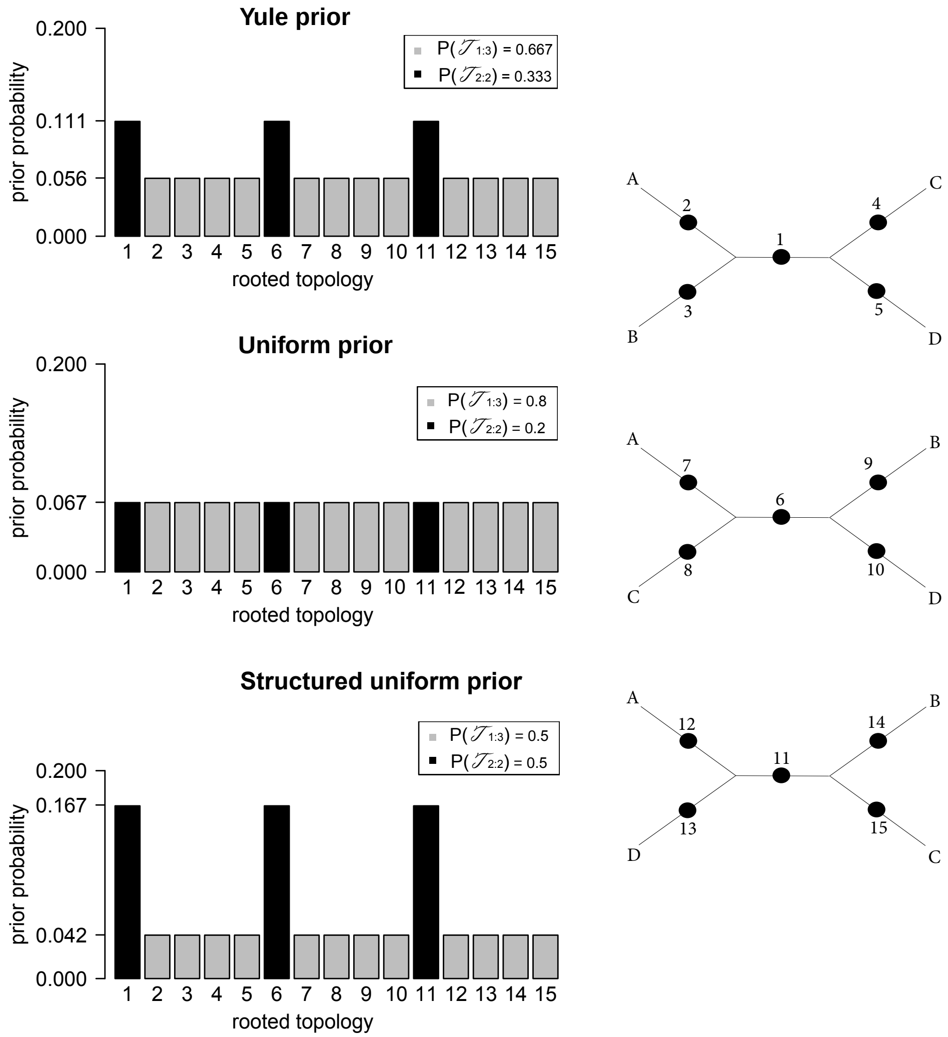

Supplementary Figure 1: Probability assigned to each rooted topology and to the subsets $\mathcal{T}_{1:3}$ and $\mathcal{T}_{2:2}$ by three topological priors: Yule, uniform and structured uniform. The Yule and structured uniform priors assign higher probabilities to each of the balanced trees than to any of the caterpillar trees rooted on a pendant edge.

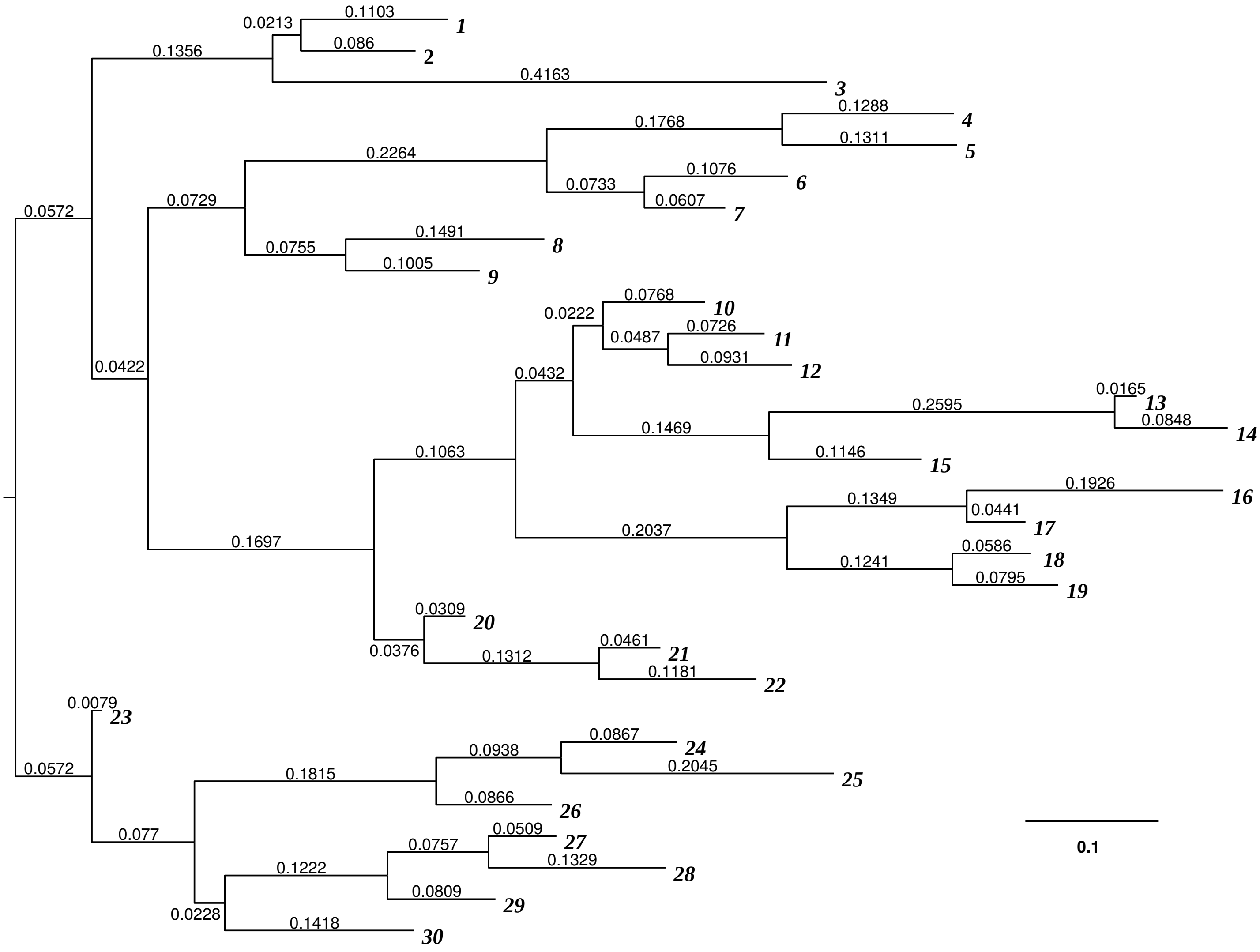

Supplementary Figure 2: A rooted random 30-taxon tree for simulating the data with the branch lengths indicated. The tree is generated under the Yule birth process, with the branch lengths simulated from Ga(2,20). The lengths of the branches adjacent to the root are simulated from Ga(1,20) such that the combined length of these two branches corresponds to a realisation from a Ga(2,20) distribution.

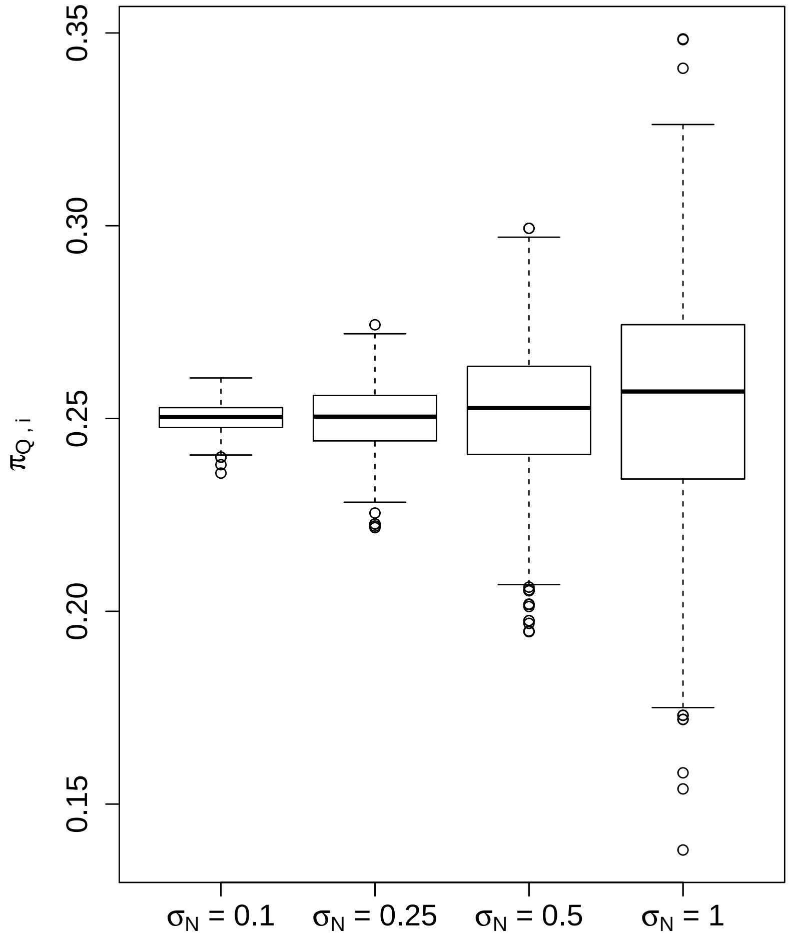

Supplementary Figure 3: Box-plot of the prior for the first element of the stationary distribution simulated with $\boldsymbol{\pi} = (0.25, 0.25, 0.25, 0.25)$, $\kappa = 2$, $\sigma_R = 0.1$ and different values of the perturbation standard deviation σ_N. The priors for the rest of the elements of the stationary distribution are the same due to symmetrys. Increasing the value of σ_N clearly increases the spread in the prior for the stationary probabilities.

$\sigma_N = 0$, NR model, Yule prior

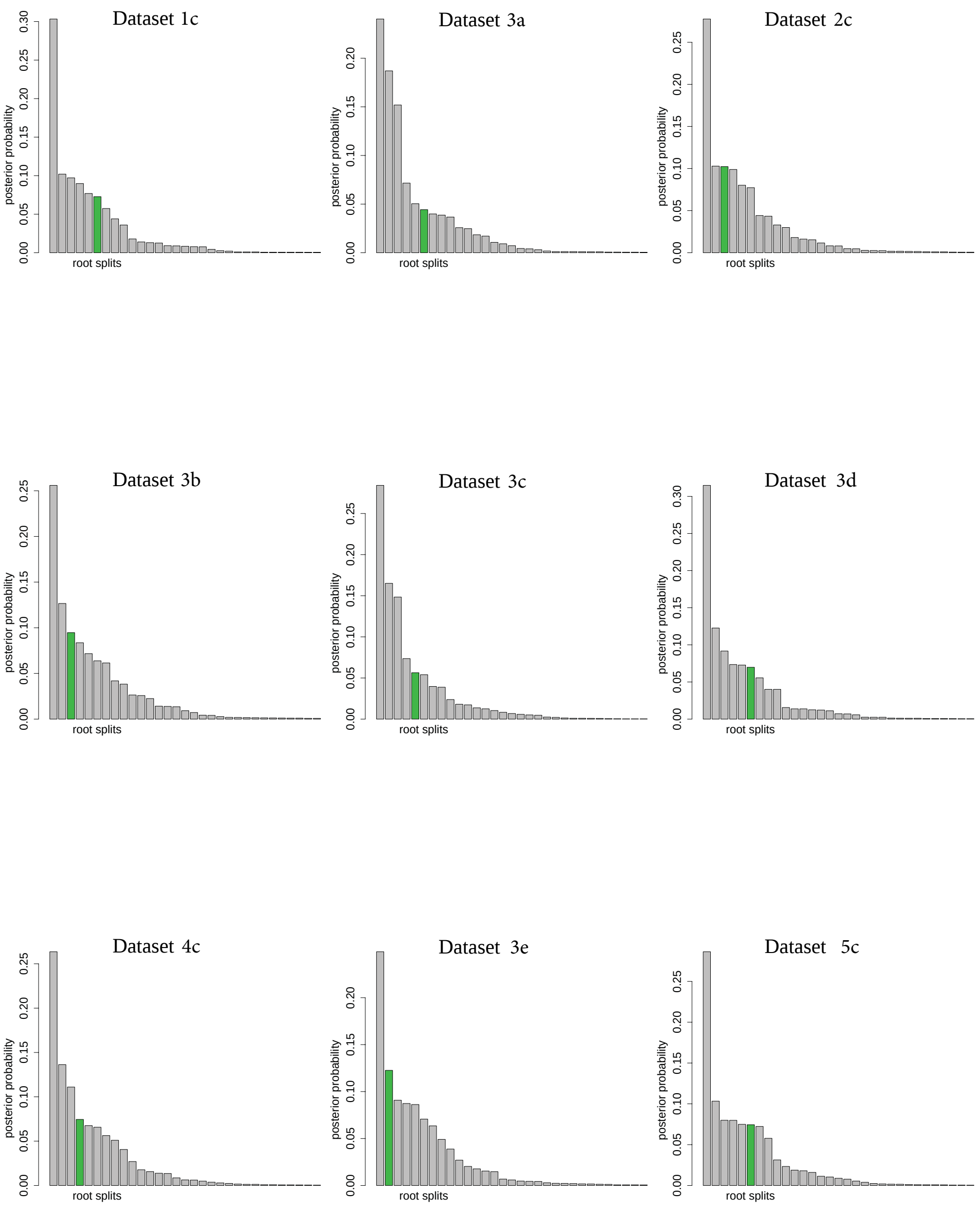

$\sigma_N = 0.1$, NR model, Yule prior

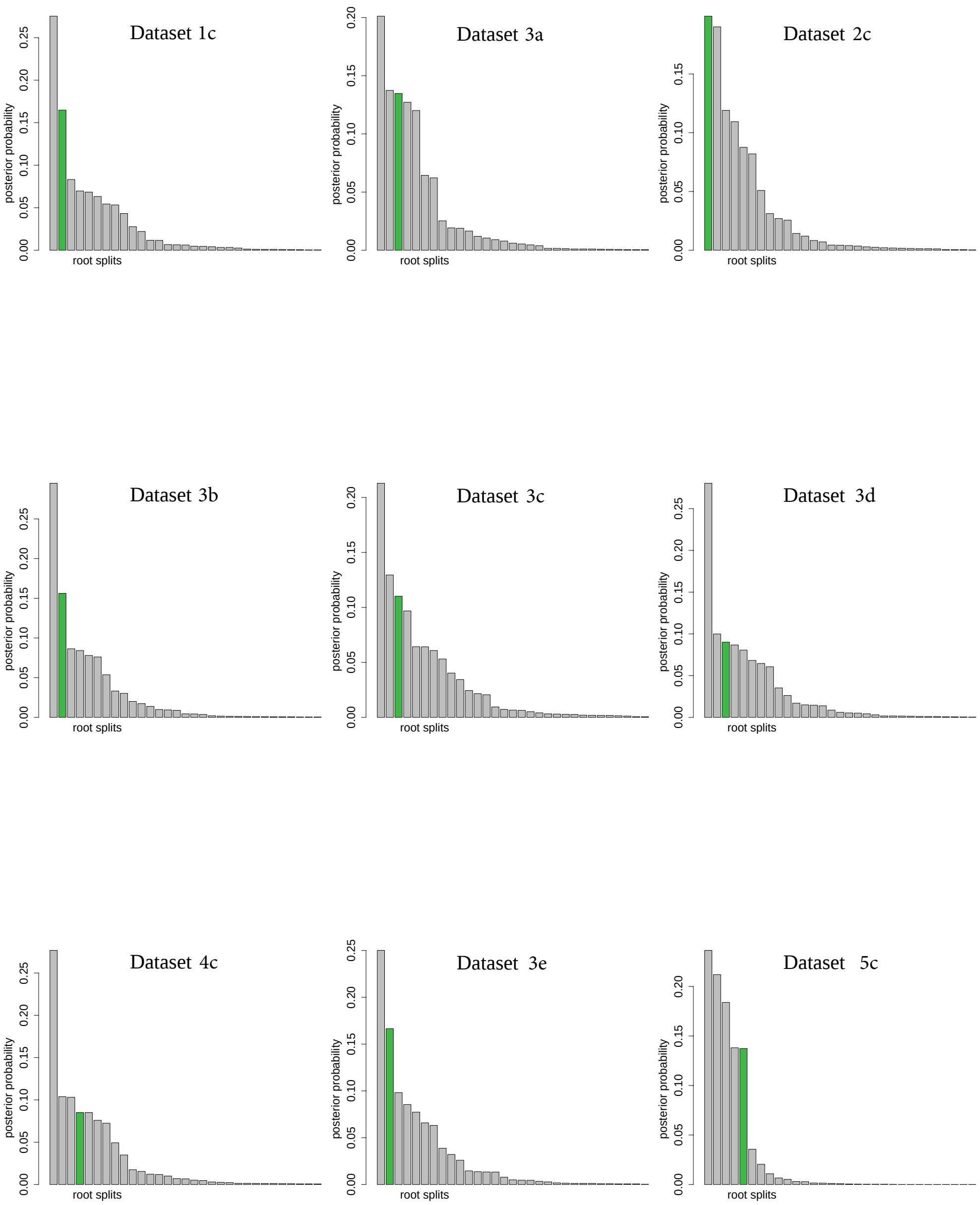

σ_N = 0.25, NR model, Yule prior

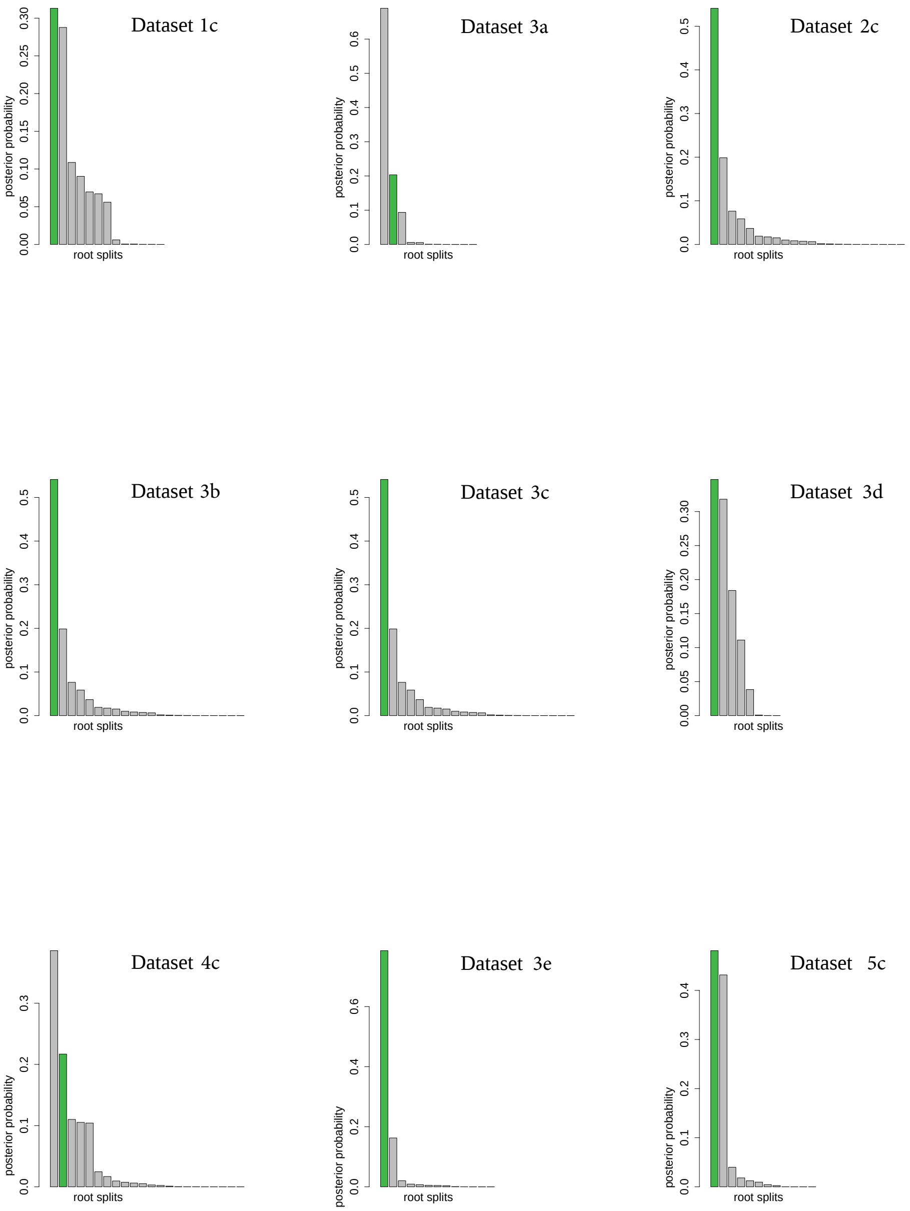

σ_N = 0.5, NR model, Yule prior

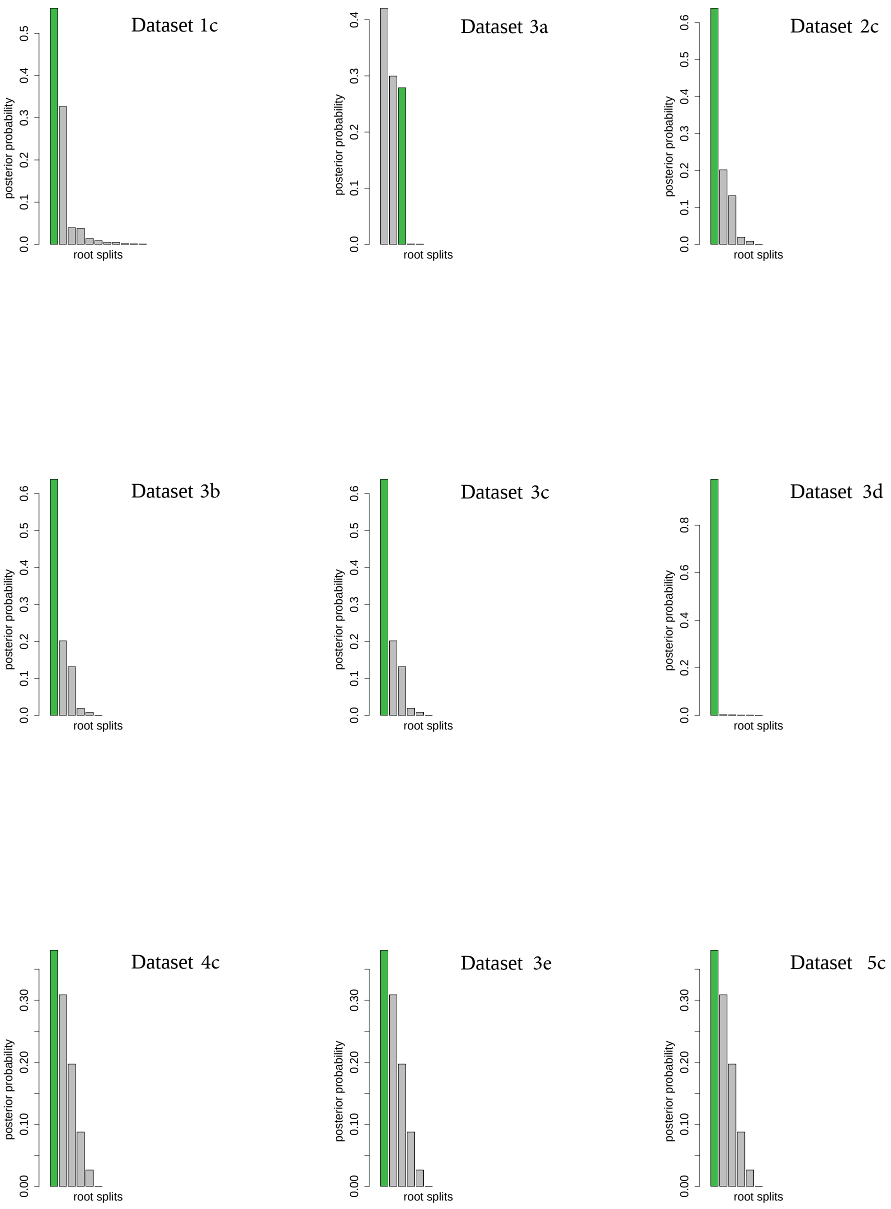

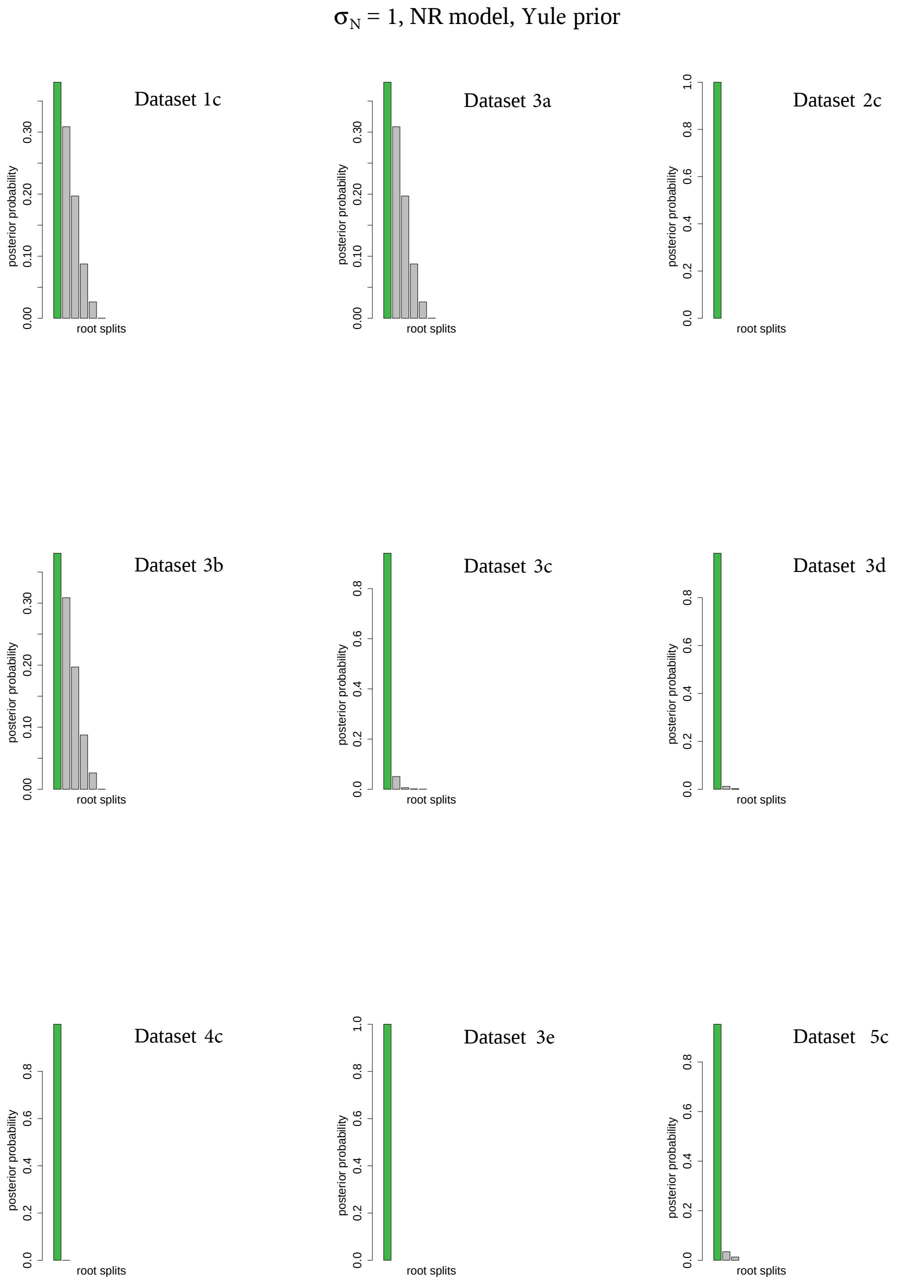

Supplementary Figure 4: Posterior distribution of the root splits for a representative selection of data sets simulated under the NR2 model with different values of σ_N and analysed with the NR model and Yule prior. Different bars on the plots represent different root splits on the posterior distribution of trees (ordered by posterior probabilities). On each plot the green bar represents the true root split.

σ_N = 0, NR model, Yule prior

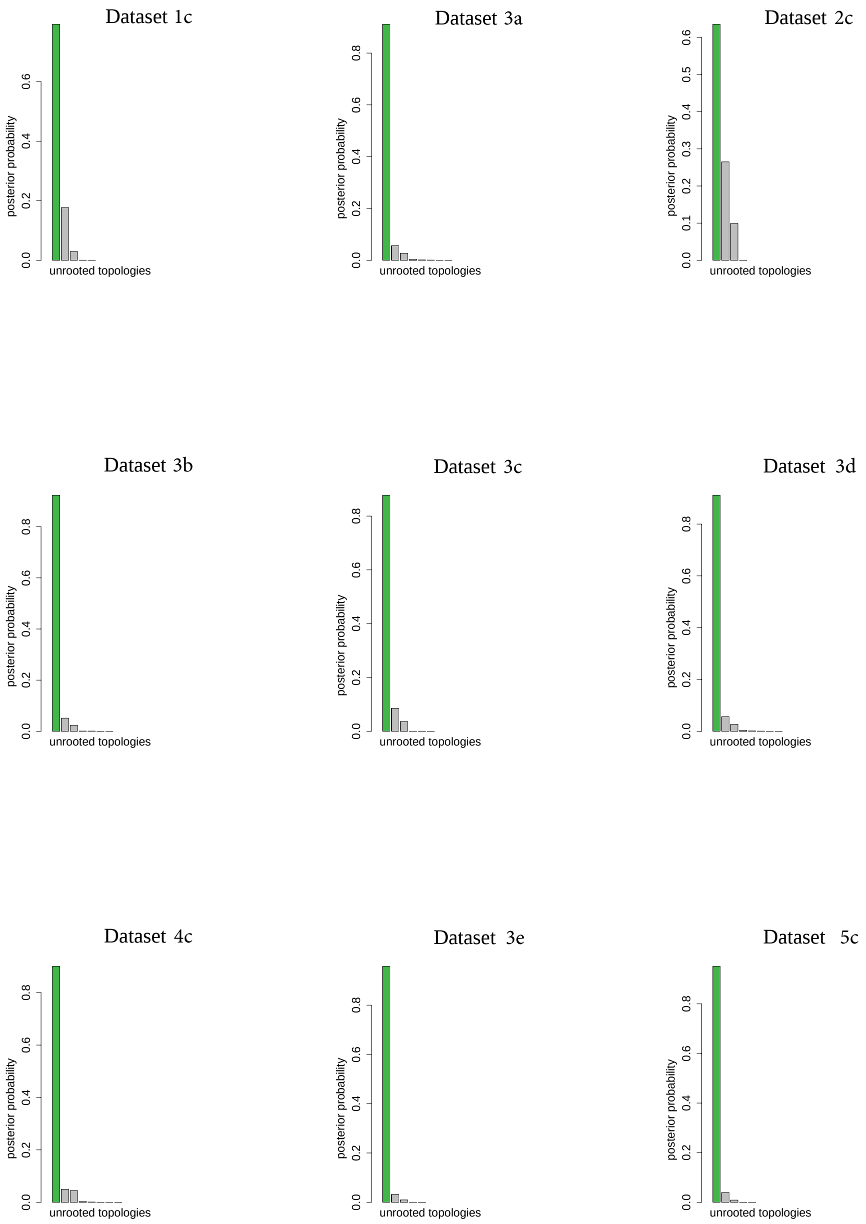

σ_N = 0.1, NR model, Yule prior

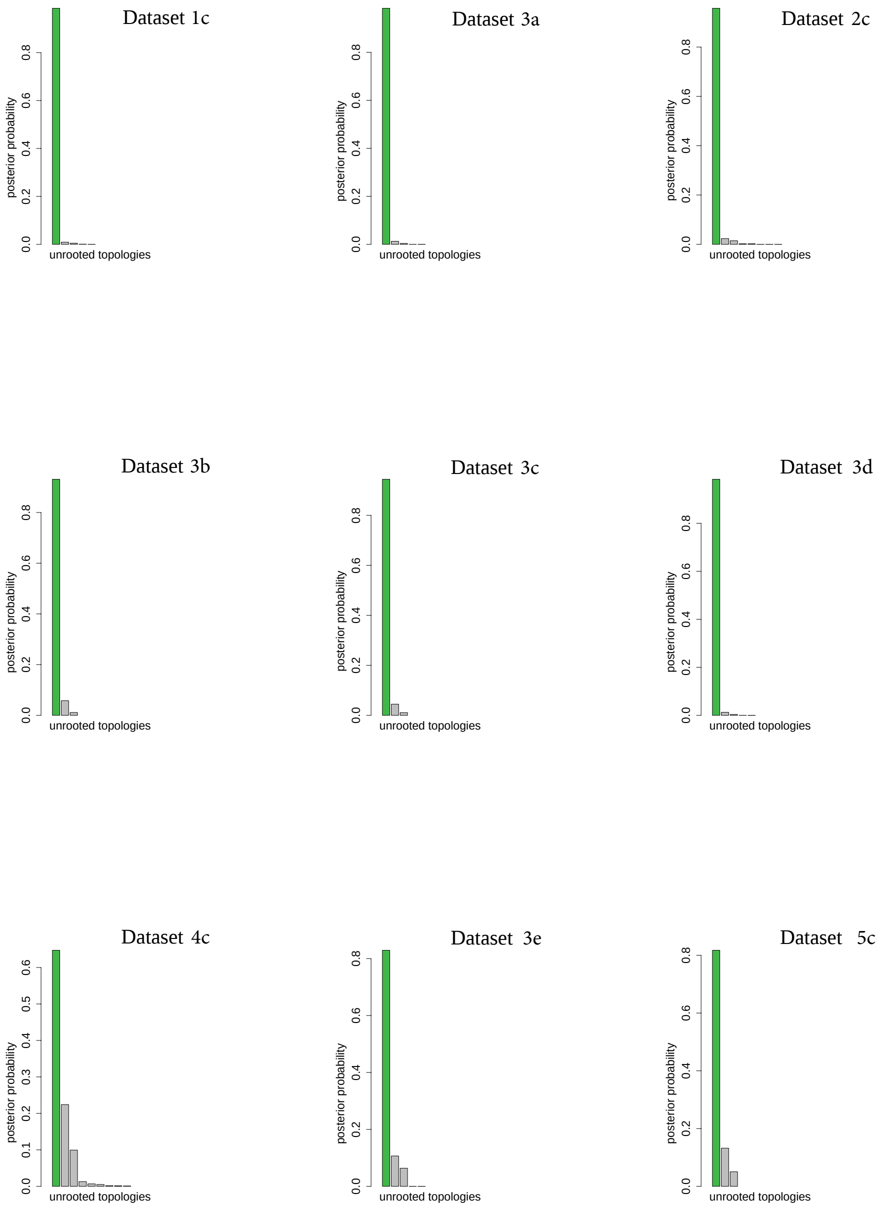

σ_N = 0.25, NR model, Yule prior

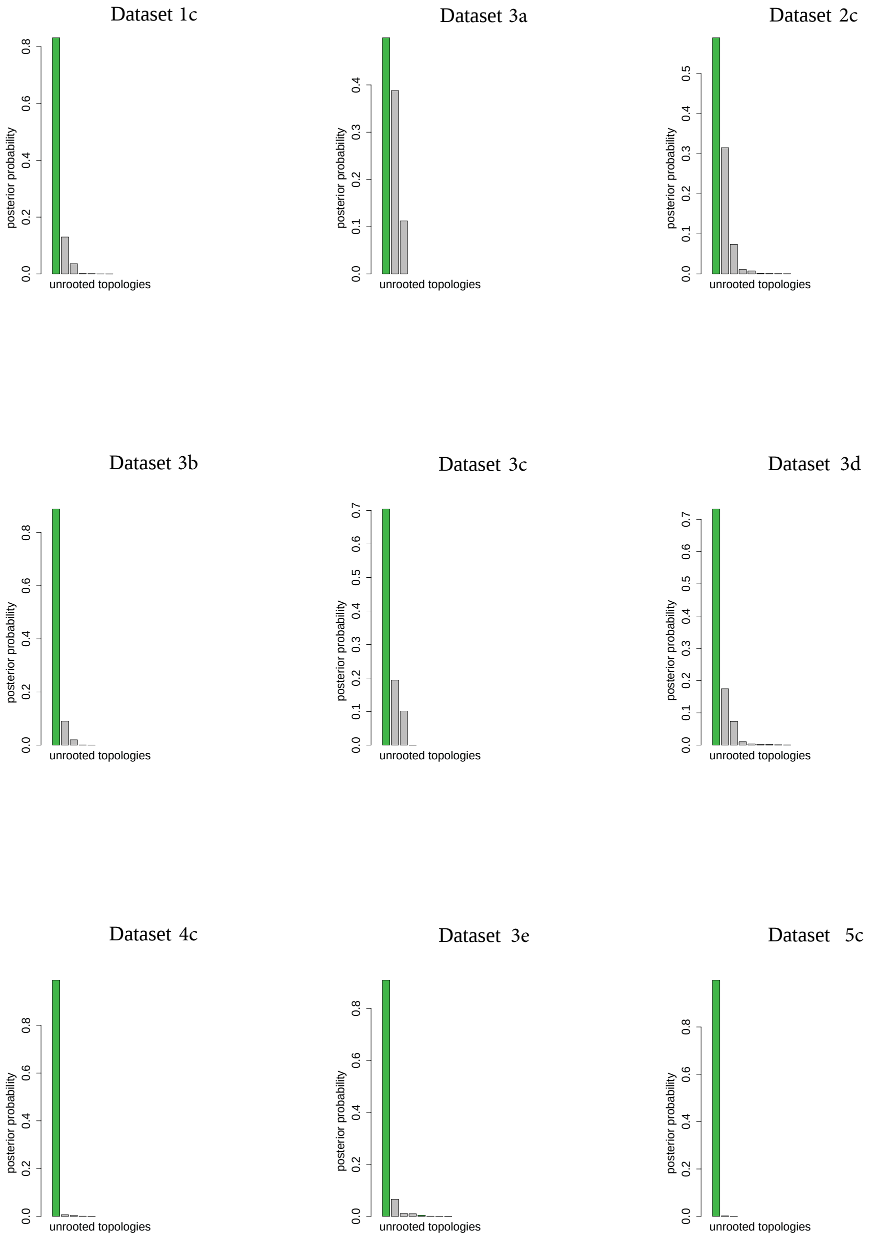

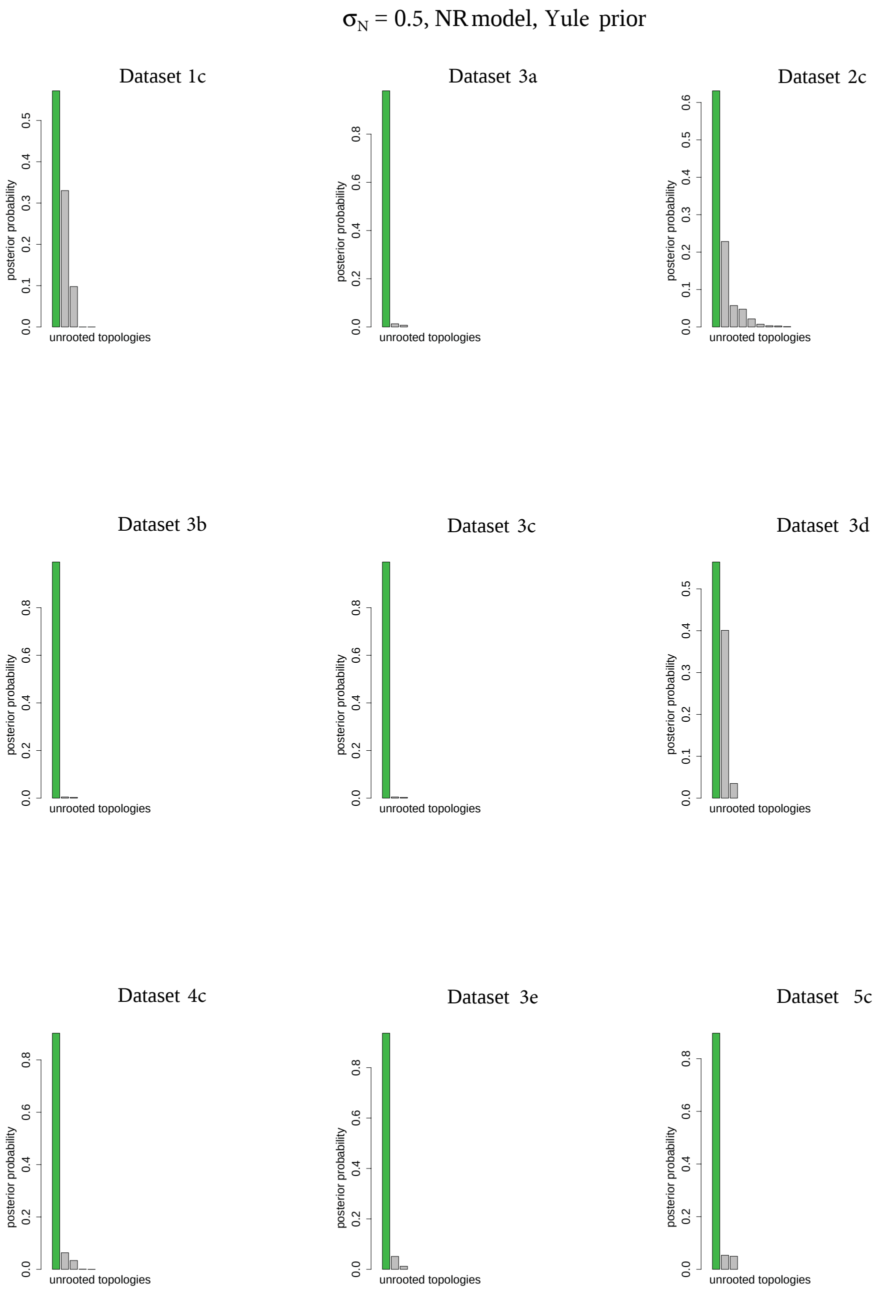
σN = 0.5, NR model, Yule prior
Dataset 1c
Dataset 3a
Dataset 2c
Dataset 3b
Dataset 3c
Dataset 3d
Dataset 4c
Dataset 3e
Dataset 5c
posterior probability
unrooted topologies

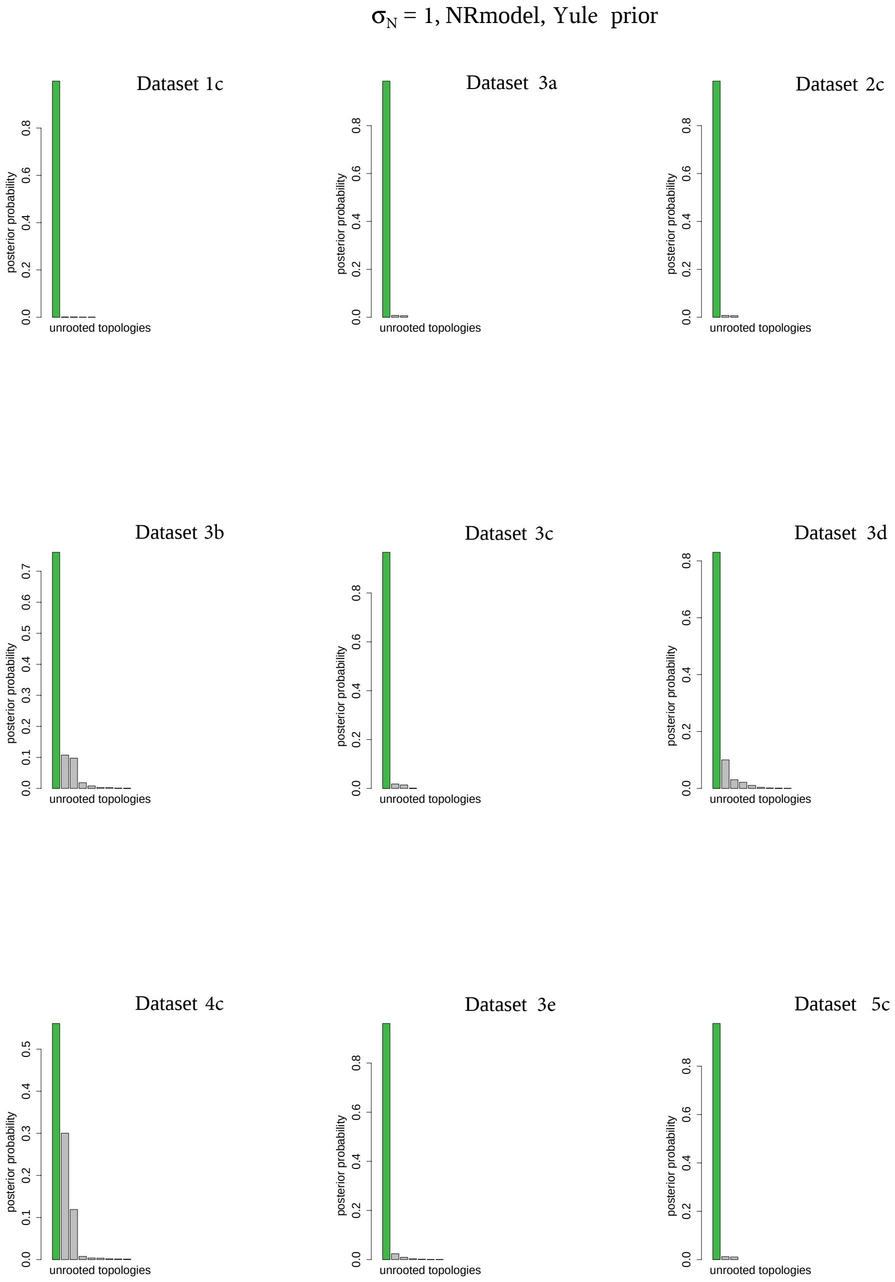

Supplementary Figure 5: Posterior distribution of the unrooted topologies for a representative selection of data sets simulated under the NR2 model with different values of σ_N and analysed with the NR model and Yule prior. Different bars on the plots represent different unrooted topologies on the posterior distribution of trees (ordered by posterior probabilities). On each plot the green bar represents the true unrooted topology.

Supplementary Table 1: Marginal posterior probabilities of the correct root split for the simulations from the NR2 model, analysed under the NR model with the structured uniform prior. The posterior means for Huelsenbeck's I statistic are indicated in parentheses. When the correct root split is a modal root split, the corresponding marginal posterior probability appears in bold.

Data Set	$\sigma_N = 0$	$\sigma_N = 0.1$	$\sigma_N = 0.25$	$\sigma_N = 0.5$	$\sigma_N = 1.0$
1a	0.06 (0.04)	0.12 (0.05)	0.37 (0.22)	**0.95** (0.35)	**1.00** (1.05)
1b	0.06 (0.04)	0.08 (0.06)	0.31 (0.22)	**0.89** (0.34)	**1.00** (1.08)
1c	0.07 (0.02)	0.13 (0.04)	**0.43** (0.21)	0.27 (0.30)	**1.00** (1.05)
1d	0.08 (0.02)	0.08 (0.05)	**0.66** (0.21)	**0.92** (0.31)	**1.00** (1.07)
1e	0.09 (0.03)	0.09 (0.05)	**0.72** (0.27)	**0.75** (0.35)	**1.00** (1.05)
2a	0.10 (0.04)	0.07 (0.04)	0.11 (0.15)	**0.94** (0.42)	**0.96** (0.82)
2b	0.07 (0.05)	0.12 (0.05)	**0.48** (0.16)	**0.97** (0.43)	**1.00** (0.90)
2c	0.09 (0.03)	**0.17** (0.06)	**0.54** (0.18)	**0.80** (0.50)	**1.00** (0.92)
2d	0.08 (0.04)	0.08 (0.03)	**0.30** (0.19)	**0.93** (0.45)	**1.00** (0.88)
2e	0.09 (0.02)	0.11 (0.06)	**0.32** (0.19)	**0.98** (0.44)	**1.00** (0.86)
3a	0.04 (0.06)	0.05 (0.08)	0.40 (0.36)	**0.44** (0.49)	**0.99** (0.75)
3b	0.08 (0.04)	0.16 (0.05)	0.26 (0.31)	**0.98** (0.50)	**1.00** (0.74)
3c	0.06 (0.05)	0.07 (0.05)	**0.74** (0.33)	**0.96** (0.44)	**0.96** (0.78)
3d	0.07 (0.03)	0.08 (0.03)	**0.50** (0.32)	**0.99** (0.45)	**0.99** (0.73)
3e	0.11 (0.04)	**0.16** (0.04)	**0.61** (0.32)	**0.97** (0.45)	**1.00** (0.73)
4a	0.08 (0.06)	0.08 (0.04)	0.15 (0.20)	0.40 (0.34)	**1.00** (0.77)
4b	0.08 (0.02)	0.07 (0.03)	0.16 (0.16)	0.30 (0.38)	**0.99** (0.79)
4c	0.07 (0.06)	0.09 (0.03)	0.32 (0.17)	**0.54** (0.35)	**1.00** (0.75)
4d	0.10 (0.02)	0.17 (0.06)	0.13 (0.17)	**0.47** (0.37)	**0.98** (0.73)
4e	0.08 (0.02)	0.08 (0.02)	**0.49** (0.14)	**0.53** (0.34)	**0.99** (0.75)
5a	0.08 (0.03)	**0.23** (0.10)	**0.53** (0.34)	**0.95** (0.50)	**1.00** (1.05)
5b	0.07 (0.02)	0.13 (0.05)	0.31 (0.37)	**0.95** (0.51)	**1.00** (1.02)
5c	0.07 (0.03)	0.21 (0.12)	0.25 (0.32)	**0.94** (0.46)	**0.98** (0.99)
5d	0.09 (0.03)	0.10 (0.08)	**0.52** (0.36)	**0.97** (0.45)	**1.00** (1.04)
5e	0.08 (0.02)	0.10 (0.06)	**0.40** (0.32)	**0.77** (0.45)	**0.99** (1.00)

Supplementary Table 2: Marginal posterior probabilities of the correct root split for the simulations from the NR2 model, analysed under the NR2 model with the Yule prior. The posterior means for Huelsenbeck's I statistic are indicated in parentheses. When the correct root split is a modal root split, the corresponding marginal posterior probability appears in bold.

Data Set	$\sigma_N = 0$	$\sigma_N = 0.1$	$\sigma_N = 0.25$	$\sigma_N = 0.5$	$\sigma_N = 1.0$
1a	0.07 (0.24)	0.11 (0.04)	**0.63** (0.23)	**0.92** (0.35)	**0.99** (1.03)
1b	0.05 (0.04)	0.10 (0.05)	**0.57** (0.23)	**0.79** (0.34)	**1.00** (1.10)
1c	0.06 (0.02)	0.16 (0.06)	**0.33** (0.22)	**0.55** (0.31)	**1.00** (1.00)
1d	0.09 (0.02)	0.07 (0.03)	**0.64** (0.22)	**0.93** (0.31)	**1.00** (1.04)
1e	0.09 (0.02)	0.09 (0.04)	**0.86** (0.28)	**0.70** (0.35)	**1.00** (1.03)
2a	0.09 (0.25)	0.08 (0.03)	0.07 (0.16)	**0.87** (0.41)	**0.95** (0.76)
2b	0.07 (0.05)	0.09 (0.02)	**0.58** (0.17)	**0.98** (0.43)	**1.00** (0.85)
2c	0.10 (0.02)	0.17 (0.05)	**0.57** (0.19)	**0.64** (0.49)	**1.00** (0.87)
2d	0.08 (0.03)	0.09 (0.02)	0.20 (0.19)	**0.88** (0.44)	**1.00** (0.82)
2e	0.09 (0.02)	0.10 (0.04)	**0.41** (0.19)	**0.99** (0.44)	**1.00** (0.80)
3a	0.05 (0.05)	0.13 (0.08)	0.20 (0.36)	0.29 (0.49)	**0.98** (0.69)
3b	0.09 (0.02)	0.17 (0.05)	**0.48** (0.31)	**0.99** (0.49)	**1.00** (0.70)
3c	0.07 (0.04)	0.10 (0.04)	**0.69** (0.33)	**0.92** (0.44)	**0.95** (0.74)
3d	0.08 (0.02)	0.09 (0.03)	**0.34** (0.32)	**0.99** (0.45)	**0.99** (0.68)
3e	0.10 (0.03)	0.16 (0.04)	**0.79** (0.32)	**0.96** (0.45)	**1.00** (0.68)
4a	0.06 (0.07)	0.07 (0.03)	0.09 (0.21)	**0.63** (0.33)	**0.99** (0.77)
4b	0.07 (0.02)	0.08 (0.02)	0.13 (0.17)	0.19 (0.38)	**0.99** (0.81)
4c	0.07 (0.07)	0.09 (0.03)	0.22 (0.18)	**0.37** (0.35)	**1.00** (0.74)
4d	0.11 (0.02)	0.15 (0.06)	0.28 (0.18)	0.32 (0.38)	**0.99** (0.73)
4e	0.08 (0.02)	0.08 (0.03)	**0.74** (0.14)	**0.37** (0.35)	**0.98** (0.76)
5a	0.08 (0.02)	0.22 (0.10)	**0.34** (0.34)	**0.91** (0.51)	**1.00** (1.03)
5b	0.07 (0.02)	0.13 (0.04)	0.21 (0.37)	**0.92** (0.51)	**0.99** (1.00)
5c	0.07 (0.02)	0.03 (0.13)	**0.48** (0.32)	**0.88** (0.46)	**0.95** (0.94)
5d	0.08 (0.03)	0.18 (0.08)	**0.36** (0.36)	**0.97** (0.45)	**0.99** (1.02)
5e	0.08 (0.02)	0.09 (0.07)	0.23 (0.32)	**0.65** (0.44)	**0.99** (0.98)

Supplementary Table 3: Marginal posterior probabilities of the correct root split for the simulations from the NR2 model, analysed under the NR2 model with the structured uniform prior. The posterior means for Huelsenbeck's I statistic are indicated in parentheses. When the correct root split is a modal root split, the corresponding marginal posterior probability appears in bold.

Data Set	$\sigma_N = 0$	$\sigma_N = 0.1$	$\sigma_N = 0.25$	$\sigma_N = 0.5$	$\sigma_N = 1.0$
1a	0.08 (0.02)	0.11 (0.04)	0.40 (0.23)	**0.95** (0.35)	**1.00** (1.03)
1b	0.07 (0.04)	0.08 (0.05)	0.32 (0.22)	**0.88** (0.34)	**1.00** (1.06)
1c	0.07 (0.03)	0.13 (0.06)	**0.42** (0.22)	0.27 (0.31)	**1.00** (1.00)
1d	0.08 (0.02)	0.08 (0.03)	**0.70** (0.22)	**0.93** (0.31)	**1.00** (1.04)
1e	0.09 (0.02)	0.08 (0.04)	**0.71** (0.28)	**0.75** (0.35)	**1.00** (1.04)
2a	0.09 (0.03)	0.07 (0.03)	0.10 (0.16)	**0.93** (0.41)	**0.96** (0.76)
2b	0.07 (0.05)	0.10 (0.03)	**0.49** (0.17)	**0.98** (0.43)	**1.00** (0.85)
2c	0.09 (0.02)	**0.15** (0.05)	**0.55** (0.19)	**0.80** (0.49)	**1.00** (0.87)
2d	0.09 (0.03)	0.08 (0.02)	**0.29** (0.19)	**0.93** (0.44)	**1.00** (0.82)
2e	0.09 (0.02)	0.11 (0.04)	**0.31** (0.19)	**0.98** (0.44)	**1.00** (0.80)
3a	0.05 (0.05)	0.06 (0.09)	0.40 (0.36)	**0.44** (0.49)	**0.99** (0.69)
3b	0.08 (0.02)	0.17 (0.05)	0.24 (0.31)	**0.98** (0.49)	**1.00** (0.69)
3c	0.06 (0.04)	0.06 (0.05)	**0.77** (0.33)	**0.96** (0.44)	**0.97** (0.74)
3d	0.08 (0.02)	0.08 (0.04)	**0.51** (0.32)	**0.99** (0.45)	**0.99** (0.68)
3e	0.10 (0.03)	**0.16** (0.04)	**0.62** (0.32)	**0.97** (0.45)	**0.99** (0.68)
4a	0.07 (0.07)	0.08 (0.03)	0.14 (0.21)	0.40 (0.33)	**1.00** (0.81)
4b	0.07 (0.02)	0.08 (0.02)	0.15 (0.17)	0.30 (0.38)	**1.00** (0.69)
4c	0.07 (0.07)	0.09 (0.03)	0.32 (0.18)	**0.53** (0.35)	**1.00** (0.74)
4d	0.11 (0.02)	0.16 (0.06)	0.12 (0.18)	**0.46** (0.38)	**0.97** (0.73)
4e	0.08 (0.02)	0.08 (0.03)	**0.48** (0.14)	**0.53** (0.35)	**0.99** (0.76)
5a	0.07 (0.02)	**0.23** (0.10)	**0.52** (0.34)	**0.95** (0.51)	**1.00** (1.03)
5b	0.07 (0.02)	0.12 (0.04)	0.31 (0.37)	**0.96** (0.51)	**1.00** (1.01)
5c	0.08 (0.02)	**0.22** (0.12)	0.25 (0.32)	**0.94** (0.46)	**0.98** (0.95)
5d	0.09 (0.03)	0.10 (0.09)	**0.52** (0.37)	**0.97** (0.45)	**1.00** (1.02)
5e	0.07 (0.02)	0.09 (0.08)	**0.38** (0.32)	**0.76** (0.44)	**1.00** (0.98)

$\sigma_N = 0$, NR model, structured uniform prior

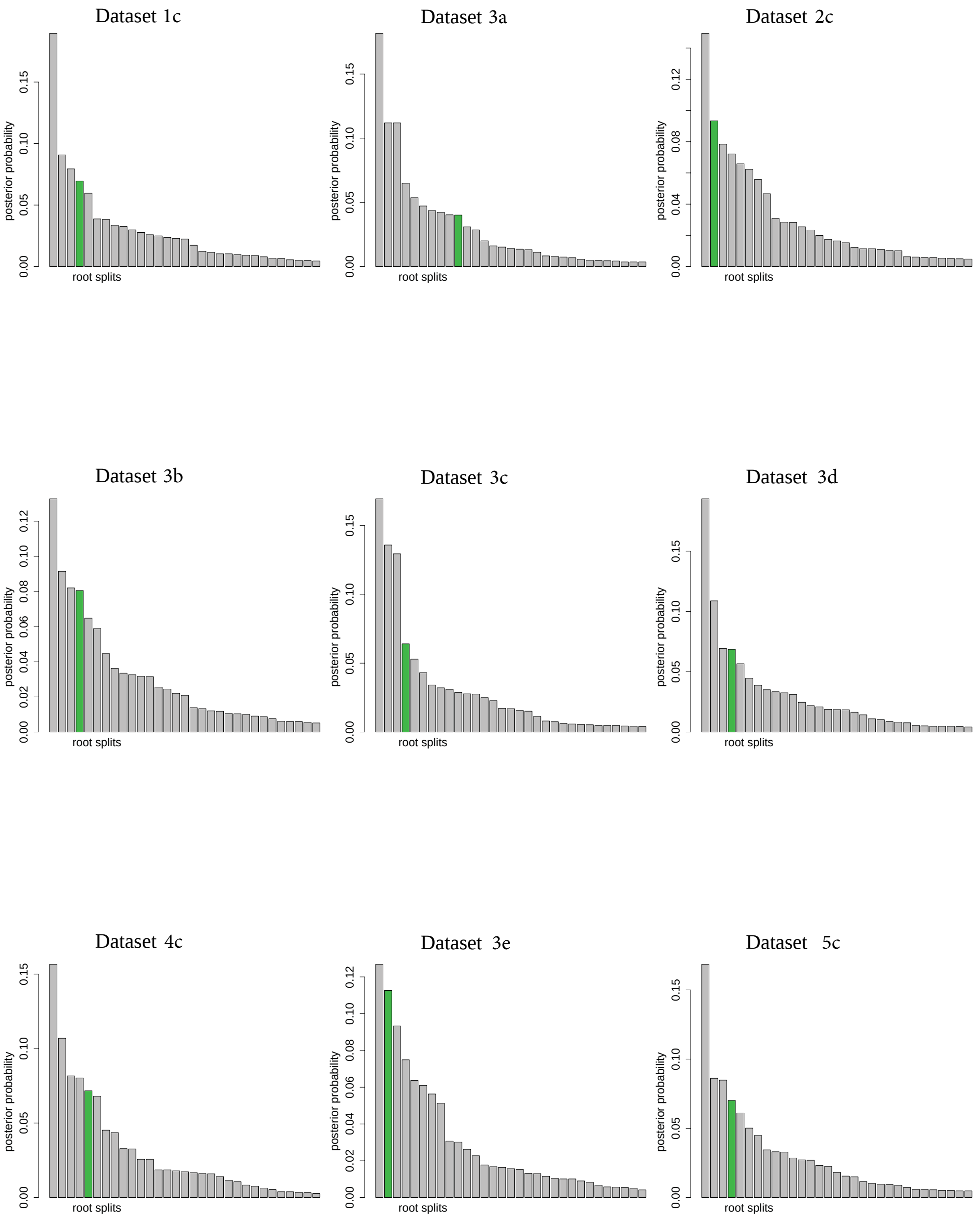

σ_N = 0.1, NR model, structured uniform prior

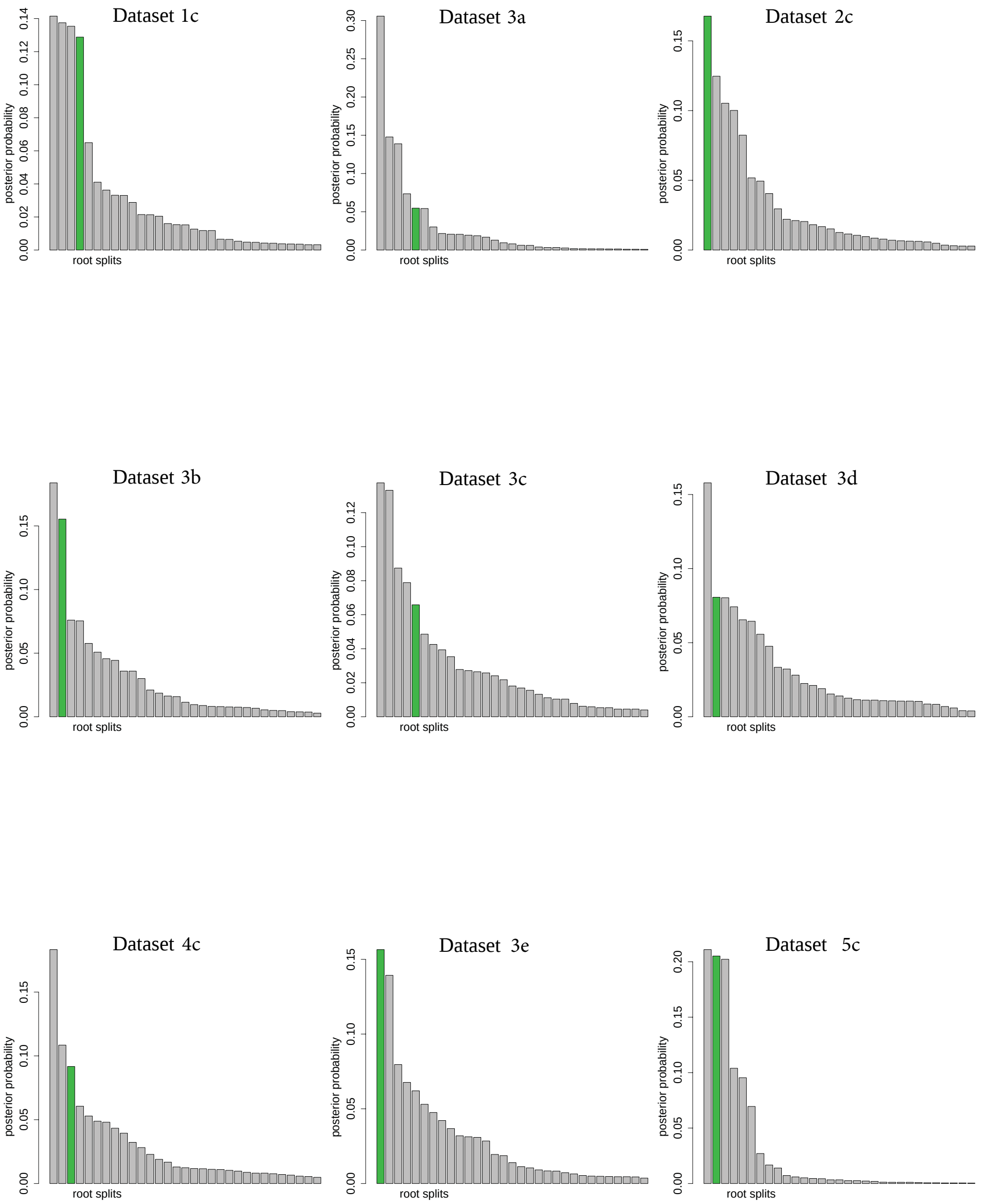

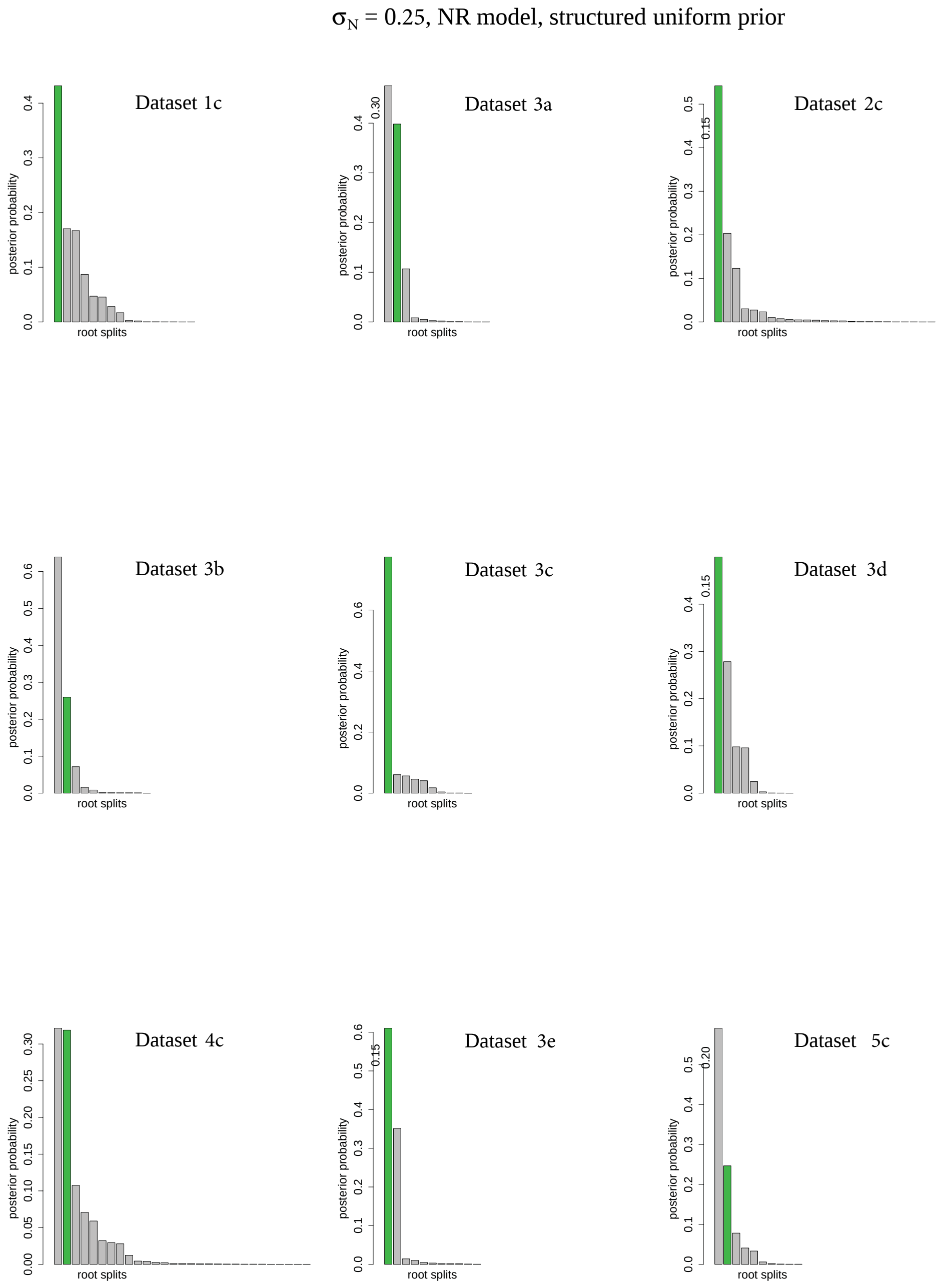
σ_N = 0.25, NR model, structured uniform prior
Dataset 1c
Dataset 3a
Dataset 2c
Dataset 3b
Dataset 3c
Dataset 3d
Dataset 4c
Dataset 3e
Dataset 5c
posterior probability
root splits

$\sigma_N = 0.5$, NR model, structured uniform prior

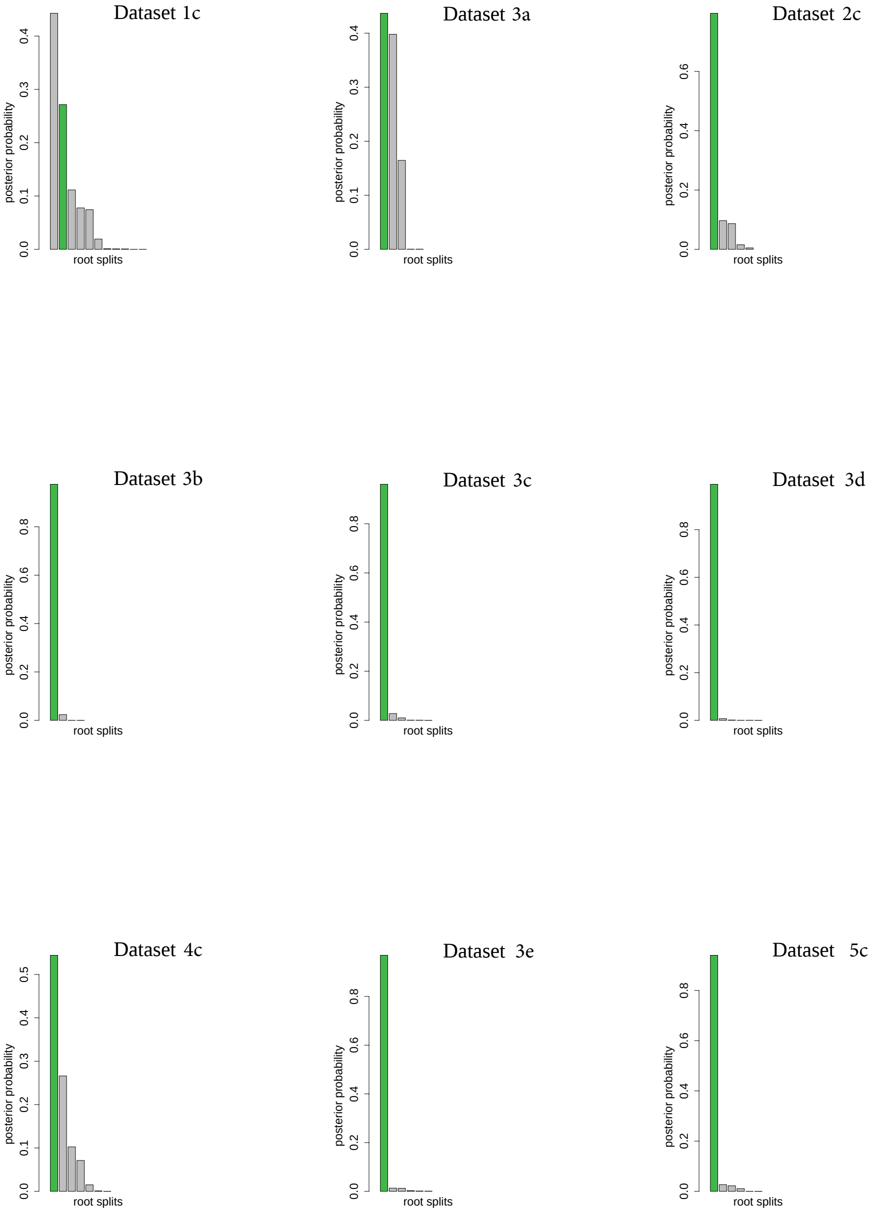

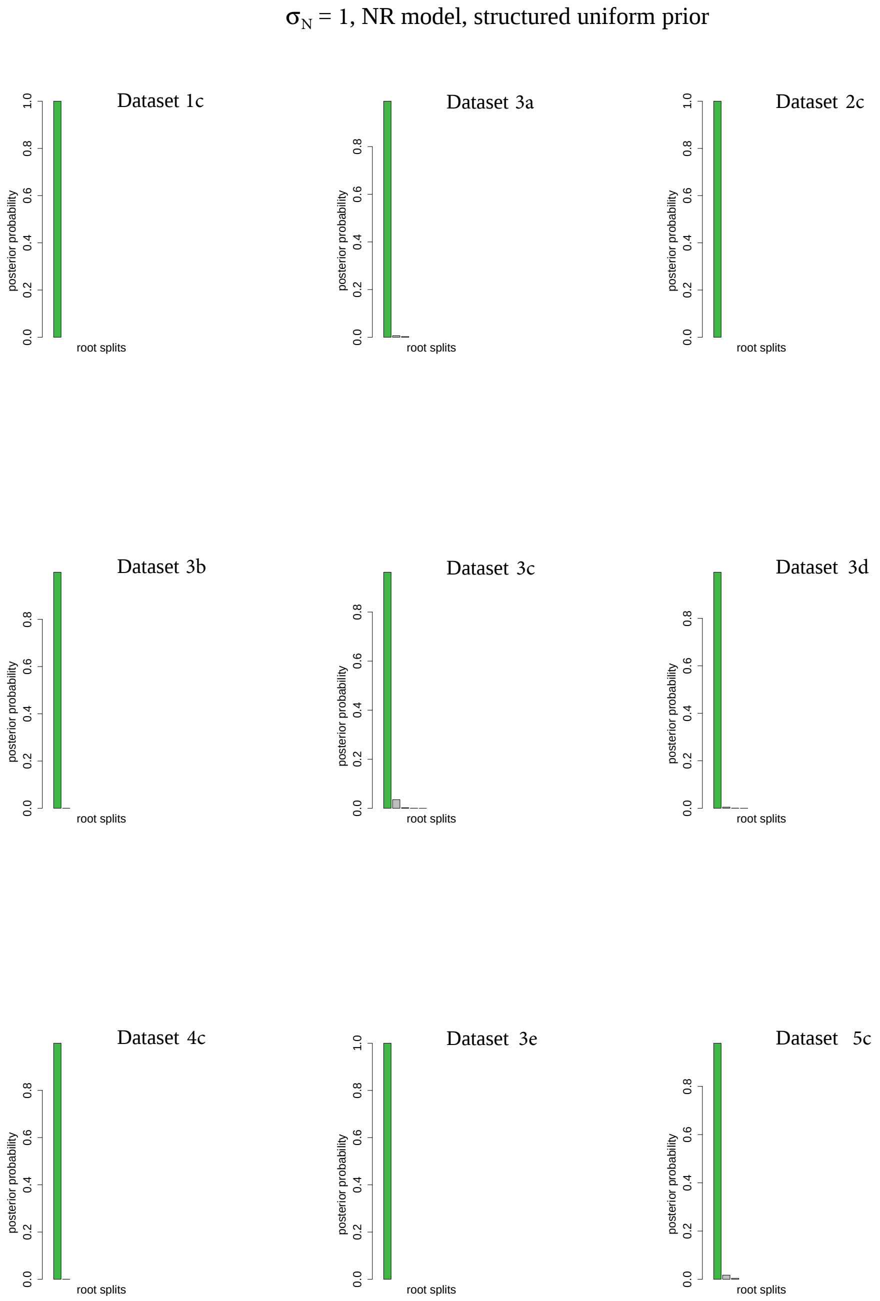

Supplementary Figure 6: Posterior distribution of the root splits for a representative selection of data sets simulated under the NR2 model with different values of σ_N and analysed with the NR model and structured uniform prior. Different bars on the plots represent different root splits on the posterior distribution of trees (ordered by posterior probabilities). On each plot the green bar represents the true root split.

σ_N = 0, NR model, structured uniform prior

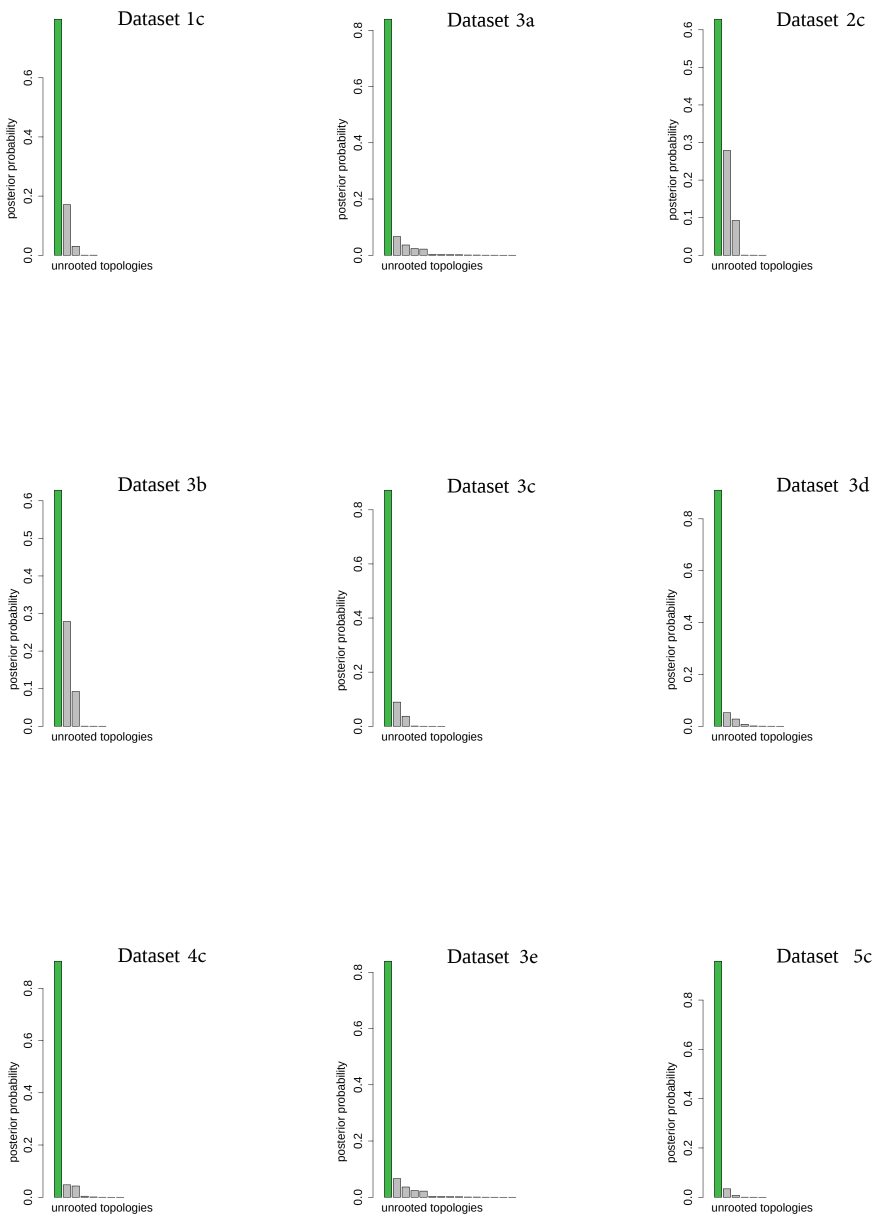

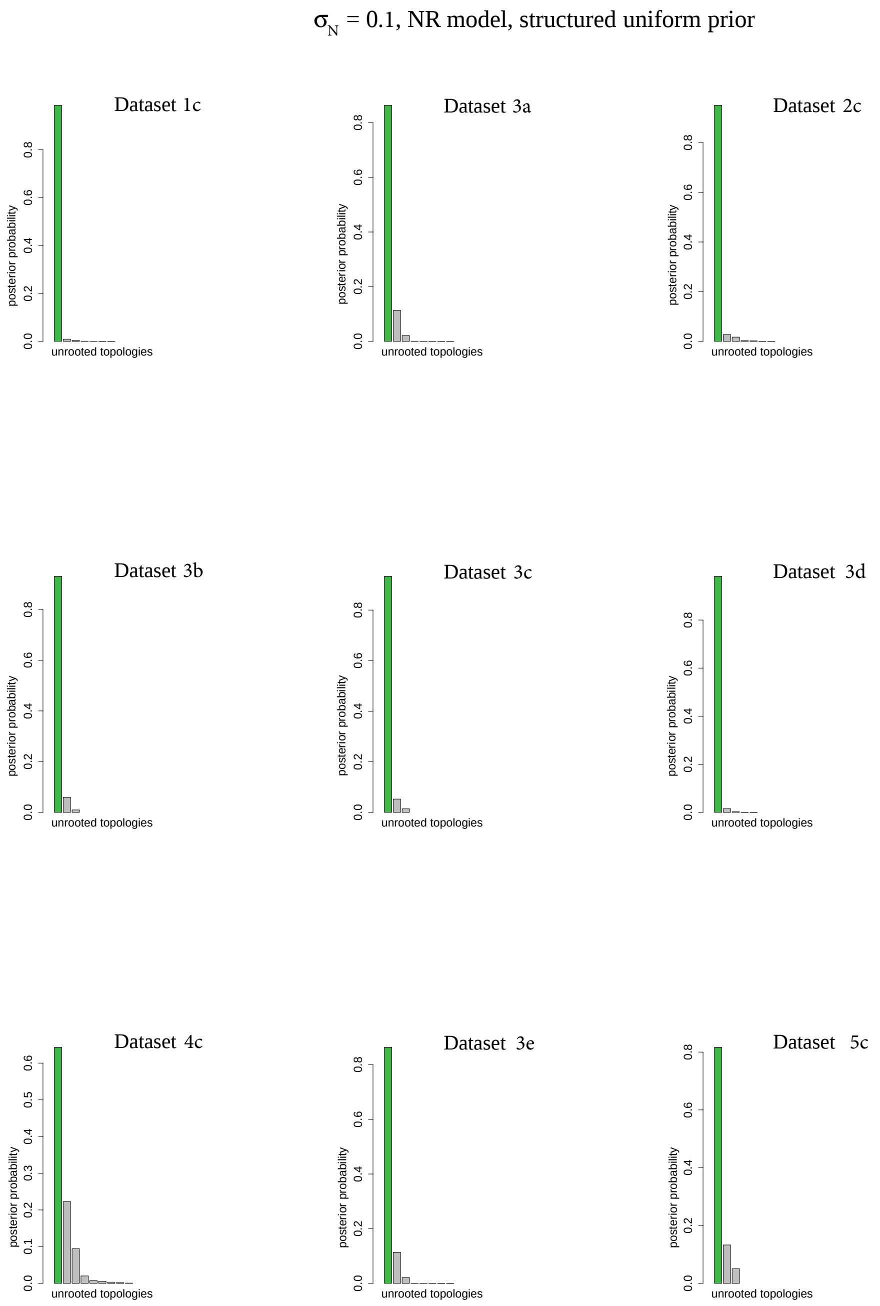

σ_N = 0.1, NR model, structured uniform prior
Dataset 1c
Dataset 3a
Dataset 2c
Dataset 3b
Dataset 3c
Dataset 3d
Dataset 4c
Dataset 3e
Dataset 5c
posterior probability
unrooted topologies

σ_N = 0.25, NR model, structured uniform prior

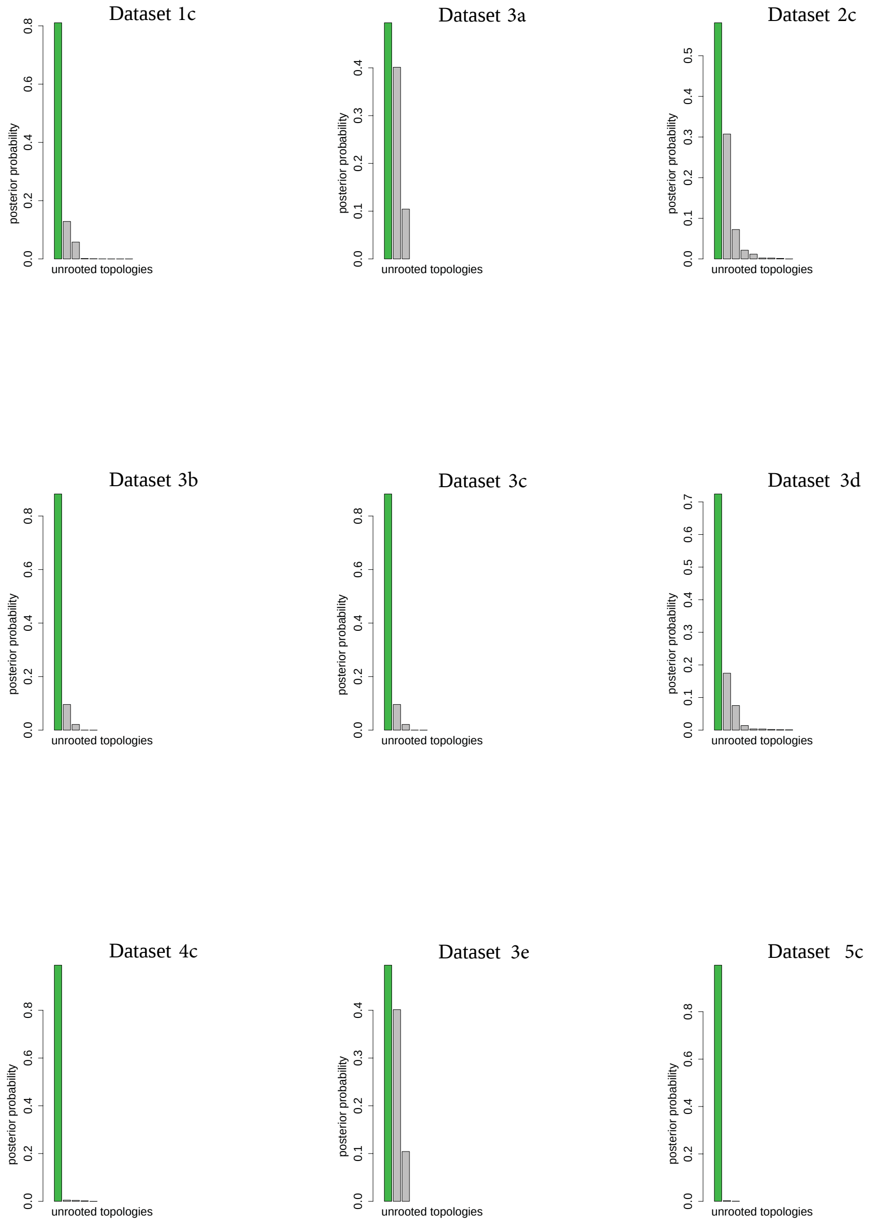

σ_N = 0.5, NR model, structured uniform prior

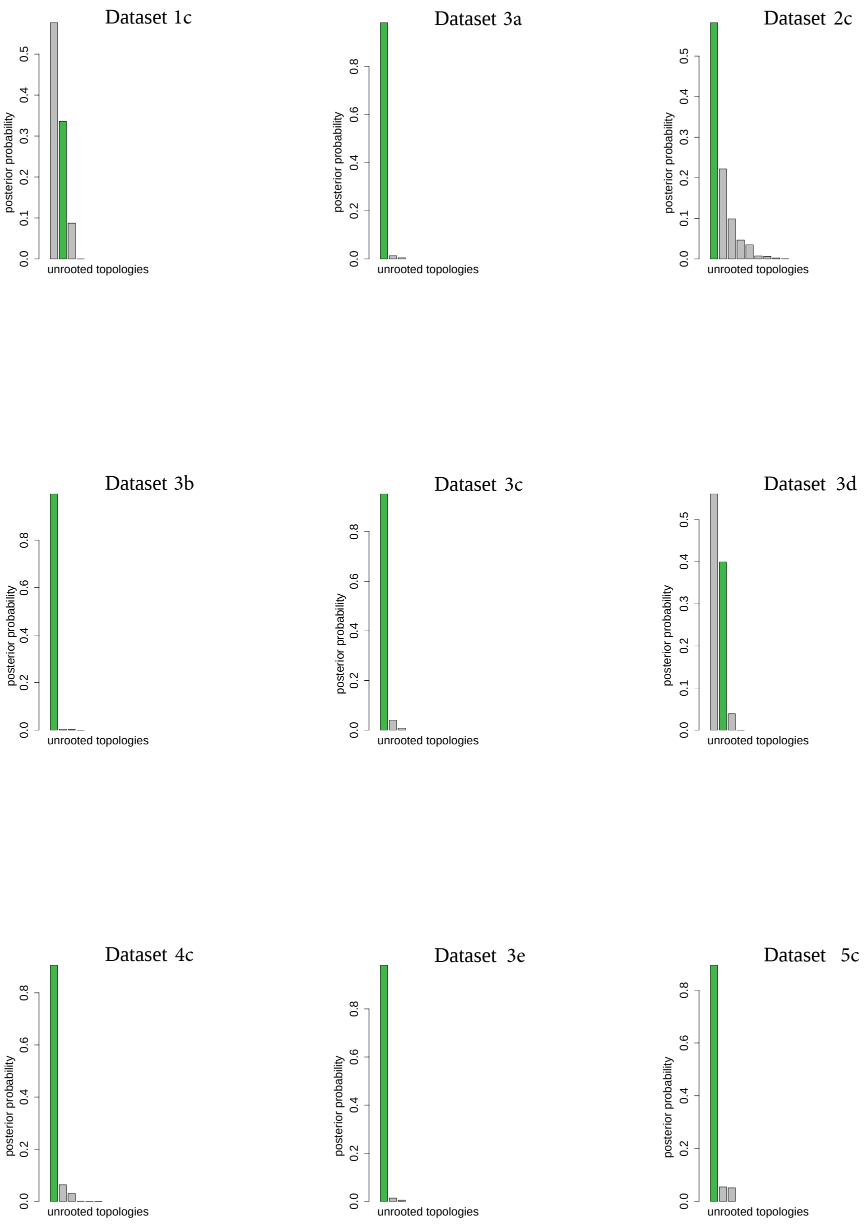

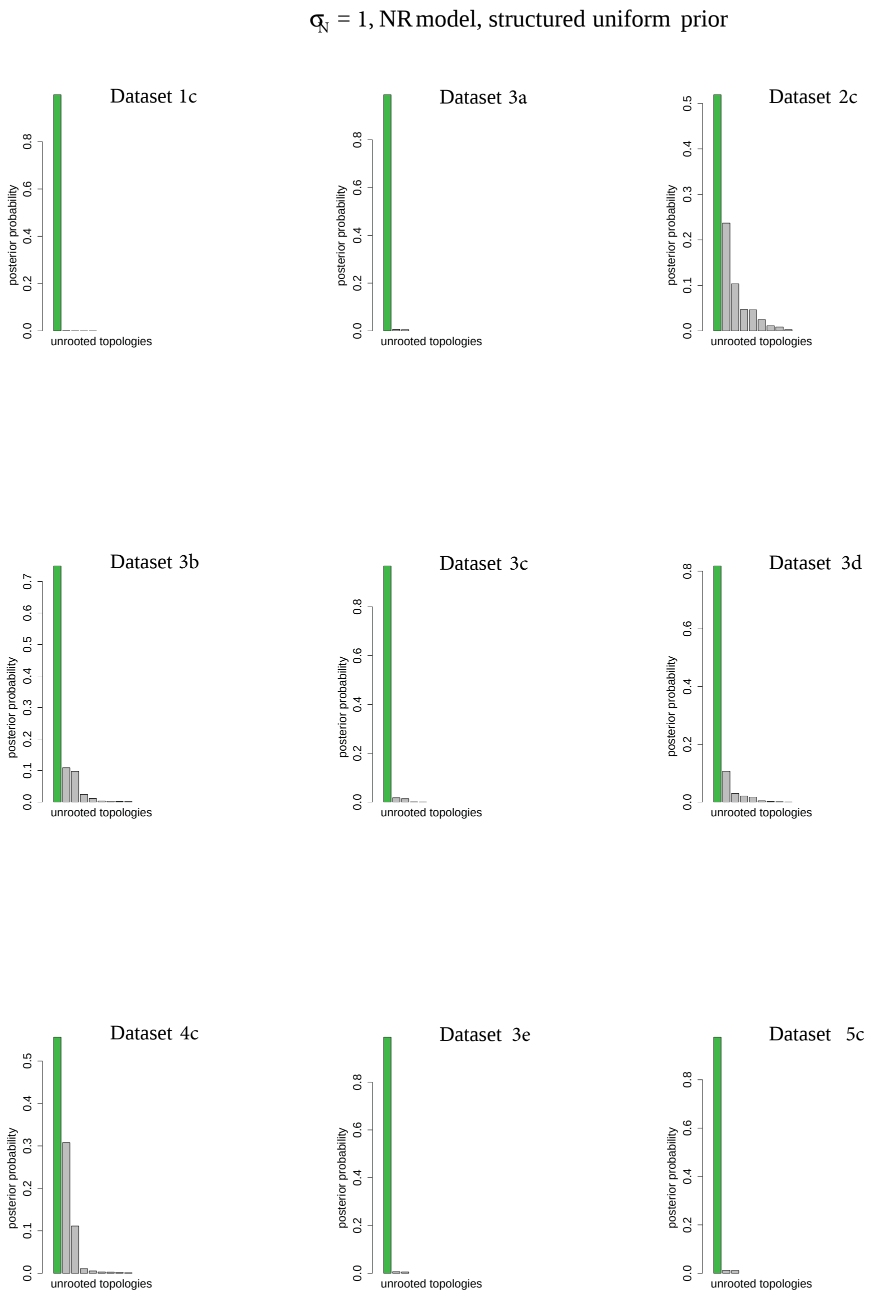

Supplementary Figure 7: Posterior distribution of the unrooted topologies for a representative selection of data sets simulated under the NR2 model with different values of σ_N and analysed with the NR model and structured uniform prior. Different bars on the plots represent different unrooted topologies on the posterior distribution of trees (ordered by posterior probabilities). On each plot the green bar represents the true unrooted topology.

σ_N= 0, NR2 model, Yule prior

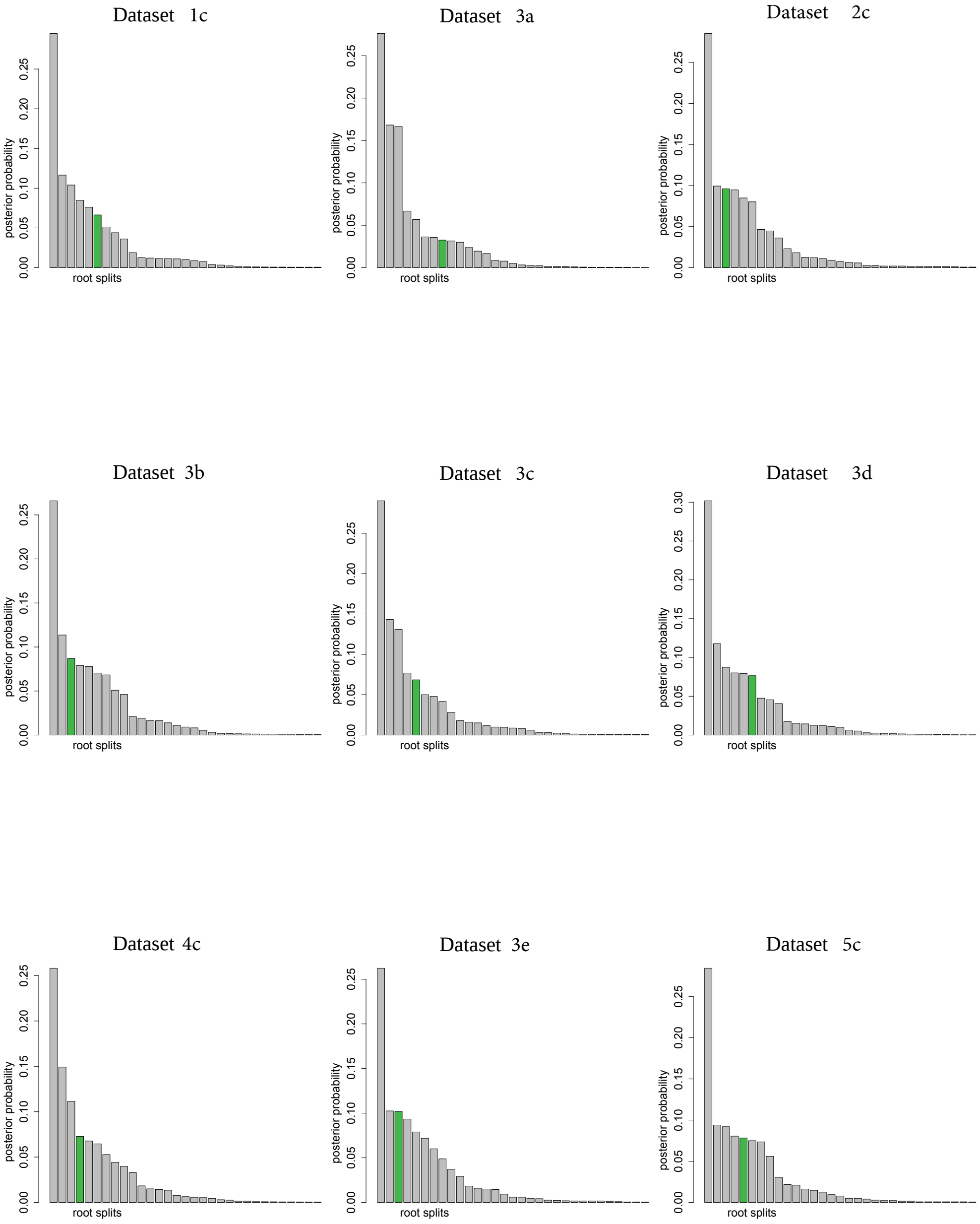

σ_N= 0.1, NR2 model, Yule prior

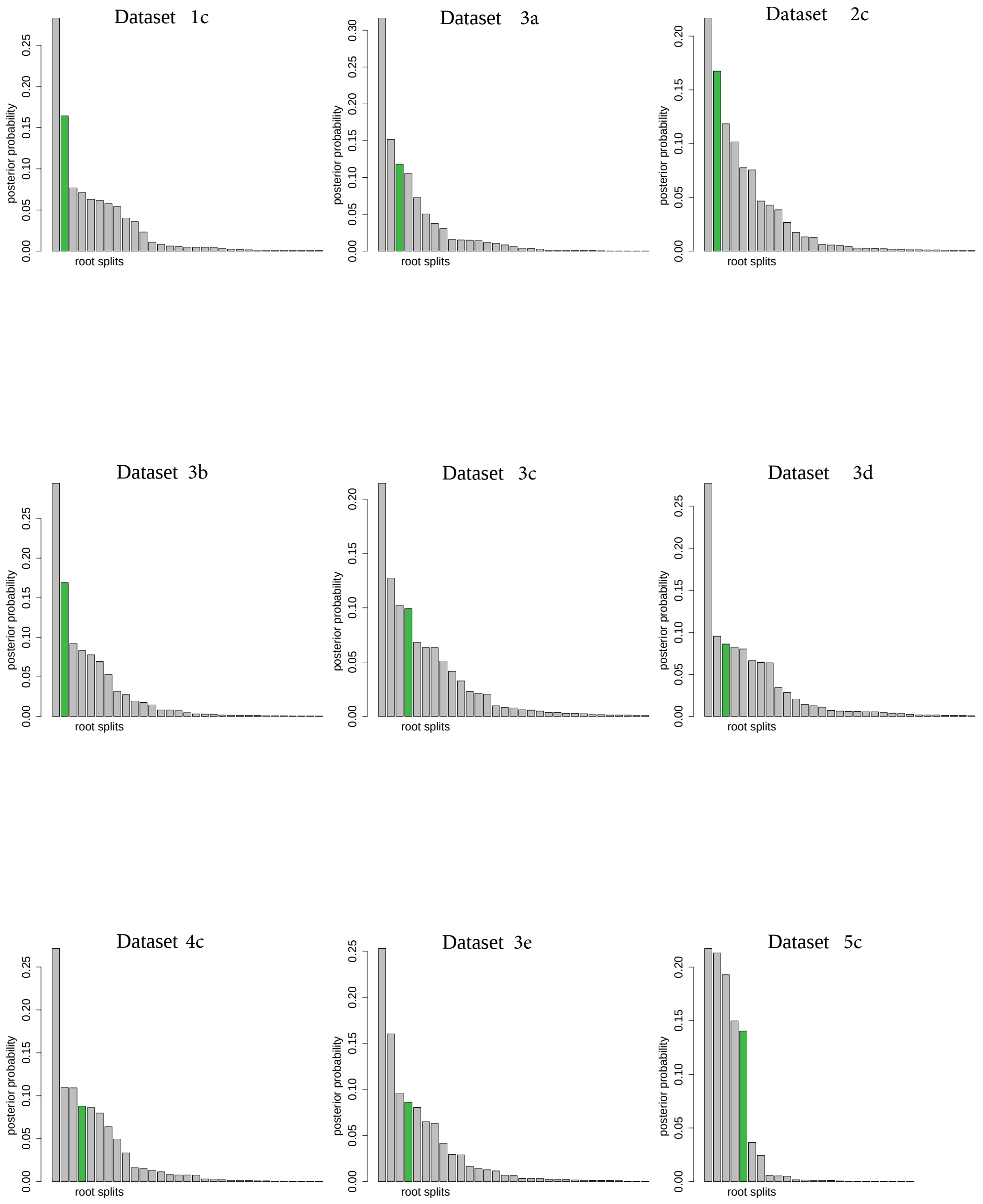

σ_N= 0.25, NR2 model, Yule prior

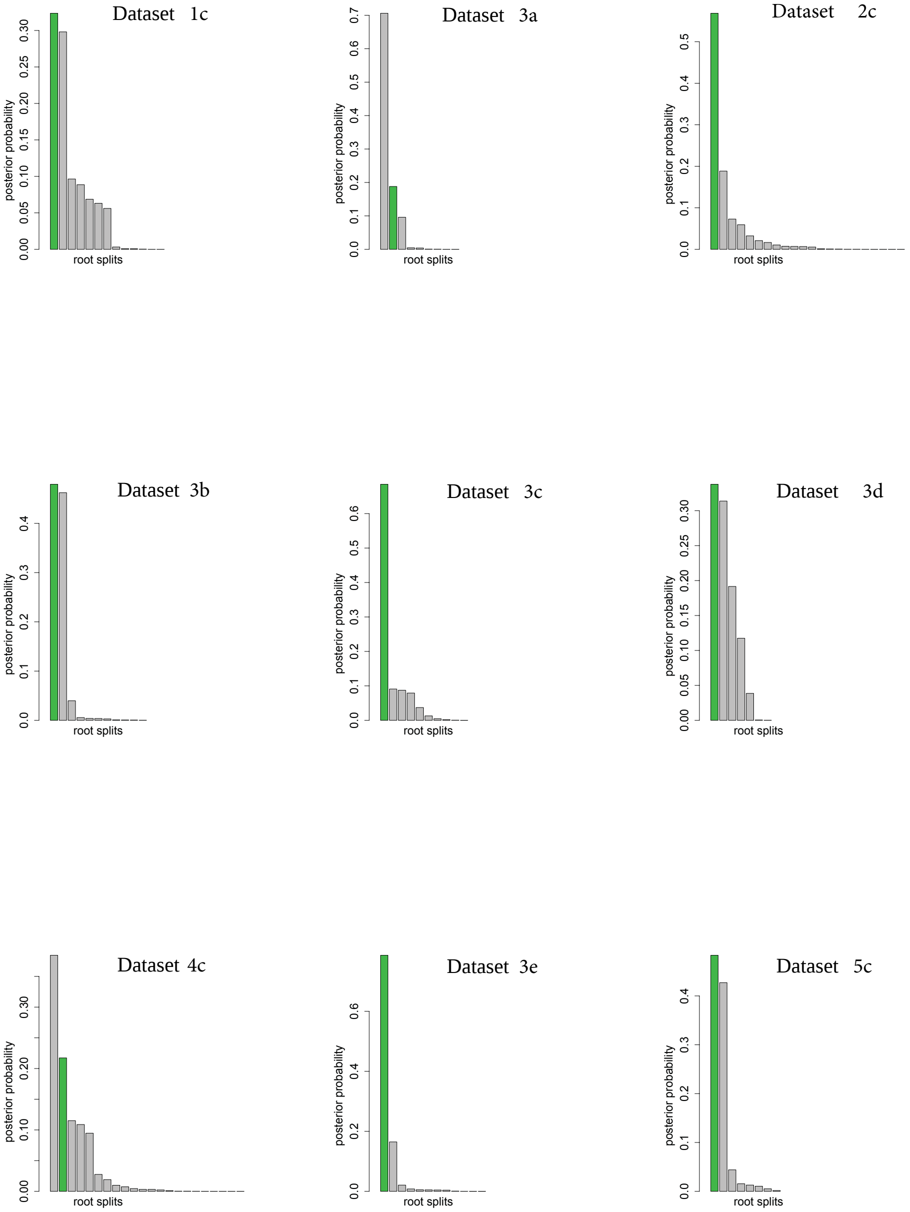

σ_N= 0.5, NR2 model, Yule prior

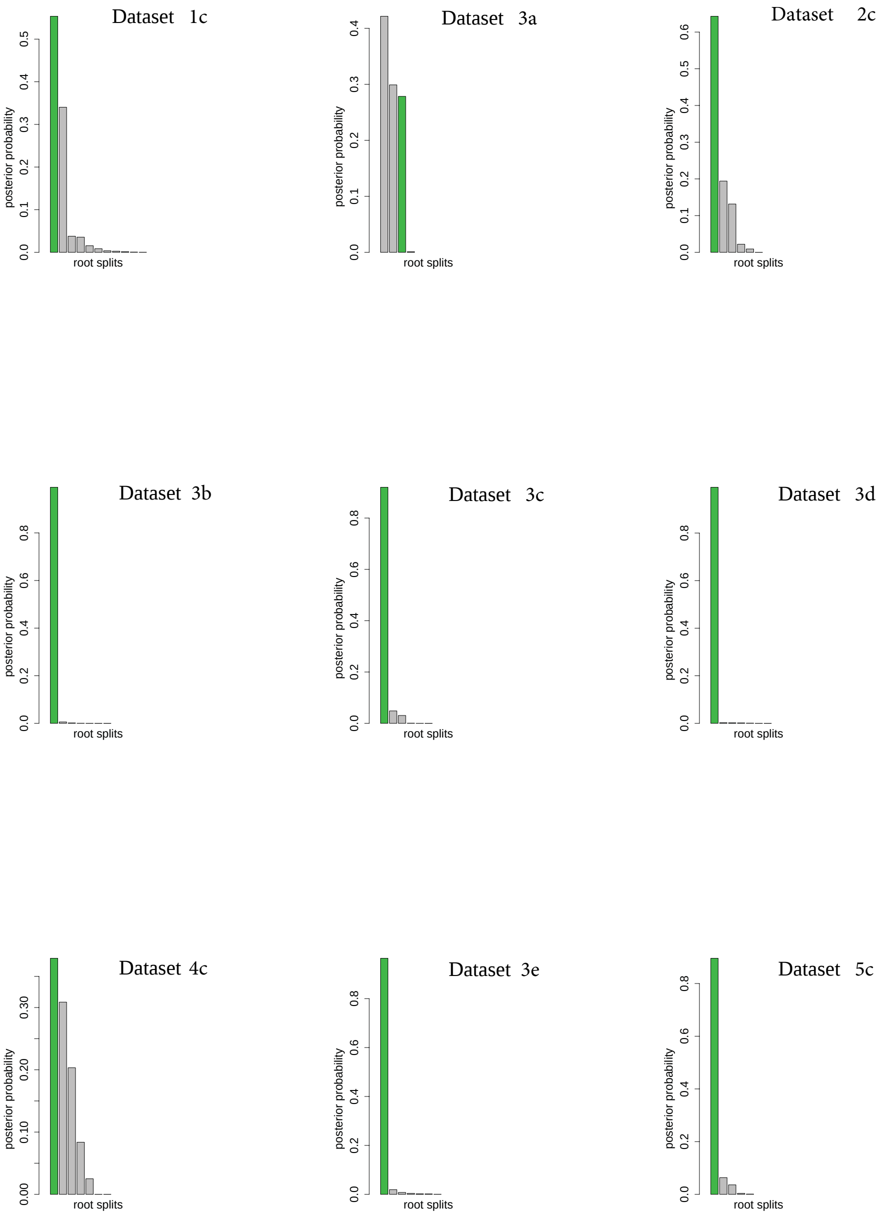

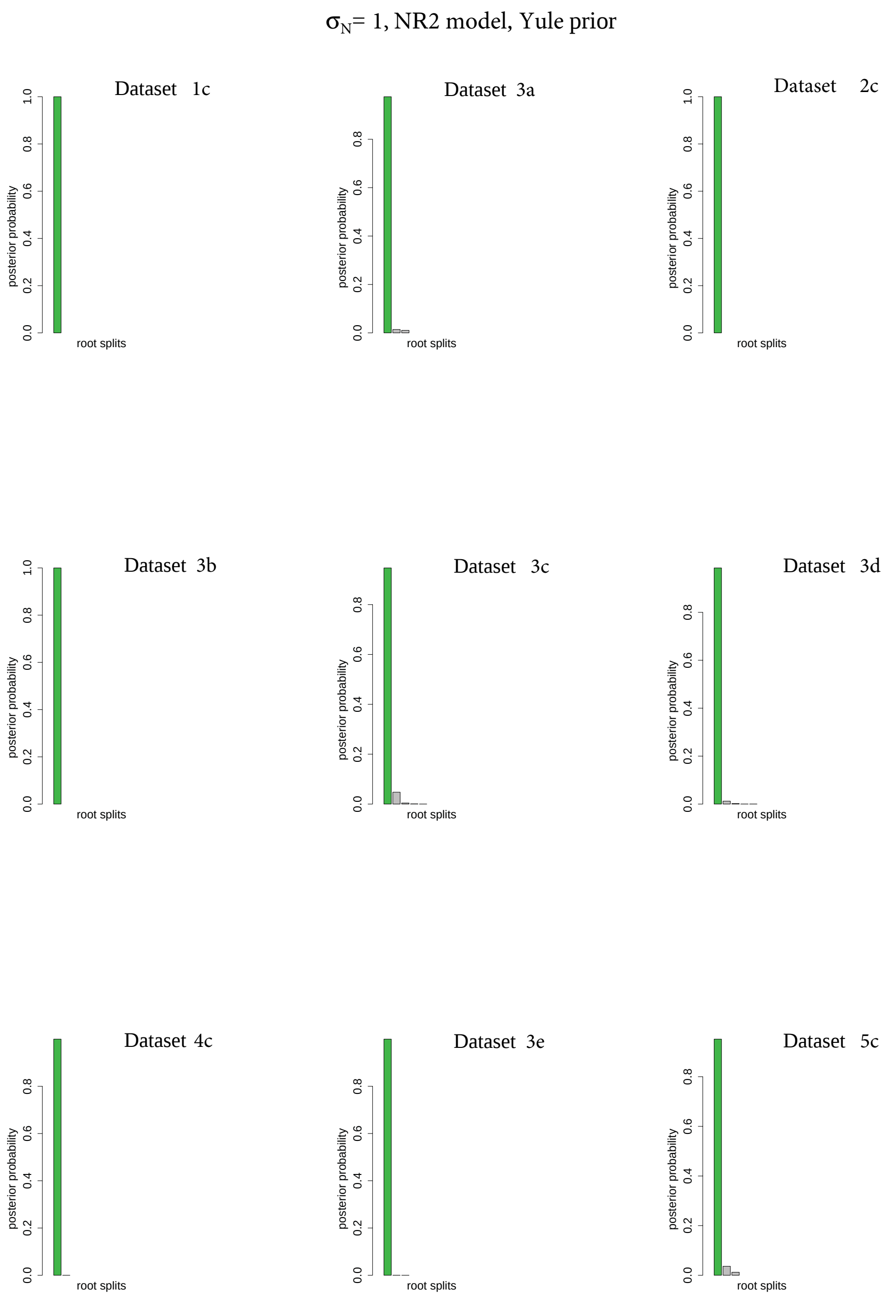

Supplementary Figure 8: Posterior distribution of the root splits for a representative selection of data sets simulated under the NR2 model with different values of σ_N and analysed with the NR2 model and Yule prior. Different bars on the plots represent different root splits on the posterior distribution of trees (ordered by posterior probabilities). On each plot the green bar represents the true root split.

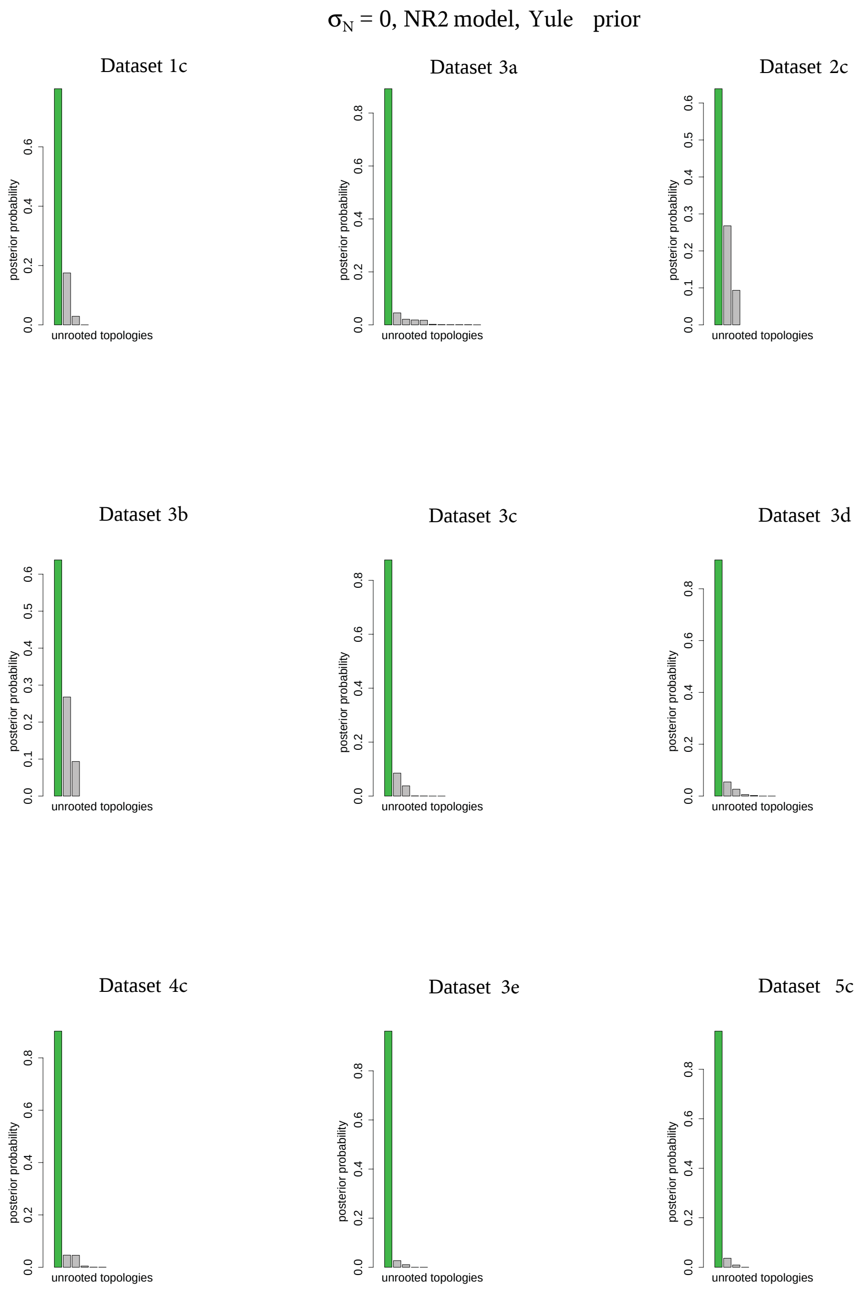
σ_N = 0, NR2 model, Yule prior
Dataset 1c
Dataset 3a
Dataset 2c
Dataset 3b
Dataset 3c
Dataset 3d
Dataset 4c
Dataset 3e
Dataset 5c
posterior probability
unrooted topologies

σ_N = 0.1, NR2 model, Yule prior

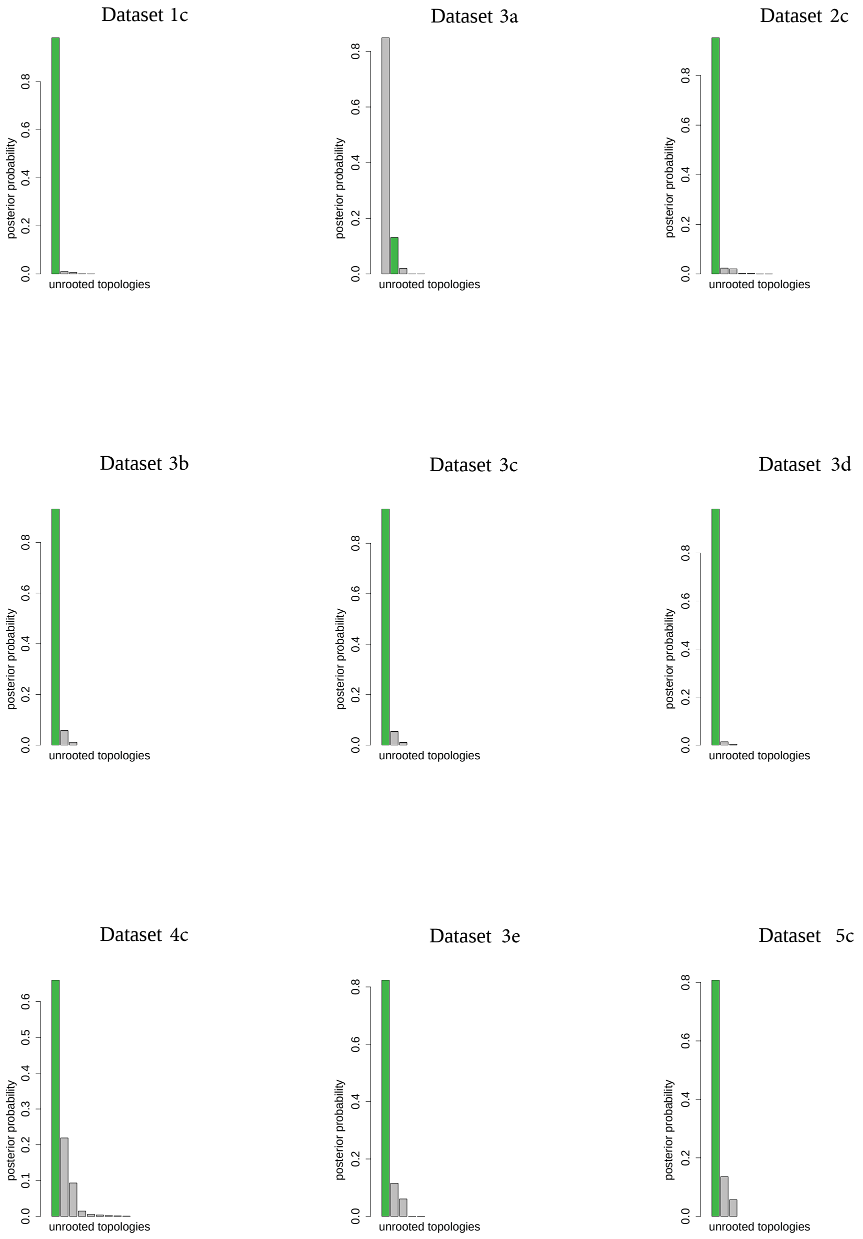

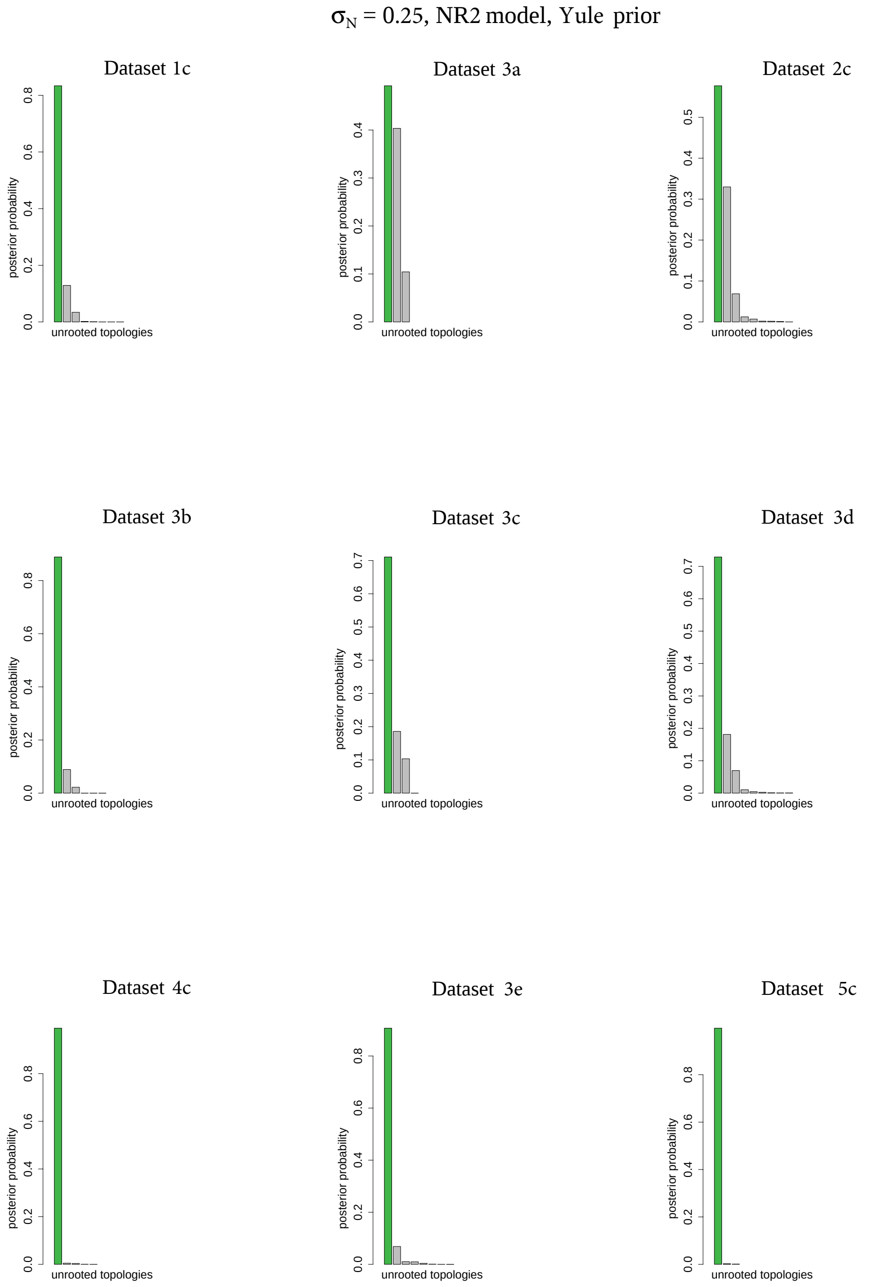
σN = 0.25, NR2 model, Yule prior
Dataset 1c
Dataset 3a
Dataset 2c
Dataset 3b
Dataset 3c
Dataset 3d
Dataset 4c
Dataset 3e
Dataset 5c
posterior probability
unrooted topologies

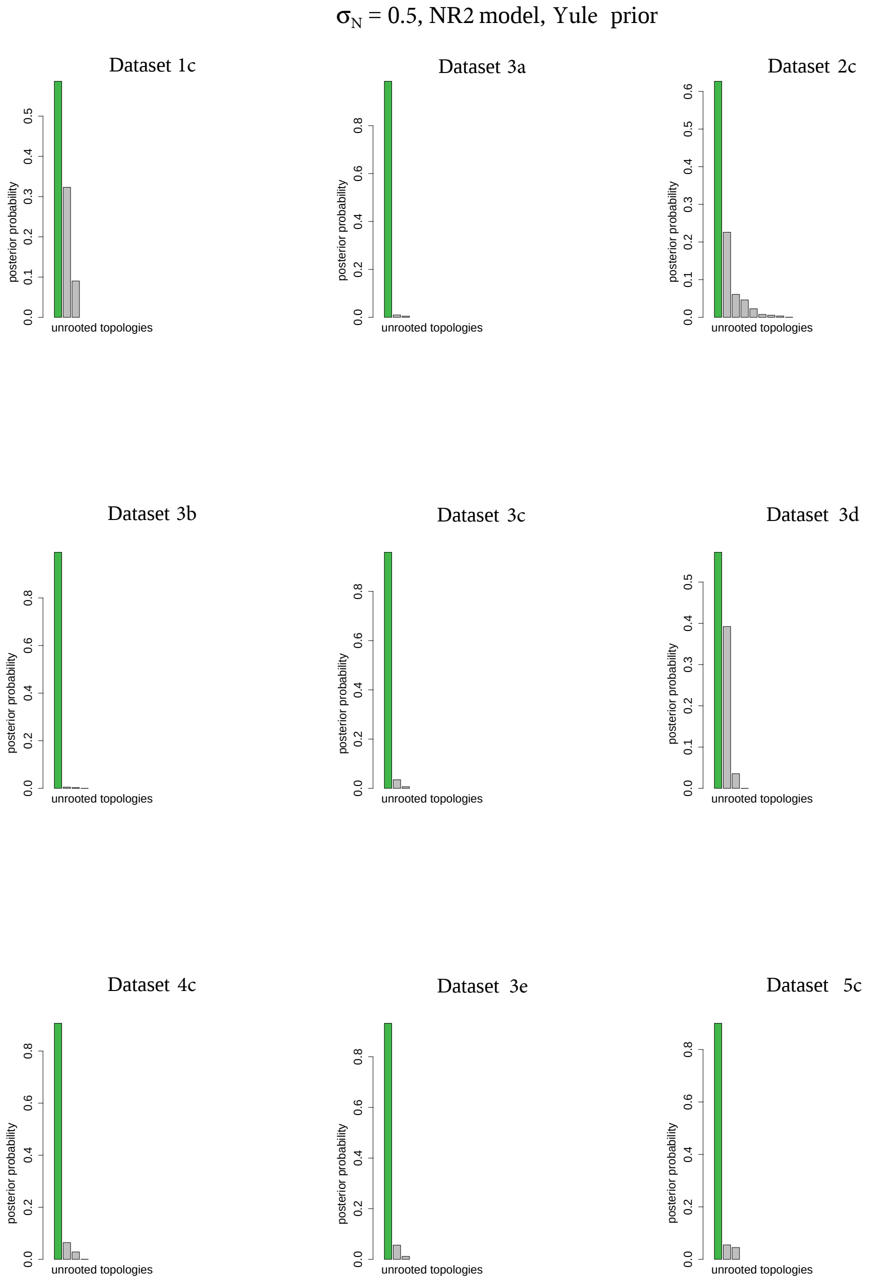
σN = 0.5, NR2 model, Yule prior
Dataset 1c
Dataset 3a
Dataset 2c
Dataset 3b
Dataset 3c
Dataset 3d
Dataset 4c
Dataset 3e
Dataset 5c
posterior probability
unrooted topologies

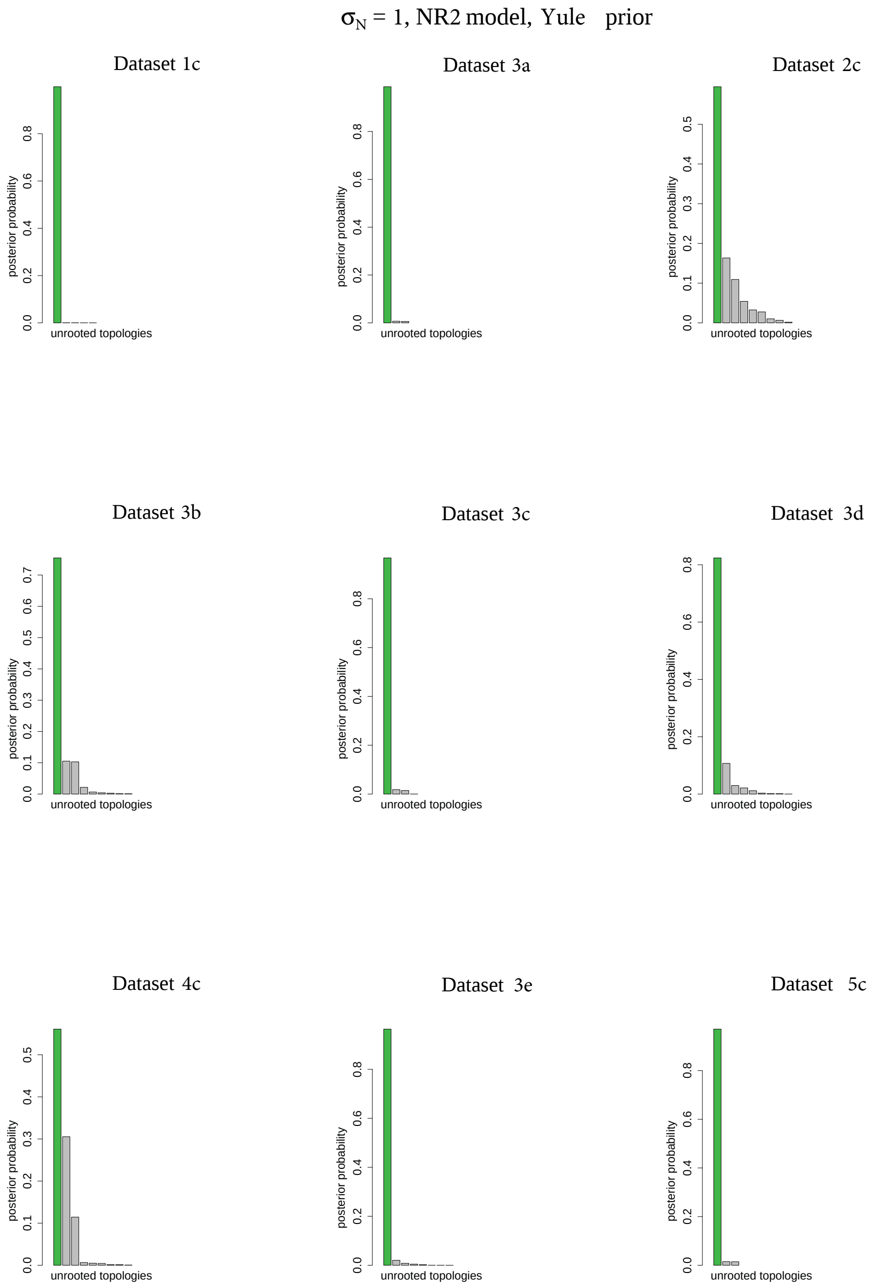

Supplementary Figure 9: Posterior distribution of the unrooted topologies for a representative selection of data sets simulated under the NR2 model with different values of σ_N and analysed with the NR2 model and Yule prior. Different bars on the plots represent different unrooted topologies on the posterior distribution of trees (ordered by posterior probabilities). On each plot the green bar represents the true unrooted topology.

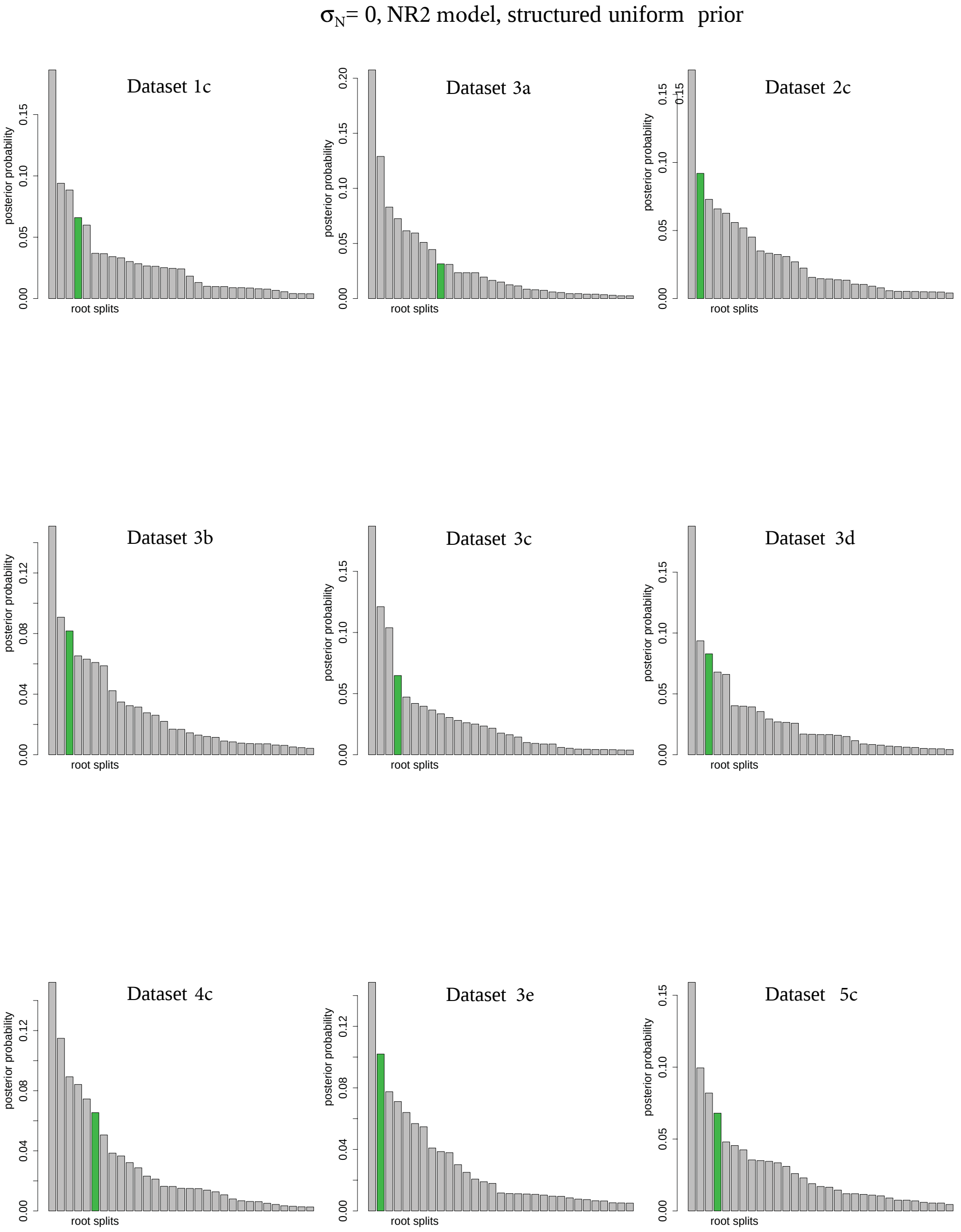
σN= 0, NR2 model, structured uniform prior
Dataset 1c
Dataset 3a
Dataset 2c
Dataset 3b
Dataset 3c
Dataset 3d
Dataset 4c
Dataset 3e
Dataset 5c
posterior probability
root splits

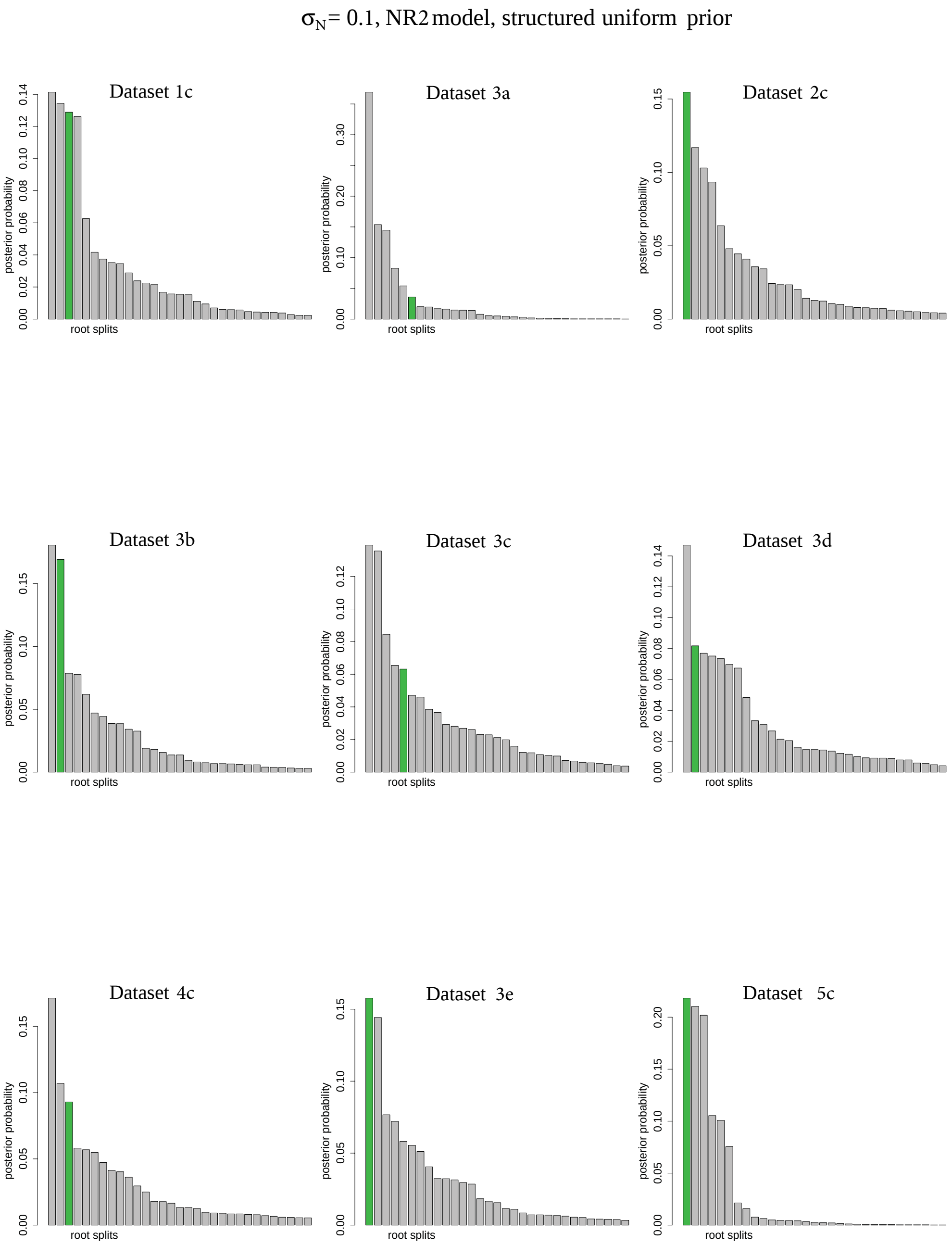
σ_N= 0.1, NR2 model, structured uniform prior
Dataset 1c
Dataset 3a
Dataset 2c
Dataset 3b
Dataset 3c
Dataset 3d
Dataset 4c
Dataset 3e
Dataset 5c
posterior probability
root splits

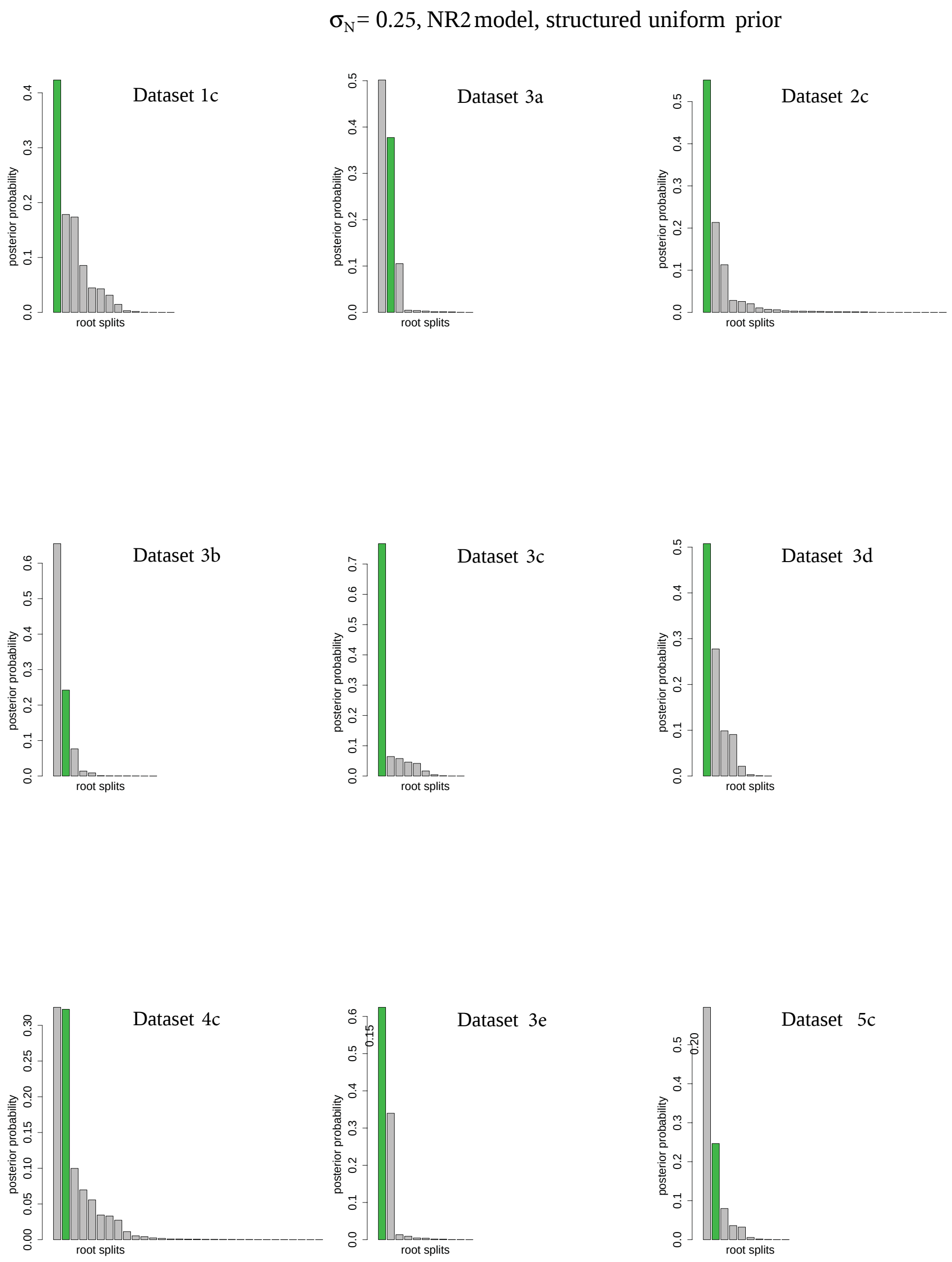
σN= 0.25, NR2 model, structured uniform prior
Dataset 1c
Dataset 3a
Dataset 2c
Dataset 3b
Dataset 3c
Dataset 3d
Dataset 4c
Dataset 3e
Dataset 5c
posterior probability
root splits

σ_N = 0.5, NR2 model, structured uniform prior

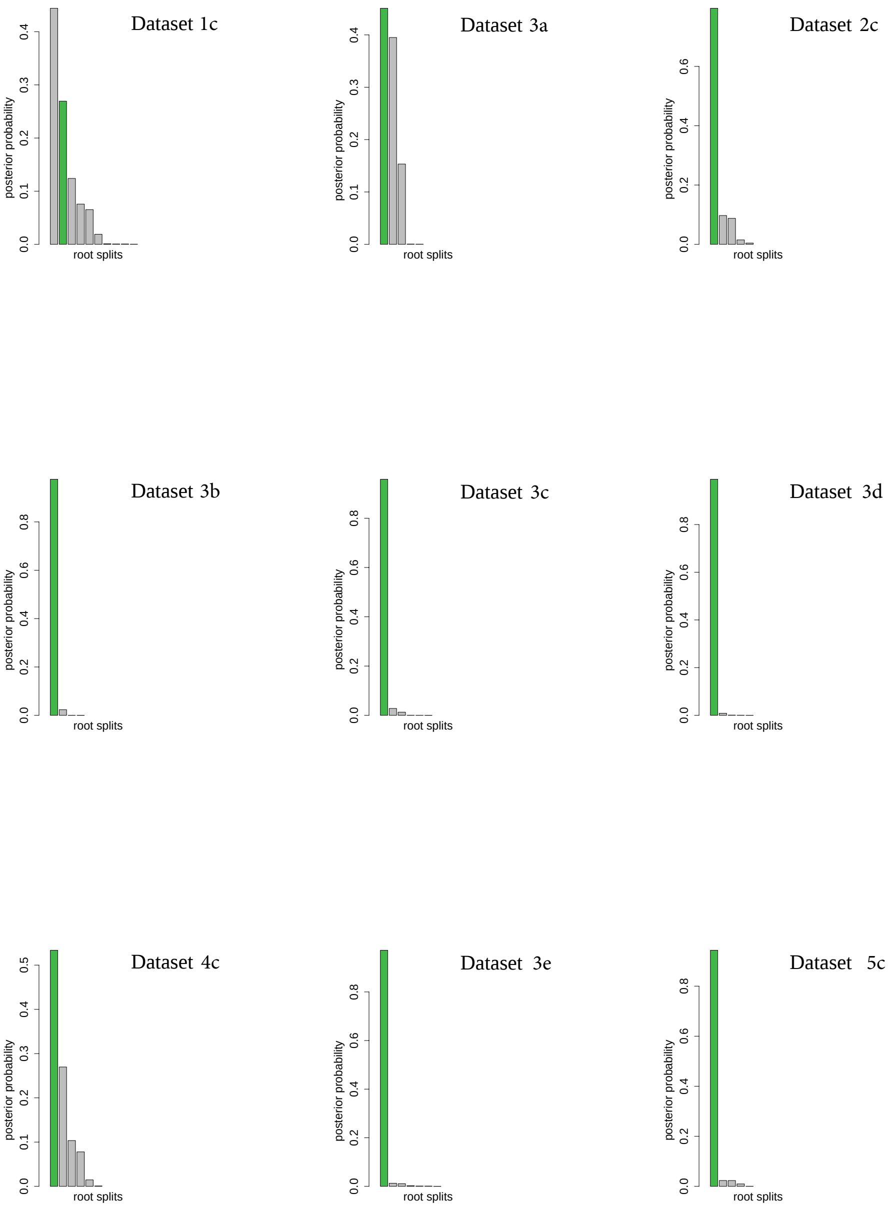

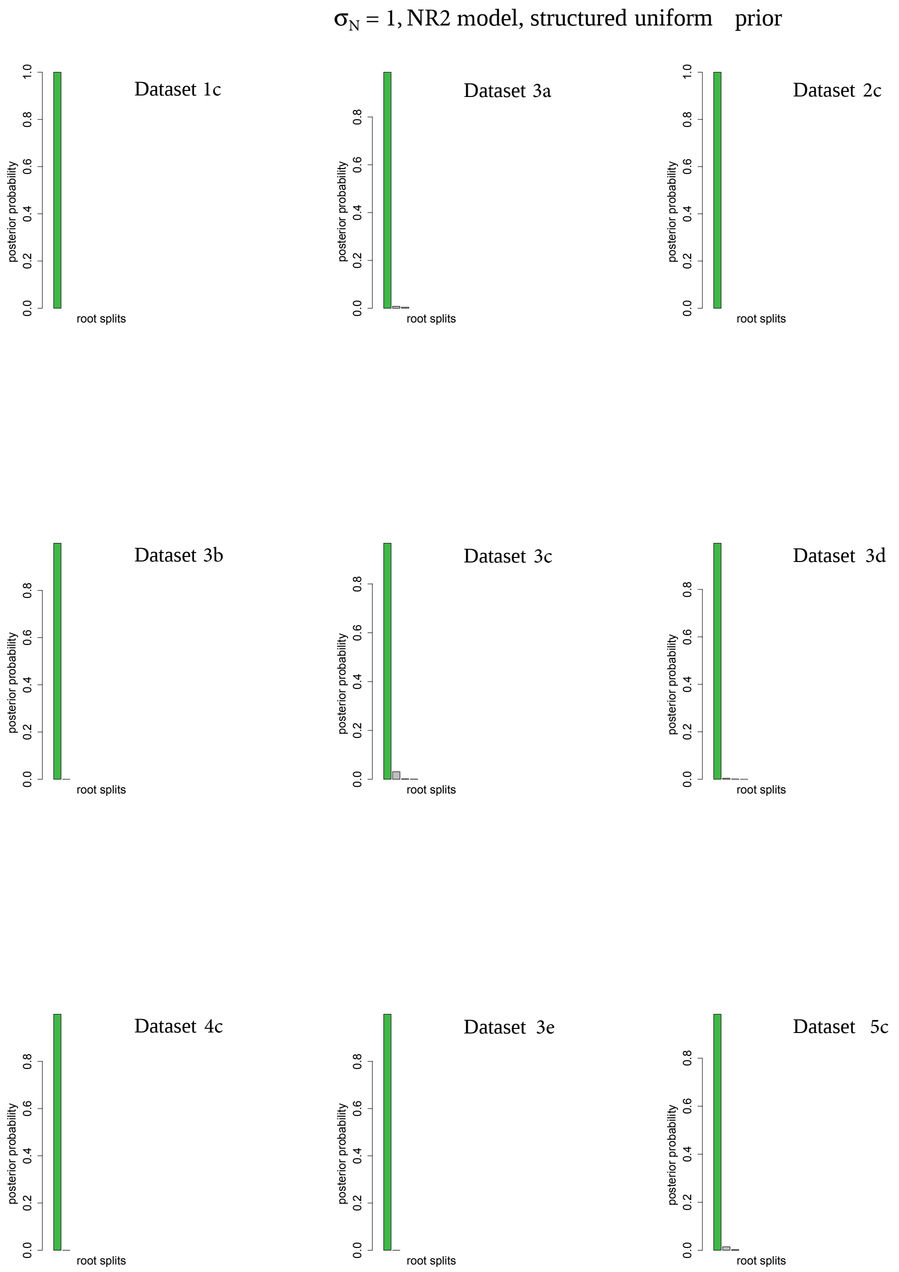

Supplementary Figure 10: Posterior distribution of the root splits for a representative selection of data sets simulated under the NR2 model with different values of σ_N and analysed with the NR2 model and structured uniform prior. Different bars on the plots represent different root splits on the posterior distribution of trees (ordered by posterior probabilities). On each plot the green bar represents the true root split.

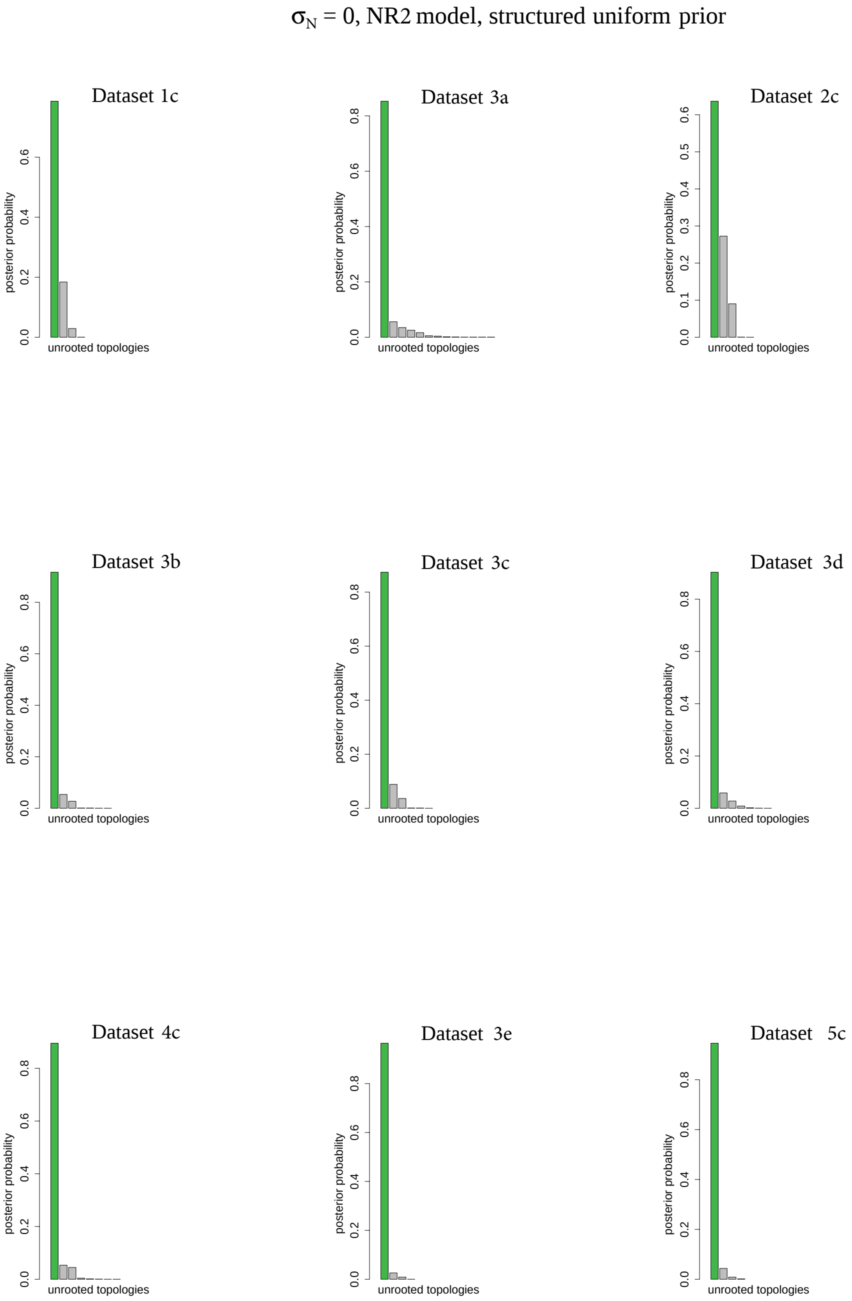
σN = 0, NR2 model, structured uniform prior
Dataset 1c
Dataset 3a
Dataset 2c
Dataset 3b
Dataset 3c
Dataset 3d
Dataset 4c
Dataset 3e
Dataset 5c
posterior probability
unrooted topologies

σ_N = 0.1, NR2 model, structured uniform prior

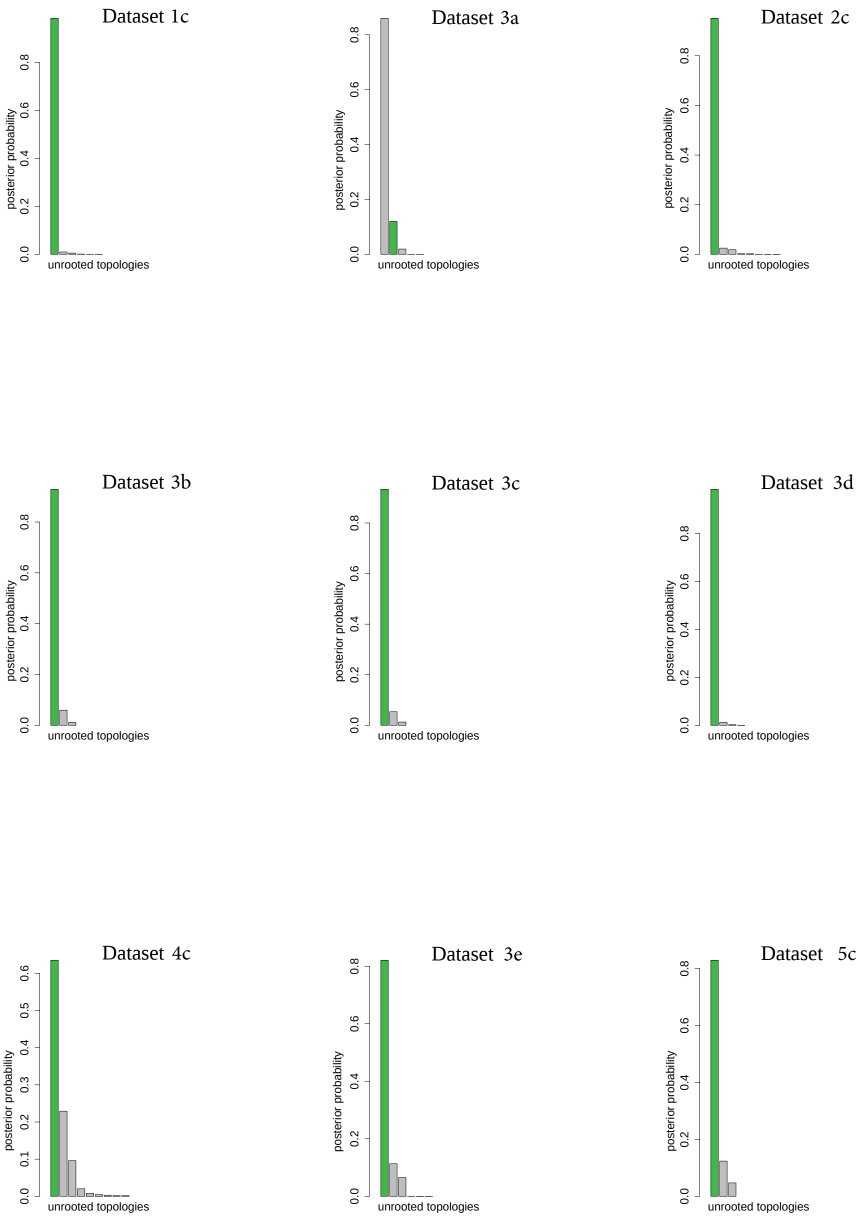

σ_N = 0.25, NR2 model, structured uniform prior

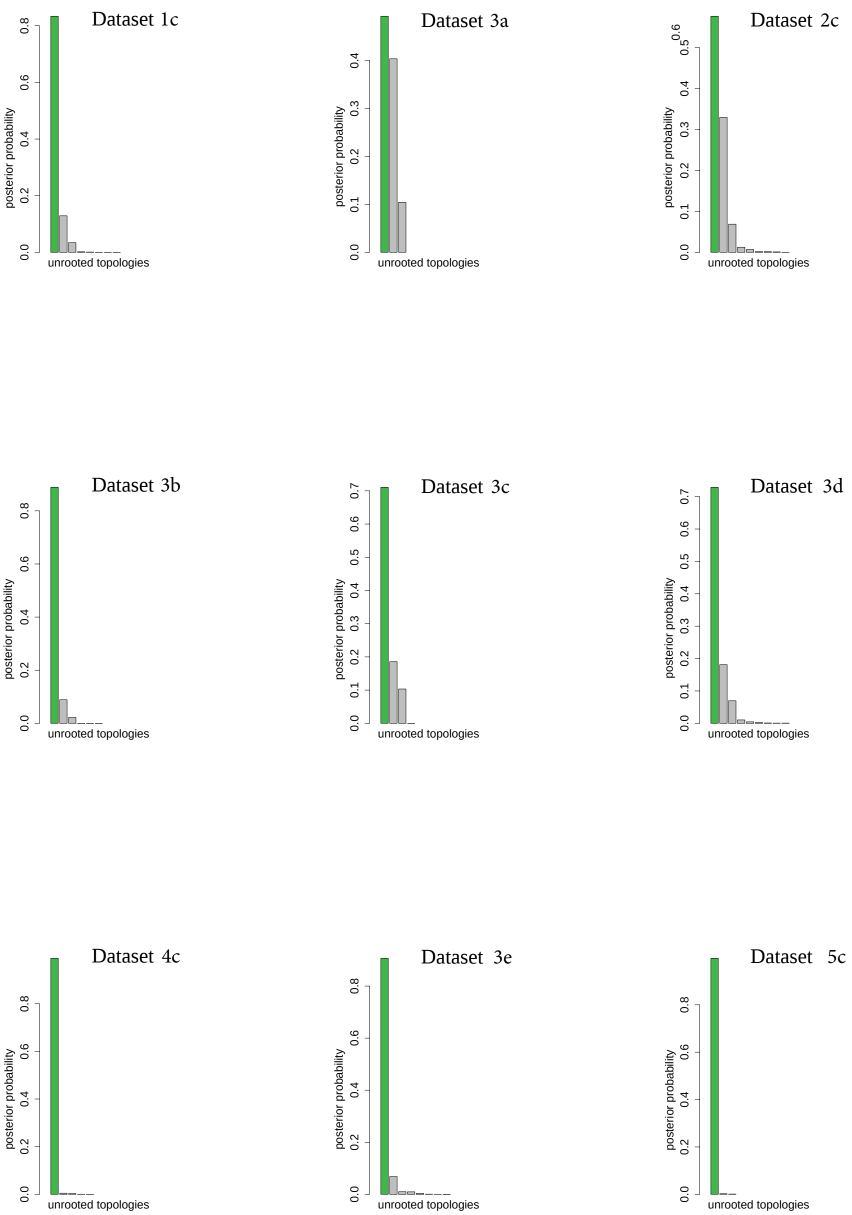

σ_N = 0.5, NR2 model, structured uniform prior

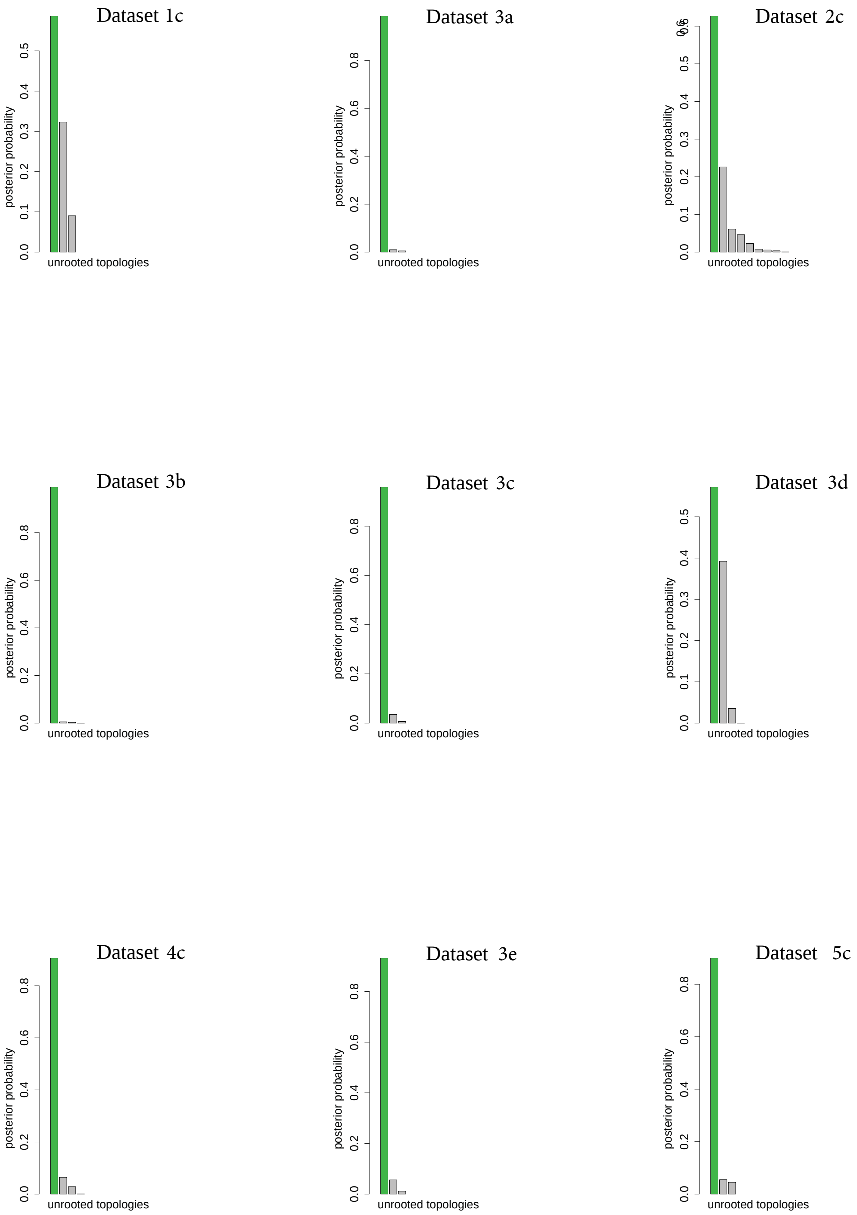

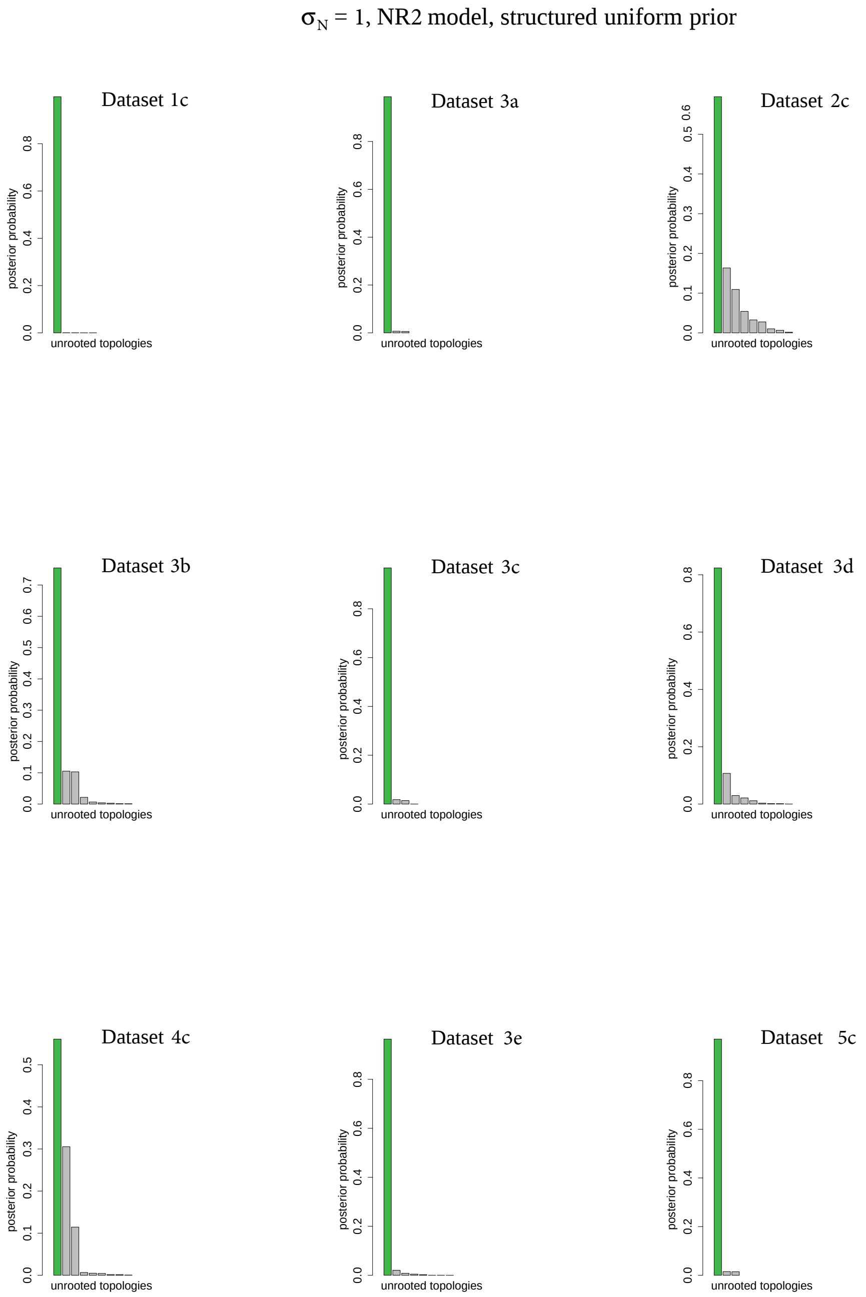

Supplementary Figure 11: Posterior distribution of the unrooted topologies for a representative selection of data sets simulated under the NR2 model with different values of σ_N and analysed with the NR2 model and structured uniform prior. Different bars on the plots represent different unrooted topologies on the posterior distribution of trees (ordered by posterior probabilities). On each plot the green bar represents the true unrooted topology.

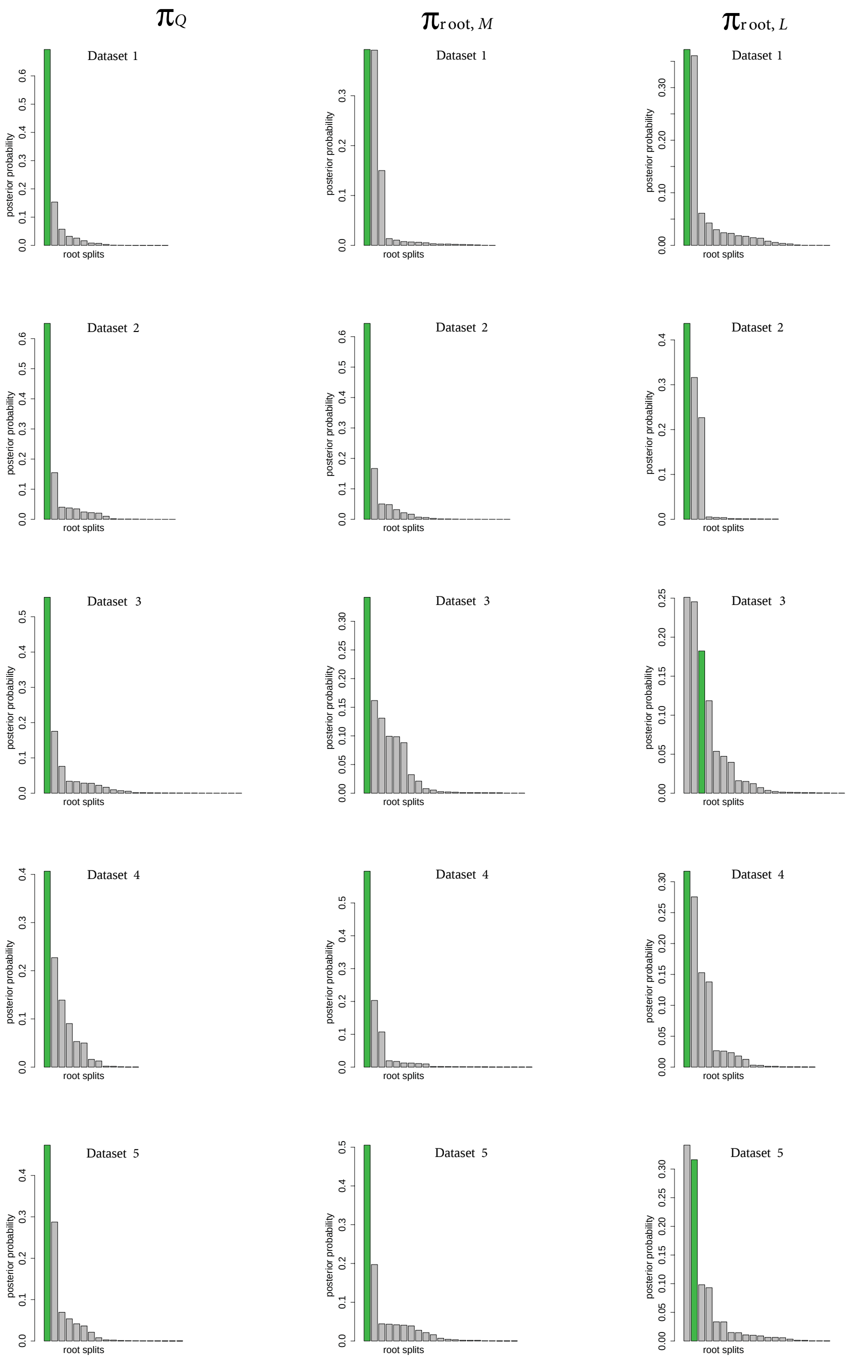

Supplementary Figure 12: Posterior distribution of the root splits for the data sets simulated under the non-stationary variant of the NR model, analysed using the NR model, with stationary distribution $\boldsymbol{\pi}_Q$, and the Yule prior. The distributions at the root are $\boldsymbol{\pi}_Q$ and two distributions, $\boldsymbol{\pi}_{\mathrm{root},M}$ and $\boldsymbol{\pi}_{\mathrm{root},L}$, increasingly displaced from $\boldsymbol{\pi}_Q$. Different bars on the plots represent different root splits on the posterior distribution of trees (ordered by posterior probabilities). On each plot the green bar represents the true root split.

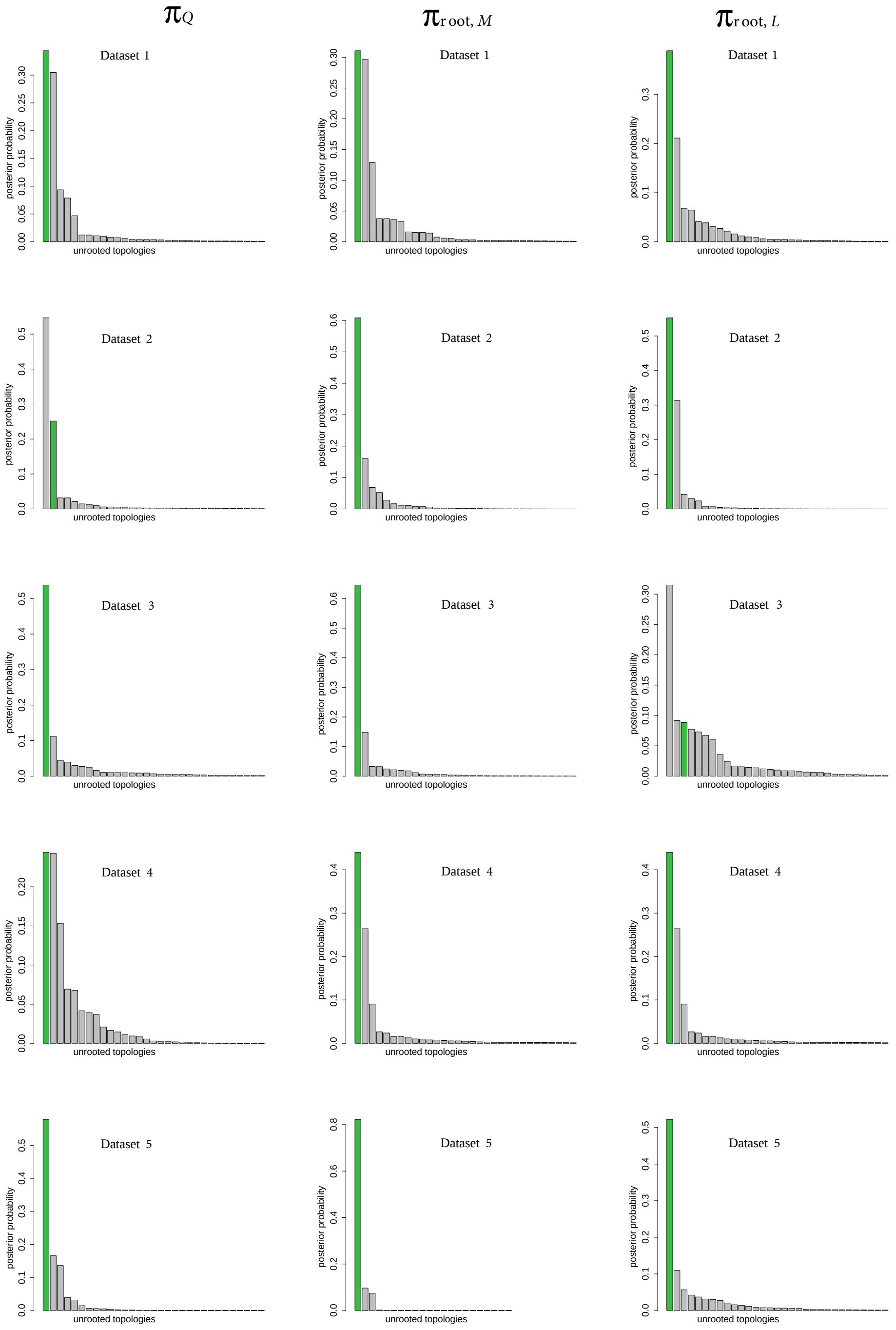

Supplementary Figure 13: Posterior distribution of the unrooted topologies for the data sets simulated under the non-stationary variant of the NR model, analysed using the NR model, with stationary distribution $\boldsymbol{\pi}_Q$, and the Yule prior. The distributions at the root are $\boldsymbol{\pi}_Q$ and two distributions, $\boldsymbol{\pi}_{\text{root},M}$ and $\boldsymbol{\pi}_{\text{root},L}$, increasingly displaced from $\boldsymbol{\pi}_Q$. Different bars on the plots represent different unrooted topologies on the posterior distribution of trees (ordered by posterior probabilities). On each plot the green bar represents the true unrooted topology.

References

Chib, S. 1995. Marginal likelihood from the Gibbs output. *J. Amer. Statist. Assoc.*, 90: 1313–1321.
